# Supplementary material for: A stem-like chromatin program in small-cell lung cancer is associated with poor outcomes after chemoimmunotherapy
Source: Cell Rep Med. 2026 Sep 10;7(10):103027. doi: 10.1016/j.xcrm.2026.103027 (PMC13710148; doi:10.1016/j.xcrm.2026.103027)
Supplement: Supplementary file 1 [file mmc1.pdf]

Cell Reports Medicine, Volume *7*

Supplemental information

A stem-like chromatin program in small-cell lung cancer is associated with poor outcomes after chemoimmunotherapy

Rajesh Kumar, Parth Desai, Rajaa El Meskini, Chirayu Mohindroo, Alexandra Ágnes Benő, Devon Atkinson, Michael Nirula, Laura Bassel, Nobuyuki Takahashi, Kelly L. Sovacool, Anna Meinhardt, Mohit Arora, Kanak Parmar, Darryl Nausome, Samantha Nichols, Linda Sciuto, Shannon Swift, Kolos Nemes, Ajit Kumar Sharma, Nathan Pate, Arun Rajan, Elliot Levy, Michael Kassin, Christopher Febres Aldana, Vishal Koparde, Melissa Galloux, Roshan Shreshta, Zoe Weaver Ohler, Lorinc Pongor, and Anish Thomas

Fig. S1

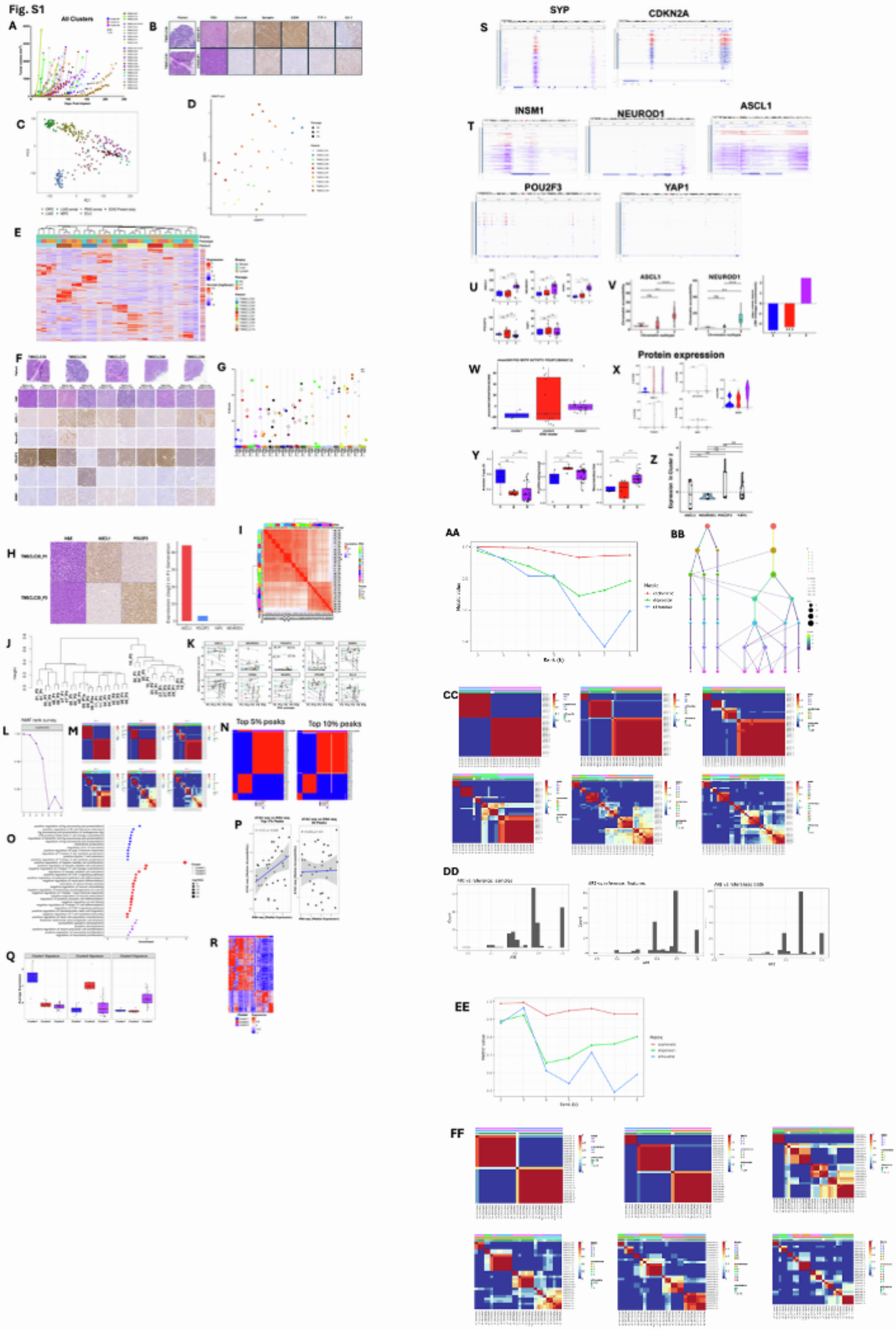

A
All Clusters
B
C
D
E
F
G
H
H&E
ASCL1
POU2F3
I
J
K
L
M
N
Top 5% peaks
Top 10% peaks
O
P
Q
R
S
SYP
CDKN2A
T
INSM1
NEUROD1
ASCL1
POU2F3
YAP1
U
V
ASCL1
NEUROD1
W
X
Protein expression
Y
Z
AA
BB
CC
DD
EE
FF

Figure S1. Validation of PDX models and stability of chromatin accessibility-informed regulatory programs and NMF clustering in SCLC, related to Figure 1

A. Growth kinetics of PDX tumors.
B. H/E and IHC analysis of key SCLC diagnostic markers (chromogranin A, synaptophysin, CD56, TTF-1, and/or CK-7) in patient-derived tumors and their corresponding first-generation PDX tumors (P1). For further details, see Tables S1 and S2. Scale bars, 100 µm.
C. Pan-cancer rPCA projection of transcriptomic data from the current study, co-clustering with known SCLC tumors[28].
D. UMAP of transcriptomic data (top 1% most variable genes), showing co-localization of P0 and corresponding PDX tumors.
E. Heatmap generated after removal of TME genes (using an approach described previously[29]) displaying co-clustering of P0 and PDX tumors.
F. H/E staining of patient-origin tumors (on top) and corresponding PDX tumors (P1, P3), alongside IHC of key SCLC lineage-defining markers (ASCL1, NEUROD1, INSM1, YAP1, and POU2F3) across P1 and P3. See Table S3 for more details. Scale bars, 100 µm.
G. IHC quantification (H-scores) of ASCL1, NEUROD1, INSM1, YAP1, and POU2F3 in PDX tumors (P1, P3).
H. IHC staining of TMSCLC20 shows a passage-associated shift from ASCL1-high at P1 to POU2F3-high at P3 by IHC, with P1 bulk RNA-seq confirming high ASCL1 expression.
I. Pearson correlation heatmap of bulk RNA-seq profiles from patient-derived xenografts (PDXs) across multiple passages. Samples from different passages of same PDX cluster together with some variability, indicating global preservation of global transcriptional identity.
J. Hierarchical clustering showing multiple passages from the same PDX frequently co-cluster, while broader branch structure reflects inter-tumor heterogeneity, supporting the absence of global transcriptional drift during PDX propagation.
K. Expression pattern of core SCLC marker conserved among the serial passages across the matched PDX samples.
L. Cophenetic correlation index of NMF for ATAC-seq data (top 1% most variable peaks) at various k values.
M. Cross-correlation map of ATAC-seq data at various NMF resolutions, demonstrating that k=3 is the optimal resolution.
N. NMF subtype assignments are stable in PDX using the top 5% vs 10% most variable ATAC-seq peaks.
O. Chromatin-accessibility defined cluster exhibits distinct biological programs enriched for neuronal, immune and stemness pathways.
P. Correlation plot showing that concordance between the ATAC-seq and matched RNA-seq dataset for top 1% peaks (left) and all peaks (right).
Q. Boxplot depicting the specificity of genes linked to chromatin-accessibility derived peaks. Center line indicates the median; box limits indicate the first and third quartiles; whiskers represent 1.5 × IQR; points represent individual samples.
R. Heatmap showing specificity of genes linked to chromatin-accessibility derived peaks.
S. Chromatin accessibility of core SCLC markers SYP and CDKN2A.
T. Chromatin accessibility of established SCLC lineage-defining markers ASCL1, NEUROD1, YAP1, POU2F3 and INSM1.
U. Quantification of chromatin accessibility of established SCLC lineage-defining markers ASCL1, NEUROD1, YAP1, POU2F3 and INSM1. Center line indicates the median; box limits indicate the first and third quartiles; whiskers represent 1.5 × IQR; points represent individual samples.
V. Plot depicting the enrichment of ASCL1/ NEUROD1 and odd ratio enrichment of SCLC lineage markers in Cluster 3 compared to Cluster 1 and Cluster 2. Center line indicates the median; box limits indicate the first and third quartiles; whiskers represent 1.5 × IQR; points represent individual samples.
W. Motif enrichment of chromatin defined cluster reveals heterogeneous expression of POU2F3 within Cluster 2. Center line indicates the median; box limits indicate the first and third quartiles; whiskers represent 1.5 × IQR; points represent individual samples.
X. IHC quantification (H-scores) for INSM1, ASCL1, NEUROD1, POU2F3, and YAP1. Center line indicates the median; box limits indicate the first and third quartiles; whiskers represent 1.5 × IQR; points represent individual samples.

Y. Boxplot depicting enrichment of cell-type specific signature in chromatin defined clusters. Center line indicates the median; box limits indicate the first and third quartiles; whiskers represent 1.5 × IQR; points represent individual samples.

Z. Plot showing expression of canonical SCLC lineage markers (ASCL1, NEUROD1, POU2F3, and YAP1) in chromatin-defined Cluster 2. Center line indicates the median; box limits indicate the first and third quartiles; whiskers represent 1.5 × IQR; points represent individual samples.

AA. Cophenetic correlation, dispersion, and silhouette scores for NMF solutions on ATAC-seq data (top 1% most variable peaks) across values of k (n=39).

BB. Clustree analysis showing stable cluster lineage from k=2 to k=3, with increased fragmentation at higher k in PDX ATAC-seq dataset.

CC. Consensus cross correlation similarity heatmaps for NMF solutions at different k values, illustrating that k = 3 yields the most stable and well-separated clusters.

DD. Perturbation-based bootstrap stability analysis of the NMF derived k=3. Histograms show the distribution of Adjusted Rand Index (ARI) values from 200 bootstrap replicates under three perturbation condition.

EE. Plot showing cluster stability metrices (cophenetic, dispersion and silhouette) on the discordant sample removed dataset (n=28) across different values of k.

FF. Heatmap showing cluster stability at different values of NMF (k=2-8). k=3 is most stable with well-defined cluster stability blocks and increasing sub clustering yield off-diagonal signal, showing reduced cluster stability.

NMF, nonnegative matrix factorization; PCA, principal component analysis; TME, tumor microenvironment; UMAP, Uniform manifold approximation and projection; IHC, Immunohistochemistry *p<0.05, *** p<0.001, Tukey’s multiple comparison test.

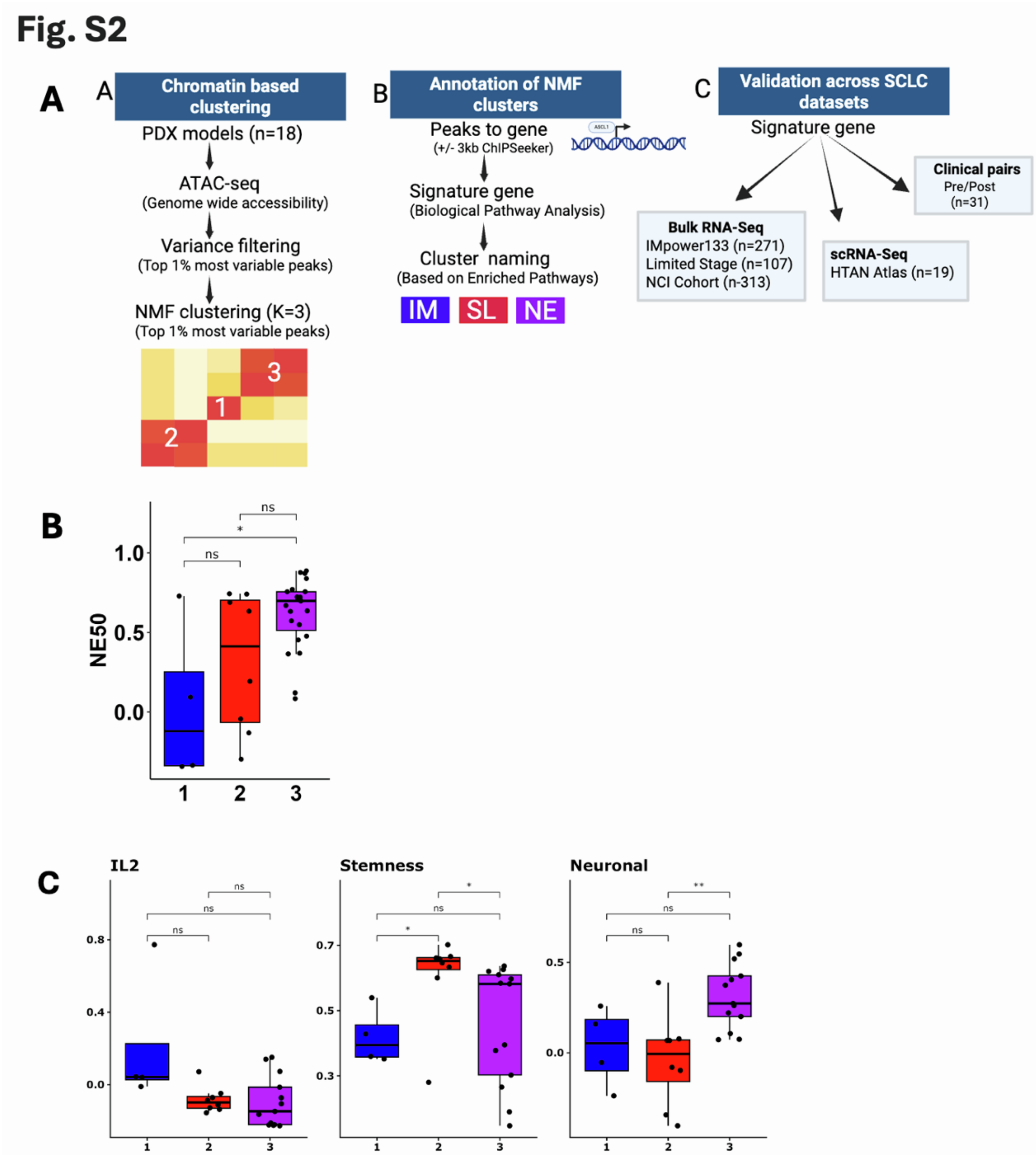

Figure S2. Computational framework and transcriptomic features supporting chromatin-defined SCLC regulatory programs, related to Figure 2

A. Concept schematic figure describing the overall computational framework for clustering, naming and cross validation strategy used in the study
B. Boxplot depicting expression of a 50-gene NE signature[15] across the three ATAC-seq-derived clusters in PDX transcriptomic dataset (n=35). Center line indicates the median; box limits indicate the first and third quartiles; whiskers represent 1.5 × IQR; points represent individual samples.

C. Pathway activity using ssGSEA analysis, showing upregulation of IL-2 signaling in cluster1, stemness signature in cluster2 and neuronal signaling in cluster3 samples. Center line indicates the median; box limits indicate the first and third quartiles; whiskers represent 1.5 × IQR; points represent individual samples. Tukey's multiple comparison test; * $p < 0.05$, ** $p < 0.005$, *** $p < 0.0001$

NE, neuroendocrine

Fig. S3

A **SCLC-IM**

Total Target Sequences = 35352, Total Background Sequences = 31271

Rank	Motif	Name	P-value	log P-pvalue	q-value (Benjamini)	# Target Sequences with Motif	% of Targets Sequences with Motif	# Background Sequences with Motif	% of Background Sequences with Motif
1		KLF10(Zf)/HEK293-KLF10.GFP-ChIP-Seq(GSE58341)/Homer	1e-573	-1.320e+03	0.0000	6024.0	17.04%	2658.0	8.50%
2		IRF8(IRF)/BMDM-IRF8-ChIP-Seq(GSE77884)/Homer	1e-128	-2.970e+02	0.0000	2185.0	6.18%	1111.7	3.56%
3		bZIP:IRF(bZIP,IRF)/Th17-BatF-ChIP-Seq(GSE39756)/Homer	1e-124	-2.864e+02	0.0000	3276.0	9.27%	1887.0	6.04%
4		Fra1(bZIP)/BT549-Fra1-ChIP-Seq(GSE46166)/Homer	1e-122	-2.815e+02	0.0000	2499.0	7.07%	1344.1	4.30%
5		Atf3(bZIP)/GBM-ATF3-ChIP-Seq(GSE33912)/Homer	1e-113	-2.603e+02	0.0000	2922.0	8.27%	1672.0	5.35%
6		Fra2(bZIP)/Striatum-Fra2-ChIP-Seq(GSE43429)/Homer	1e-111	-2.573e+02	0.0000	2078.0	5.88%	1086.7	3.48%
7		BATF(bZIP)/Th17-BATF-ChIP-Seq(GSE39756)/Homer	1e-100	-2.315e+02	0.0000	2838.0	8.03%	1657.4	5.30%
8		PU.1:IRF8(ETS:IRF)/pDC-Irf8-ChIP-Seq(GSE66899)/Homer	1e-97	-2.249e+02	0.0000	1261.0	3.57%	583.8	1.87%
9		Fos(bZIP)/TSC-Fos-ChIP-Seq(GSE110950)/Homer	1e-92	-2.141e+02	0.0000	2605.0	7.37%	1517.0	4.85%
10		JunB(bZIP)/DendriticCells-Junb-ChIP-Seq(GSE36099)/Homer	1e-92	-2.122e+02	0.0000	2424.0	6.86%	1390.1	4.45%
11		Fosl2(bZIP)/3T3L1-Fosl2-ChIP-Seq(GSE56872)/Homer	1e-87	-2.010e+02	0.0000	1440.0	4.07%	724.7	2.32%
12		IRF1(IRF)/PBMC-IRF1-ChIP-Seq(GSE43036)/Homer	1e-84	-1.955e+02	0.0000	1122.0	3.17%	523.1	1.67%

SCLC-SL

Total Target Sequences = 44971, Total Background Sequences = 44813

Rank	Motif	Name	P-value	log P-pvalue	q-value (Benjamini)	# Target Sequences with Motif	% of Targets Sequences with Motif	# Background Sequences with Motif	% of Background Sequences with Motif
1		Oct11(POU,Homeobox)/NCIH1048-POU2F3-ChIP-seq(GSE115123)/Homer	1e-1390	-3.202e+03	0.0000	6161.0	13.70%	1895.6	4.23%
2		Brn1(POU,Homeobox)/NPC-Brn1-ChIP-Seq(GSE35496)/Homer	1e-1115	-2.567e+03	0.0000	5340.0	11.87%	1723.5	3.85%
3		Oct2(POU,Homeobox)/Bcell-Oct2-ChIP-Seq(GSE21512)/Homer	1e-1071	-2.467e+03	0.0000	5540.0	12.32%	1884.6	4.21%
4		Oct4(POU,Homeobox)/mES-Oct4-ChIP-Seq(GSE11431)/Homer	1e-993	-2.288e+03	0.0000	6773.0	15.06%	2736.7	6.11%
5		Oct6(POU,Homeobox)/NPC-Pou3f1-ChIP-Seq(GSE35496)/Homer	1e-840	-1.936e+03	0.0000	5807.0	12.91%	2346.5	5.24%
6		Fra2(bZIP)/Striatum-Fra2-ChIP-Seq(GSE43429)/Homer	1e-231	-5.325e+02	0.0000	3211.0	7.14%	1726.0	3.85%
7		Fra1(bZIP)/BT549-Fra1-ChIP-Seq(GSE46166)/Homer	1e-228	-5.251e+02	0.0000	3725.0	8.28%	2117.0	4.73%
8		Fos(bZIP)/TSC-Fos-ChIP-Seq(GSE110950)/Homer	1e-226	-5.205e+02	0.0000	3937.0	8.75%	2283.0	5.10%
9		OCT4-SOX2-TCF-NANOG(POU,Homeobox,HMG)/mES-Oct4-ChIP-Seq(GSE11431)/Homer	1e-218	-5.028e+02	0.0000	2135.0	4.75%	996.9	2.23%
10		JunB(bZIP)/DendriticCells-Junb-ChIP-Seq(GSE36099)/Homer	1e-205	-4.736e+02	0.0000	3594.0	7.99%	2083.2	4.65%
11		Atf3(bZIP)/GBM-ATF3-ChIP-Seq(GSE33912)/Homer	1e-196	-4.522e+02	0.0000	4199.0	9.34%	2583.2	5.77%
12		Fosl2(bZIP)/3T3L1-Fosl2-ChIP-Seq(GSE56872)/Homer	1e-195	-4.491e+02	0.0000	2237.0	4.97%	1115.3	2.49%

SCLC-NE

Total Target Sequences = 3329, Total Background Sequences = 45606

Rank	Motif	Name	P-value	log P-pvalue	q-value (Benjamini)	# Target Sequences with Motif	% of Targets Sequences with Motif	# Background Sequences with Motif	% of Background Sequences with Motif
1		NeuroD1(bHLH)/Islet-NeuroD1-ChIP-Seq(GSE30298)/Homer	1e-117	-2.700e+02	0.0000	707.0	21.24%	3780.7	8.29%
2		Ascl1(bHLH)/NeuralTubes-Ascl1-ChIP-Seq(GSE55840)/Homer	1e-113	-2.618e+02	0.0000	1125.0	33.80%	7952.7	17.43%
3		Atoh1(bHLH)/Cerebellum-Atoh1-ChIP-Seq(GSE22111)/Homer	1e-100	-2.323e+02	0.0000	819.0	24.61%	5164.8	11.32%
4		DLX2(Homeobox)/BasalGanglia-Dlx2-ChIP-seq(GSE124936)/Homer	1e-94	-2.172e+02	0.0000	1081.0	32.48%	8045.8	17.64%
5		BHLHA15(bHLH)/NIH3T3-BHLHB8.HA-ChIP-Seq(GSE119782)/Homer	1e-90	-2.087e+02	0.0000	948.0	28.49%	6737.8	14.77%
6		Lhx2(Homeobox)/HFSC-Lhx2-ChIP-Seq(GSE48068)/Homer	1e-87	-2.026e+02	0.0000	826.0	24.82%	5559.0	12.19%
7		En1(Homeobox)/SUM149-EN1-ChIP-Seq(GSE120957)/Homer	1e-87	-2.021e+02	0.0000	1201.0	36.09%	9594.6	21.03%
8		MyoG(bHLH)/C2C12-MyoG-ChIP-Seq(GSE36024)/Homer	1e-85	-1.971e+02	0.0000	759.0	22.81%	4953.7	10.86%
9		NeuroG2(bHLH)/Fibroblast-NeuroG2-ChIP-Seq(GSE75910)/Homer	1e-85	-1.970e+02	0.0000	998.0	29.99%	7421.6	16.27%
10		Nkx6.1(Homeobox)/Islet-Nkx6.1-ChIP-Seq(GSE40975)/Homer	1e-85	-1.960e+02	0.0000	1532.0	46.03%	13619.2	29.86%
11		NF1-halfsite(CTF)/LNCaP-NF1-ChIP-Seq(Unpublished)/Homer	1e-84	-1.955e+02	0.0000	1078.0	32.39%	8314.3	18.23%
12		DLX5(Homeobox)/BasalGanglia-Dlx5-ChIP-seq(GSE124936)/Homer	1e-84	-1.954e+02	0.0000	674.0	20.25%	4151.4	9.10%
13		Twist2(bHLH)/Myoblast-Twist2.Ty1-ChIP-Seq(GSE127998)/Homer	1e-80	-1.856e+02	0.0000	1090.0	32.75%	8592.6	18.84%
14		LHX9(Homeobox)/Hct116-LHX9.V5-ChIP-Seq(GSE116822)/Homer	1e-78	-1.807e+02	0.0000	956.0	28.73%	7193.3	15.77%
15		DLX1(Homeobox)/BasalGanglia-Dlx1-ChIP-seq(GSE124936)/Homer	1e-78	-1.803e+02	0.0000	963.0	28.94%	7275.9	15.95%

B

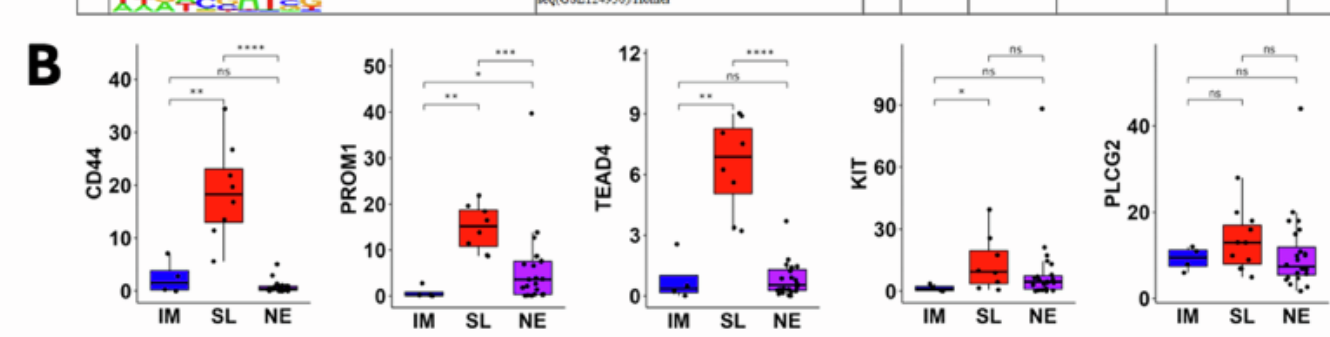

Figure S3. Enriched motifs and stem-associated features of chromatin-informed SCLC programs, related to Figure 3

A. HOMER-enriched transcription factor motifs for SCLC-IM, SCLC-SL, and SCLC-NE programs.
B. Boxplot showing promoter accessibility of canonical stem -associated marker genes. Center line indicates the median; box limits indicate the first and third quartiles; whiskers represent 1.5 × IQR; points represent individual samples. Tukey's multiple comparison test; * $p < 0.05$, ** $p < 0.005$, *** $p < 0.0001$

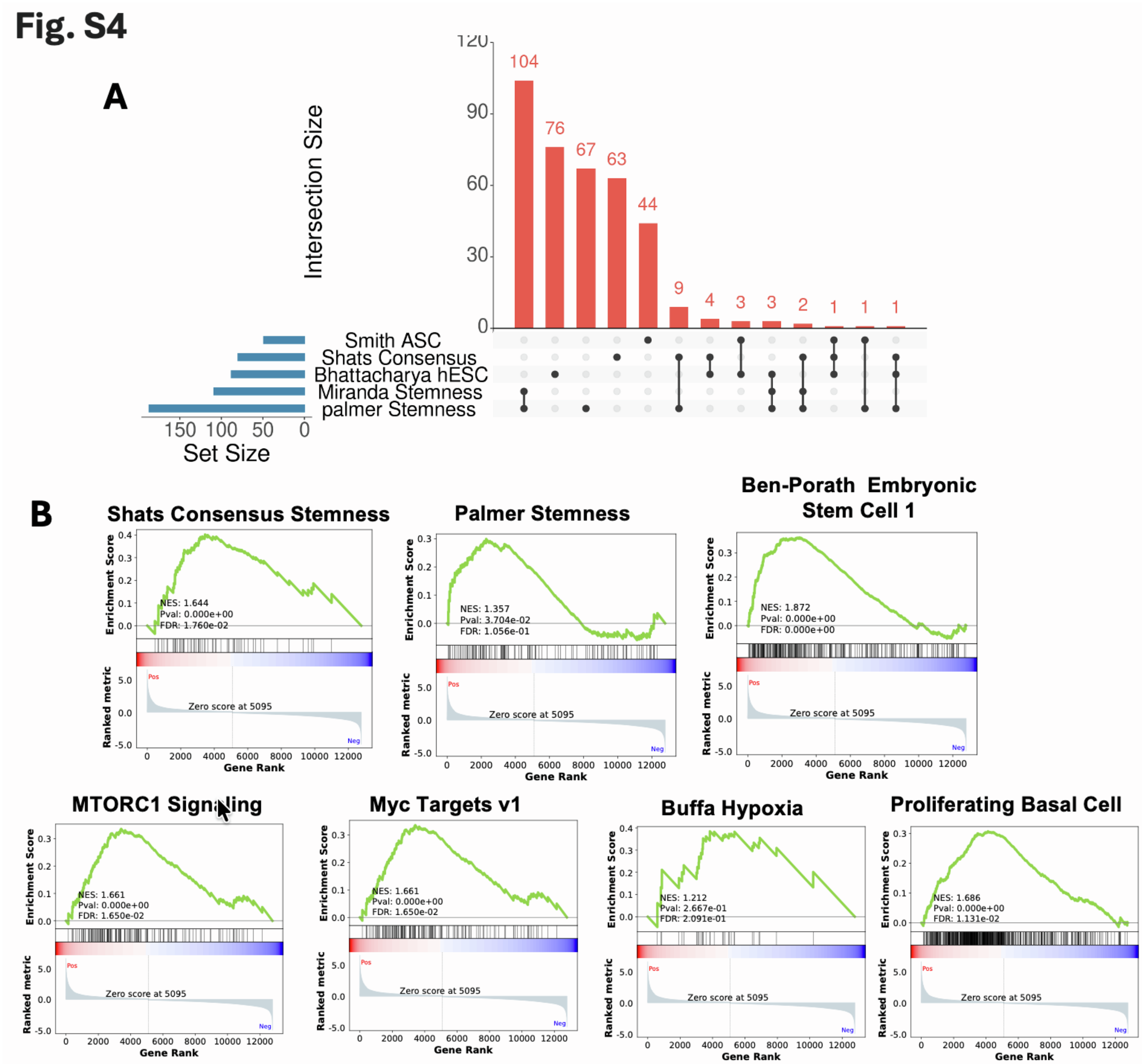

Figure S4. Stemness and metabolic features define the chromatin-informed SCLC-SL programs, related to Figure 4

A. UpSet plot depicting the degree of overlap among the stemness gene signature considered in the present study.
B. Gene set enrichment analysis shows multiple stemness signature along with metabolic signatures are exclusively high in SCLC-SL group compared to SCLC-IM and SCLC-NE.

Fig. S5

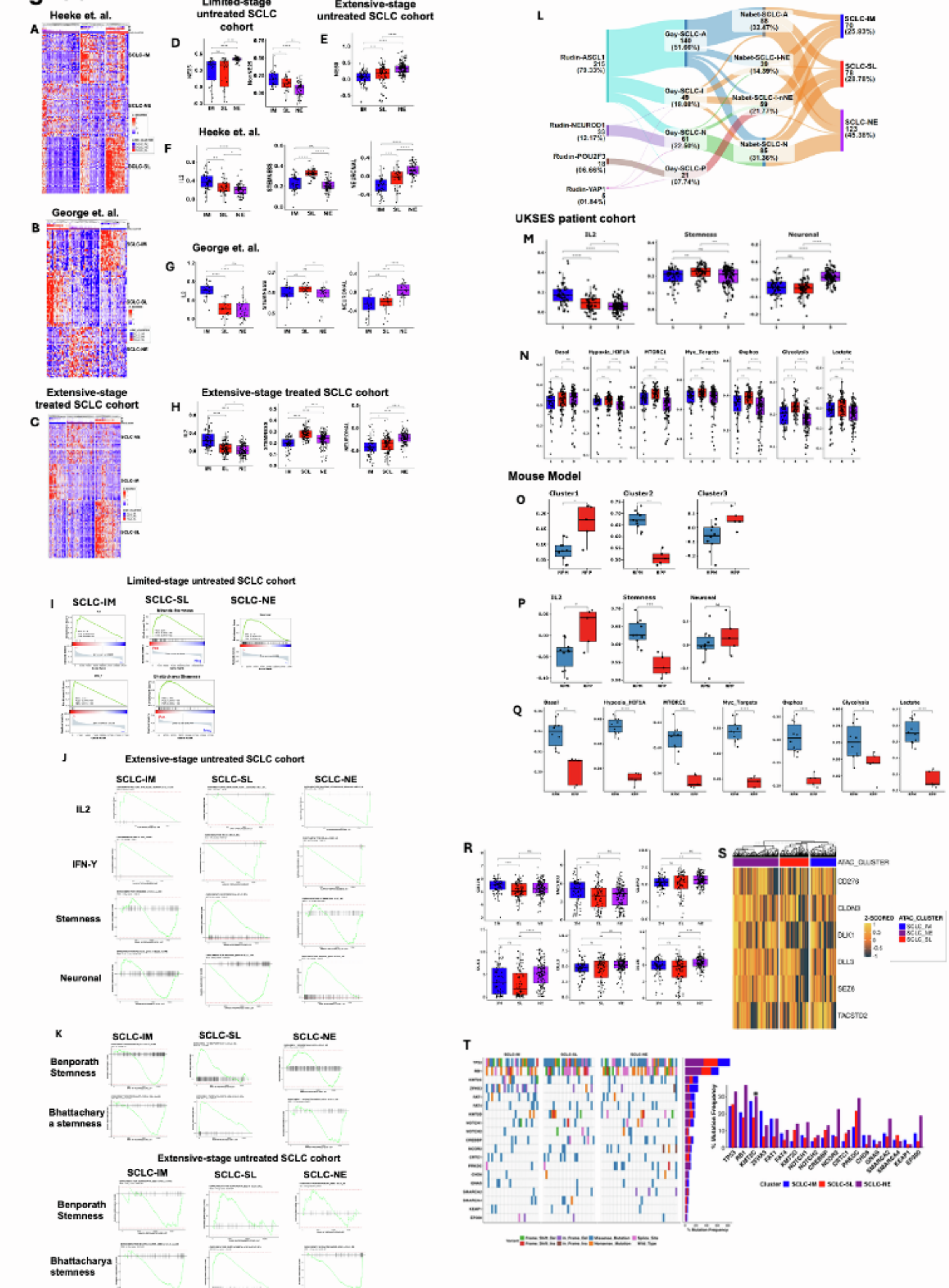
Heeke et. al.
A
SCLC-IM
SCLC-NE
SCLC-SL
George et. al.
B
Extensive-stage treated SCLC cohort
C
Limited-stage untreated SCLC cohort
D
Extensive-stage untreated SCLC cohort
E
Heeke et. al.
F
George et. al.
G
Extensive-stage treated SCLC cohort
H
Limited-stage untreated SCLC cohort
I
SCLC-IM
SCLC-SL
SCLC-NE
J
Extensive-stage untreated SCLC cohort
IL2
IFN-Y
Stemness
Neuronal
K
Benporath Stemness
Bhattachary a stemness
Extensive-stage untreated SCLC cohort
Benporath Stemness
Bhattacharya stemness
L
Rudin-ASCL1
Rudin-NEUROD1
Rudin-POU2F3
Rudin-YAP1
Gay-SCLC-A
Gay-SCLC-I
Gay-SCLC-N
Gay-SCLC-P
Nabet-SCLC-A
Nabet-SCLC-I-NE
Nabet-SCLC-i-nNE
Nabet-SCLC-N
SCLC-IM
SCLC-SL
SCLC-NE
UKSES patient cohort
M
IL2
Stemness
Neuronal
N
Basal
Hypoxia_HIF1A
MTORC1
Myc_Targets
Oxphos
Glycolysis
Lactate
Mouse Model
O
Cluster1
Cluster2
Cluster3
P
IL2
Stemness
Neuronal
Q
Basal
Hypoxia_HIF1A
MTORC1
Myc_Targets
Oxphos
Glycolysis
Lactate
R
S
ATAC_CLUSTER
CD276
CLDN3
DLK1
DLL3
SEZ6
TACSTD2
Z-SCORED
ATAC_CLUSTER
SCLC_IM
SCLC_NE
SCLC_SL
T
% Mutation Frequency
Cluster
SCLC-IM
SCLC-SL
SCLC-NE

Figure S5. Cross-cohort identification of chromatin-informed SCLC program and associated features, related to Figures 5

A-C. Heatmap showing relative expression of chromatin accessibility-defined program signature genes in Heeke et al.[51], George et al.[27], and NCI datasets. Top annotations indicate mean expression of signature genes derived from chromatin-based clusters

D, E. NE score in the chromatin-derived SCLC program identified in the limited stage[14] and extensive-stage [13]SCLC datasets. Center line indicates the median; box limits indicate the first and third quartiles; whiskers represent 1.5 × IQR; points represent individual samples. Statistical comparisons were performed using Tukey's multiple comparison test; * $p < 0.05$, **$p < 0.005$, ***$p < 0.0001$

F-H. Expression of IL2, stemness, and neuronal signatures in Heeke et al.[51], George et al.[27], and NCI Thomas datasets. Center line indicates the median; box limits indicate the first and third quartiles; whiskers represent 1.5 × IQR; points represent individual samples. Statistical comparisons were performed using Tukey's multiple comparison test; * $p < 0.05$, **$p < 0.005$, ***$p < 0.0001$

I.IL2, IFN-gamma, Stemness and neuronal pathways enrichment (GSEA-based) in SCLC-IM, SCLC-SL, and SCLC-NE in LS-SCLC tumors.

J. IL2, IFN-gamma, stemness and neuronal pathways enrichment (GSEA-based) in SCLC-IM, SCLC-SL, and SCLC-NE in ES-SCLC tumors.

K. Additional validation of stemness enrichment in SCLC-SL using multiple additional stem signatures[43-46] (GSEA-based) in chromatin accessibility defined SCLC tumors (LS-SCLC and ES-SCLC)

L. Sankey diagram depicting the reclassification of prior SCLC classifications (Rudin et al., Gay et al., Nabet et al) using the chromatin-based classification in extensive-stage SCLC patient tumors[13].

M. Boxplot showing pathway level activity of IL2, stemness, and neuronal signatures in UKSES cohort[57] (n=226). Center line indicates the median; box limits indicate the first and third quartiles; whiskers represent 1.5 × IQR; points represent individual samples. Statistical comparisons were performed using Tukey's multiple comparison test; * $p < 0.05$, **$p < 0.005$, ***$p < 0.0001$

N. Boxplot showing enrichment of unique biological programs in SCLC-SL (Cluster2) compared to the other, SCLC-IM (Cluster1) and SCLC-NE (Cluster3) in UKSES cohort. Center line indicates the median; box limits indicate the first and third quartiles; whiskers represent 1.5 × IQR; points represent individual samples. Statistical comparisons were performed using Tukey's multiple comparison test; * $p < 0.05$, **$p < 0.005$, ***$p < 0.0001$

O. Boxplots showing enrichment of chromatin-derived cluster signature in the SCLC mouse model transcriptomic dataset[58] (RPM and RPP). RPM mouse model shows enrichment of the Cluster2 (stem-like, SCLC-SL) signature, while significantly de-enriched for both Cluster1 and Cluster3 signature, indicating consistent with the SCLC-SL program. Center line indicates the median; box limits indicate the first and third quartiles; whiskers represent 1.5 × IQR; points represent individual samples. Statistical comparisons were performed using Tukey's multiple comparison test; * $p < 0.05$, **$p < 0.005$, ***$p < 0.0001$

P. Pathway level activity of the key functional programs in RPM and RPP mouse model. RPM model shows significant enrichment of stemness pathway while de-enriched for both IL2 and Neuronal programs. Center line indicates the median; box limits indicate the first and third quartiles; whiskers represent 1.5 × IQR; points represent individual samples. Statistical comparisons were performed using Tukey's multiple comparison test; * $p < 0.05$, **$p < 0.005$, ***$p < 0.0001$

Q. Additional pathway analysis demonstrating increased activity of basal cell, hypoxia (HIF1A), MTORC1 signaling, MYC targets, oxidative phosphorylation, glycolysis, and lactate-associated pathways in RPM tumors, consistent with a metabolically reprogrammed, stem-like phenotype. Center line indicates the median; box limits indicate the first and third quartiles; whiskers represent 1.5 × IQR; points represent individual samples. Statistical comparisons were performed using Student's t.test *$p < 0.05$, **$p < 0.01$, ***$p < 0.001$, ****$p < 0.0001$; ns, not significant

R. Expression levels of clinically relevant surface targets across chromatin accessibility informed programs in extensive stage disease[13]. Center line indicates the median; box limits indicate the first and third quartiles; whiskers represent 1.5 × IQR; points represent individual samples. Statistical comparisons were performed using Tukey's multiple comparison test; * $p < 0.05$, **$p < 0.005$, ***$p < 0.0001$

S. Comprehensive expression profiling of surface immune targets across chromatin-informed programs in extensive stage SCLC[13] following stromal deconvolution.

T. Mutation landscape and frequency bar plot showing significant enrichment of ZFHX3 mutation in SCLC-IM in LS-SCLC[14]. Statistical comparisons were performed using Fisher's exact test; * $p < 0.05$, **$p < 0.005$, ***$p < 0.0001$

GSEA, gene set enrichment analysis; NE, neuroendocrine; LS, Limited stage; ES, extensive stage

Fig. S6

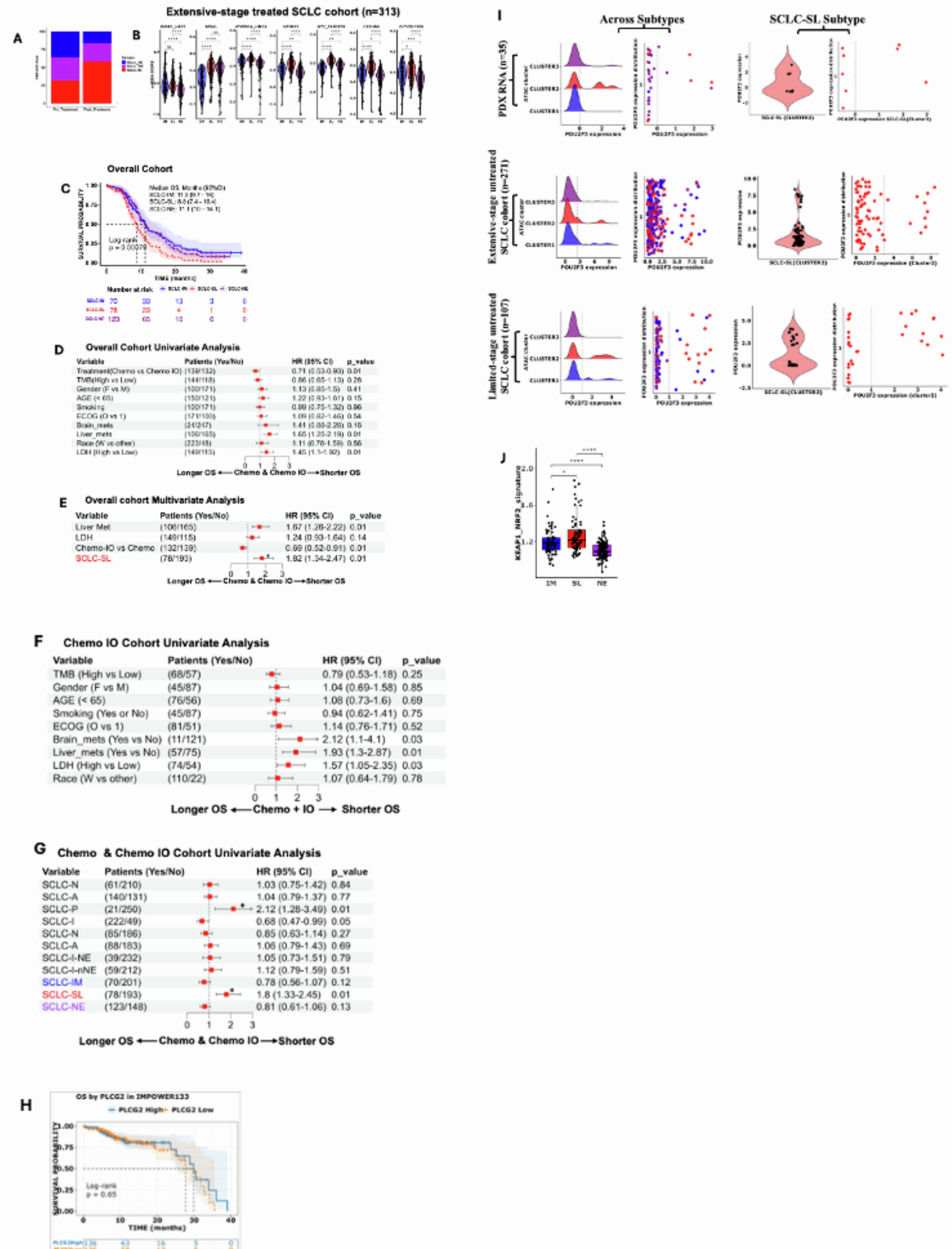
A
B
Extensive-stage treated SCLC cohort (n=313)
C
Overall Cohort
Log-rank
p = 0.00079
TIME (months)
Number at risk
D
Overall Cohort Univariate Analysis
Variable
Patients (Yes/No)
HR (95% CI)
p_value
Treatment(Chemo vs Chemo IO) (139/132) 0.71 (0.53-0.93) 0.01
TMB(High vs Low) (144/118) 0.86 (0.65-1.13) 0.28
Gender (F vs M) (100/171) 1.13 (0.85-1.5) 0.41
AGE (< 65) (150/121) 1.22 (0.93-1.61) 0.15
Smoking (100/171) 0.99 (0.75-1.32) 0.96
ECOG (O vs 1) (171/100) 1.09 (0.82-1.46) 0.54
Brain_mets (24/247) 1.41 (0.88-2.26) 0.16
Liver_mets (106/165) 1.65 (1.25-2.19) 0.01
Race (W vs other) (223/48) 1.11 (0.78-1.59) 0.56
LDH (High vs Low) (149/115) 1.45 (1.1-1.92) 0.01
Longer OS ⟵ Chemo & Chemo IO ⟶ Shorter OS
E
Overall cohort Multivariate Analysis
Variable
Patients (Yes/No)
HR (95% CI)
p_value
Liver Met (106/165) 1.67 (1.26-2.22) 0.01
LDH (149/115) 1.24 (0.93-1.64) 0.14
Chemo-IO vs Chemo (132/139) 0.69 (0.52-0.91) 0.01
SCLC-SL (78/193) 1.82 (1.34-2.47) 0.01
Longer OS ⟵ Chemo & Chemo IO ⟶ Shorter OS
F
Chemo IO Cohort Univariate Analysis
Variable
Patients (Yes/No)
HR (95% CI)
p_value
TMB (High vs Low) (68/57) 0.79 (0.53-1.18) 0.25
Gender (F vs M) (45/87) 1.04 (0.69-1.58) 0.85
AGE (< 65) (76/56) 1.08 (0.73-1.6) 0.69
Smoking (Yes or No) (45/87) 0.94 (0.62-1.41) 0.75
ECOG (O vs 1) (81/51) 1.14 (0.76-1.71) 0.52
Brain_mets (Yes vs No) (11/121) 2.12 (1.1-4.1) 0.03
Liver_mets (Yes vs No) (57/75) 1.93 (1.3-2.87) 0.01
LDH (High vs Low) (74/54) 1.57 (1.05-2.35) 0.03
Race (W vs other) (110/22) 1.07 (0.64-1.79) 0.78
Longer OS ⟵ Chemo + IO ⟶ Shorter OS
G
Chemo & Chemo IO Cohort Univariate Analysis
Variable
Patients (Yes/No)
HR (95% CI)
p_value
SCLC-N (61/210) 1.03 (0.75-1.42) 0.84
SCLC-A (140/131) 1.04 (0.79-1.37) 0.77
SCLC-P (21/250) 2.12 (1.28-3.49) 0.01
SCLC-I (222/49) 0.68 (0.47-0.99) 0.05
SCLC-N (85/186) 0.85 (0.63-1.14) 0.27
SCLC-A (88/183) 1.06 (0.79-1.43) 0.69
SCLC-I-NE (39/232) 1.05 (0.73-1.51) 0.79
SCLC-I-nNE (59/212) 1.12 (0.79-1.59) 0.51
SCLC-IM (70/201) 0.78 (0.56-1.07) 0.12
SCLC-SL (78/193) 1.8 (1.33-2.45) 0.01
SCLC-NE (123/148) 0.81 (0.61-1.06) 0.13
Longer OS ⟵ Chemo & Chemo IO ⟶ Shorter OS
H
OS by PLCG2 in IMPOWER133
PLCG2 High
PLCG2 Low
SURVIVAL PROBABILITY
Log-rank
p = 0.65
TIME (months)
I
Across Subtypes
SCLC-SL Subtype
PDX RNA (n=35)
Extensive-stage untreated SCLC cohort (n=271)
Limited-stage untreated SCLC cohort (n=107)
CLUSTER3
CLUSTER2
CLUSTER1
POU2F3 expression
J
KEAP1_NRF2_signature
IM
SL
NE

Figure S6. Prognostic and molecular features of chromatin-informed SCLC programs, related to Figure 6

A. Proportions of chromatin accessibility-informed programs for paired samples (NCI dataset) from the same patients across two time points along the treatment course.

B. Violin plot showing enrichment of unique biological programs in SCLC-SL, compared to the others (SCLC-IM and SCLC-NE) in NCI dataset. Violin plots show the distribution of ssGSEA scores; points represent individual patient tumors. Statistical comparisons were performed using Tukey's multiple comparison test. *$p < 0.05$, **$p < 0.005$, ***$p < 0.0001$.

C. Kaplan-Meier survival analysis of patients from the IMpower133 trial[26] with extensive-stage SCLC[13] treated with chemoimmunotherapy or chemotherapy (n = 271), stratified by the chromatin-based features.

D. Forest plot of univariate hazard ratios and clinical variables in overall cohort for ES-SCLC dataset[13] treated with chemoimmunotherapy or chemotherapy.

E. Multivariate cox regression analysis adjusting for liver metastases, LDH level, and treatment (chemoimmunotherapy or chemotherapy)

F. Forest plot of univariate hazard ratios and clinical variables in chemoimmunotherapy cohort for ES-SCLC dataset[13] (n=132 patients)

G. Forest plot of univariate hazard ratios for chromatin-informed programs and prior SCLC classifications from (Gay et al. and Nabet et al.) in overall cohort for ES-SCLC dataset[13] (n=271).

H. Kaplan-Meier overall survival analysis of Impower133 cohort (n = 271), stratified by PLCG2 expression.

I. POU2F3 expression across chromatin-defined SCLC program in PDX, limited-stage[14], and extensive-stage[13] SCLC cohorts. POU2F3 expression is heterogeneous within SCLC-SL and not exclusive to this subtype

J. KEAP1/NRF2 pathway signature in SCLC-SL subtype in the extensive-stage[13] SCLC cohort. Center line indicates the median; box limits indicate the first and third quartiles; whiskers represent 1.5 × IQR; points represent individual samples. Statistical comparisons were performed using Tukey’s multiple comparison test; * $p < 0.05$, **$p < 0.005$, ***$p < 0.0001$
