# Supplementary material for: A stem-like chromatin program in small-cell lung cancer is associated with poor outcomes after chemoimmunotherapy
Source: Cell Rep Med. 2026 Sep 10;7(10):103027. doi: 10.1016/j.xcrm.2026.103027 (PMC13710148; doi:10.1016/j.xcrm.2026.103027)
Supplement: Supplementary file 2 [file mmc11.pdf]

Cell Reports
Medicine

Article

A stem-like chromatin program in small-cell lung cancer is associated with poor outcomes after chemoimmunotherapy

Graphical abstract

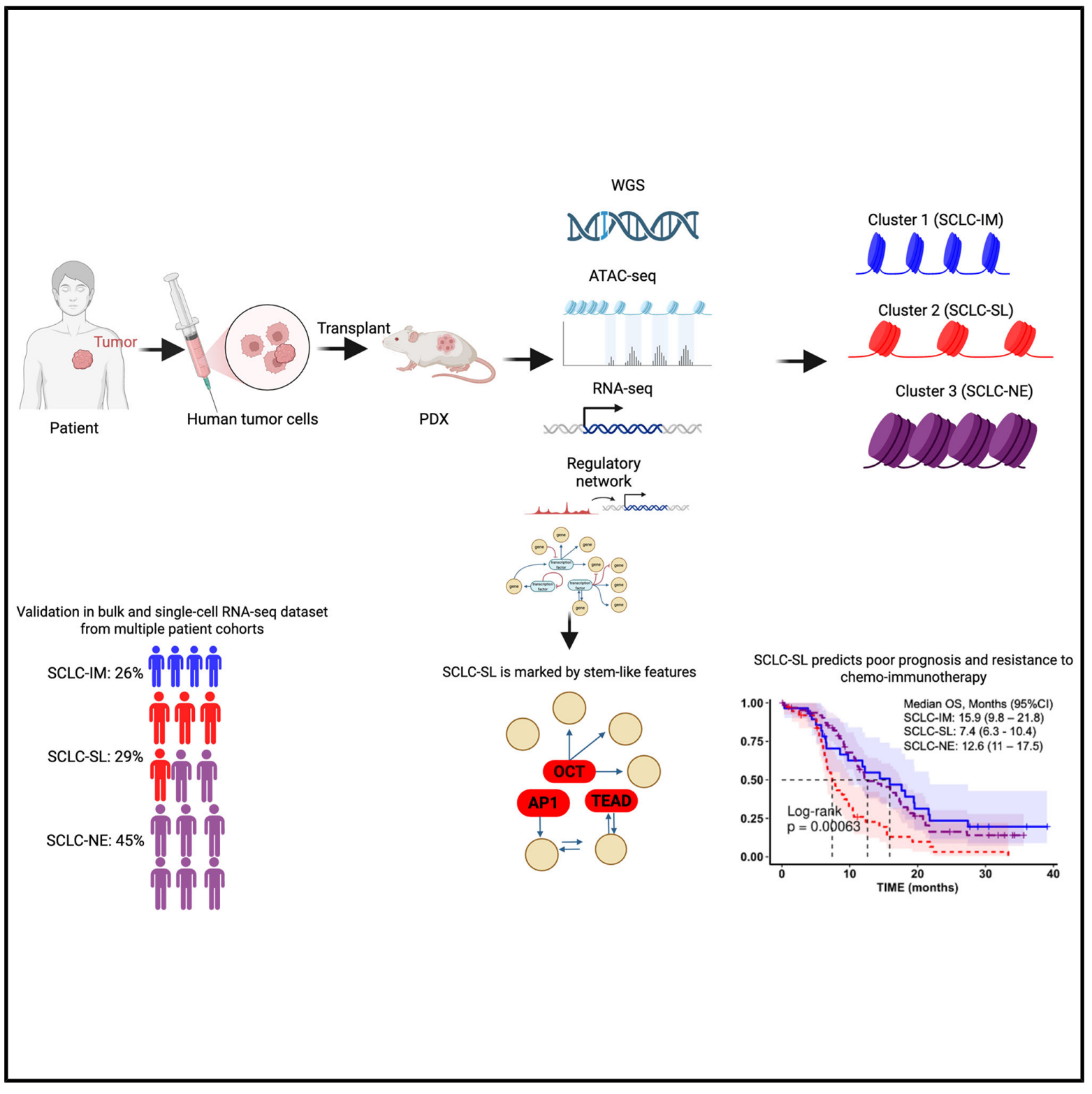

Authors

Rajesh Kumar, Parth Desai, Rajaa El Meskini, ..., Zoe Weaver Ohler, Lorinc Pongor, Anish Thomas

Correspondence

anish.thomas@northwestern.edu

In brief

Kumar et al. use ATAC-seq and transcriptomic analyses to define recurrent chromatin programs in small-cell lung cancer. A stem-like program is associated with metabolic adaptation, reduced immune signaling, and inferior outcomes after chemoimmunotherapy, supporting further development of chromatin-informed biomarkers and therapeutic strategies.

Highlights

- ATAC-seq identifies recurrent chromatin programs in SCLC PDX models
- SCLC-SL shows stem-like, metabolic, and immune-excluded features
- SCLC-SL is associated with inferior chemoimmunotherapy outcomes
- Chromatin-informed signatures are detected across SCLC cohorts

Kumar et al., 2026, Cell Reports Medicine 7, 103027
October 20, 2026 Published by Elsevier Inc.
https://doi.org/10.1016/j.xcrm.2026.103027

Cell Reports Medicine

CellPress
OPEN ACCESS

Article

A stem-like chromatin program in small-cell lung cancer is associated with poor outcomes after chemoimmunotherapy

Rajesh Kumar,[1,14] Parth Desai,[1,2,14] Rajaa El Meskini,[3,14] Chirayu Mohindroo,[1,13,15] Alexandra Ágnes Benő,[4,15] Devon Atkinson,[3,15] Michael Nirula,[1,15] Laura Bassel,[3,15] Nobuyuki Takahashi,[1,5,15] Kelly L. Sovacool,[6,7] Anna Meinhardt,[1] Mohit Arora,[1] Kanak Parmar,[1] Darryl Nausome,[6] Samantha Nichols,[1] Linda Sciuto,[1] Shannon Swift,[8] Kolos Nemes,[4] Ajit Kumar Sharma,[1] Nathan Pate,[3] Arun Rajan,[8] Elliot Levy,[9] Michael Kassin,[9] Christopher Febres Aldana,[10] Vishal Koparde,[6,7] Melissa Galloux,[11] Roshan Shreshta,[1] Zoe Weaver Ohler,[3] Lorinc Pongor,[4] and Anish Thomas[1,12,16,*]

[1]Developmental Therapeutics Branch, Center for Cancer Research, National Cancer Institute, National Institutes of Health, Bethesda, MD, USA

[2]Department of Medical Oncology, Fox Chase Cancer Center, Philadelphia, PA, USA

[3]Center for Advanced Preclinical Research, Frederick National Laboratory for Cancer Research, National Cancer Institute, National Institutes of Health, Frederick, MD, USA

[4]Cancer Genomics and Epigenetics Core Group, Hungarian Centre of Excellence for Molecular Medicine, Szeged, Hungary

[5]Department of Medical Oncology, National Cancer Center Hospital East, Kashiwa, Japan

[6]Collaborative Bioinformatics Resource, Center for Cancer Research, National Cancer Institute/National Institutes of Health, Bethesda, MD, USA

[7]Advanced Biomedical Computational Science, Frederick National Laboratory Research, Frederick, MD, USA

[8]Thoracic and GI Oncology Branch, Center for Cancer Research, National Cancer Institute, Bethesda, MD, USA

[9]Interventional Radiology, National Institutes of Health Clinical Center, Bethesda, MD, USA

[10]Laboratory of Pathology, National Institutes of Health Clinical Center, Bethesda, MD, USA

[11]Independent Bioinformatician, Marseille, France

[12]Robert H. Lurie Comprehensive Cancer Center and Division of Hematology and Oncology, Feinberg School of Medicine, Northwestern University, Chicago, IL, USA

[13]Division of Hematology, Oncology, and Blood & Marrow Transplantation, Department of Internal Medicine, University of Iowa Carver College of Medicine, Iowa City, IA, USA

[14]These authors contributed equally

[15]These authors contributed equally

[16]Lead contact

*Correspondence: anish.thomas@northwestern.edu

https://doi.org/10.1016/j.xcrm.2026.103027

SUMMARY

Small-cell lung cancer (SCLC) is an aggressive malignancy with substantial tumor heterogeneity and limited clinically actionable biomarkers beyond established features such as liver metastases. We profile tumor-intrinsic chromatin accessibility in a patient-derived xenograft biobank and identify three recurrent chromatin programs: neuroendocrine, marked by ASCL1/NEUROD1 activity; immunogenic, marked by IRF-associated activity; and stem-like, marked by TEAD/OCT activity. These programs are reproduced at the cohort level across bulk and single-cell transcriptomic datasets comprising more than 800 tumors, including 300 extensive-stage samples. In patients treated with chemoimmunotherapy, the stem-like program is associated with inferior survival, including a median overall survival of 7.41 months versus 15.9 and 12.6 months for immunogenic and neuroendocrine groups, respectively. This association remains significant after adjustment for liver metastases, brain metastases, and elevated lactate dehydrogenase. These findings support a high-risk stem-like SCLC chromatin program for prospective biomarker refinement and therapeutic investigation.

INTRODUCTION

Small-cell lung cancer (SCLC) is a highly aggressive neuroendocrine (NE) malignancy that accounts for approximately 15% of all lung cancer cases.[1] It is defined by rapid tumor growth, early dissemination,[2] and near-universal recurrence following an initial response to chemotherapy.[3] Despite decades of research, overall survival (OS) remains poor; less than 5% of patients with extensive-stage SCLC survive beyond five years. Clinically and molecularly, SCLC is highly heterogeneous,[4–8] yet current therapeutic decisions are not informed by molecular biomarkers capable of predicting treatment response or long-term outcomes.

Recent studies have proposed molecular subtypes of SCLC based on transcriptomic profiling, centered around the

This is an open access article under the CC BY license (http://creativecommons.org/licenses/by/4.0/).

expression of lineage-defining transcription factors (TFs), such as ASCL1 and NEUROD1 (which define NE subtypes), and POU2F3, and YAP1 (which define non-NE subtypes).[9–15] While these frameworks have improved our understanding of SCLC biology, their clinical utility has been limited.[9–14,16–21] One challenge is that most existing classifications rely on bulk tumor RNA sequencing (RNA-seq), which reflects a composite signal from tumor cells together with stromal and immune components. This can dilute tumor-intrinsic regulatory programs and complicate subtype assignment, particularly in advanced-stage or heavily treated tumors. Consistent with this, associations between transcriptomic subtypes and therapeutic response have varied across studies and prospective validation remains an active area of investigation.[9–14,16–21]

Compounding these challenges is the limited availability of high-quality tumor tissue in SCLC, owing to the aggressive nature of the disease and the reliance on small biopsies that are often low in tumor content.[22] This scarcity of tissue has impeded comprehensive molecular characterization, particularly in the relapsed setting where treatment resistance emerges. One approach to overcome these limitations is the use of patient-derived xenograft (PDX) models[23,24] which yield larger quantities of tumor material with minimal stromal contamination. These models enable more robust investigation of tumor-intrinsic biology and facilitate deeper molecular profiling. Chromatin profiling offers a compelling approach to directly access regulatory programs that drive cellular identity. Techniques such as ATAC-seq (assay for transposase-accessible chromatin using sequencing) provide a direct readout of chromatin accessibility, identifying active regulatory elements and TF-binding sites.[25] We hypothesized that chromatin accessibility could reveal tumor-intrinsic regulatory programs in SCLC that are obscured by bulk transcriptomic analyses. Applying ATAC-seq to a biobank of relapsed SCLC PDX models, we identify three recurrent chromatin accessibility programs: NE (SCLC-NE), immunogenic (SCLC-IM), and stem like (SCLC-SL). We then derive chromatin-informed gene expression signatures to evaluate these programs across bulk and single-cell RNA-seq (scRNA-seq) datasets from more than 800 tumors across disease stages and treatment contexts.

Among these programs, the SCLC-SL state is associated with poor outcomes in patients treated with chemoimmunotherapy, including in treatment-naive extensive-stage SCLC from the phase 3 IMpower133 trial.[13,26] This association remains significant after adjustment for established adverse clinical factors, including liver metastases, brain metastases, and elevated serum lactate dehydrogenase. Together, these findings support a chromatin-informed framework for studying recurrent SCLC regulatory states and nominate the stem-like program as a high-risk biology for prospective biomarker refinement and therapeutic investigation.

RESULTS

Establishment and characterization of SCLC-PDXs

We generated and characterized a biobank of PDX models from tumor biopsies of 20 patients with small-cell NE tumors. Most biopsies were obtained from liver (n = 11), with others from lymph nodes (n = 6), adrenal (n = 2), and breast (n = 1). All patients had received prior chemotherapy, 12 of 18 had received prior immunotherapy, and on average, had undergone two systemic lines of treatment before biopsy. The clinical and pathological characteristics of the cohort are summarized in Table S1. We focused on metastatic biopsies, as most patients with SCLC present with metastatic disease, and these samples may capture the biology of advanced, therapy-resistant tumors that drive poor clinical outcomes.

All tumors exhibited hallmark features of SCLC, including classic NE morphology on H&E-stained sections and, when evaluated, robust expression of diagnostic NE markers, such as synaptophysin (SYP) and INSM1, as well as the lung-lineage marker TTF-1 (Tables S1 and S2). Genomic profiling revealed frequent alterations in TP53 and RB1, as well as MYC amplification, consistent with known SCLC mutational landscapes.[27]

The derivative PDXs demonstrated robust engraftment, maintained growth kinetics reflective of clinical disease (Figure S1A), and recapitulated the NE histologic features of the original tumors. (Figure S1B). Immunohistochemical (IHC) analysis confirmed the preservation of NE marker SYP across models (Table S2).

Key driver alterations (TP53, RB1, and MYC-family amplifications) were preserved between patient tumors and their PDX derivatives (Table S1). Transcriptomically, PDX models showed more similarity to SCLC than adenocarcinomas of different tissues (Figure S1C), and clustered more closely with their corresponding patient tumors (Figure S1D).[28] After accounting for stromal contamination,[29] PDX transcriptomes were highly similar to the original tumors (Figure S1E). Moreover, protein expression of SCLC subtype-specific markers, particularly INSM1 was consistent across generations (Table S3; Figures S1F and S1G). One PDX model (TMSCLC20) exhibited a marked passage-dependent phenotypic shift by IHC, transitioning from ASCL1-high at P1 (H-score 190.15) to POU2F3-high at P3 (H-score 205.23) (Figure S1H). To determine whether passage-dependent IHC variability reflected broader transcriptional divergence, we performed bulk RNA-seq analyses across multiple passages (n = 35). These showed strong inter-passage correlations (Pearson $r > 0.8$–0.9; Figure S1I) and consistent clustering by PDX identity (Figure S1J), and stable SCLC lineage markers (Figure S1K), with the exception of TMSCLC16. These analyses indicate that although selective expansion of distinct subclones can occur during PDX establishment and passaging,[30] bulk transcriptional identity remains largely preserved. Together, these findings underscore the fidelity of the models and their utility for high-resolution molecular analyses.

Chromatin accessibility landscape identifies recurrent regulatory programs in SCLC

We performed ATAC-seq of 39 PDX tumors from 18 patients, including the first passage (P1) and a subsequent passage (P3). The majority of the ATAC-seq peaks mapped to promoter and distal intergenic regions (Figure 1A). Using non-negative matrix factorization (NMF), k = 3 was determined as the optimal number of clusters (Figure 1B; Figure S1L), with further sub-clustering yielding unstable groups (Figure S1M). To rigorously assess cluster robustness, we further evaluated NMF cluster stability across multiple metrics and perturbation analyses.

CellPress
OPEN ACCESS

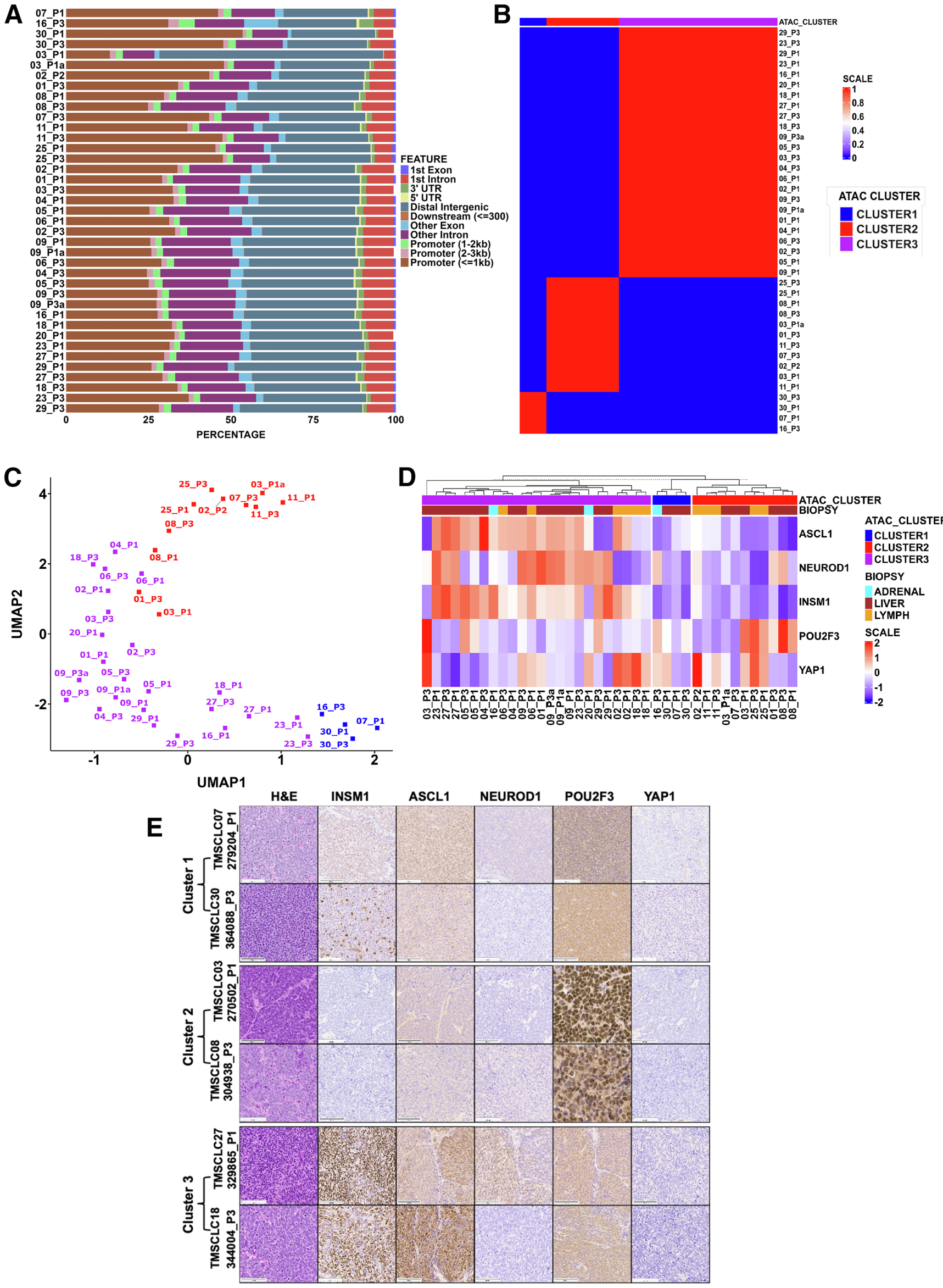

Figure 1. Chromatin accessibility profiling identifies recurrent regulatory programs in SCLC

(A) Feature distribution of the mapped ATAC-seq peaks across all samples, SCLC patient-derived xenografts (n = 39).

(B) Heatmap of NMF clustering based on the ATAC-seq peaks at k = 3 demonstrating best clustering resolution.

(C) Unsupervised UMAP on the top 1% most variable accessible peaks calculated using median absolute deviation across all samples (n = 39).

(D) Heatmap of chromatin accessibility of lineage-defining factors (ASCL1, NEUROD1, INSM1, POU2F3, and YAP1) across the three clusters of all samples (n = 39).

(E) Immunohistochemistry based protein expression of INSM1, ASCL1, NEUROD1, POU2F3 and YAP1 in representative PDX samples from each cluster. Tumors from passage 1–3 that represent the characteristics of each cluster are shown. Scale bars, 100 μm. ATAC, assay for transposase-accessible chromatin with sequencing; UMAP, uniform manifold approximation and projection; SCLC, small-cell lung cancer; PDX, patient-derived xenograft; NMF, non-negative matrix factorization; UTR, untranslated region

These analyses consistently identified k = 3 as the most stable clustering resolution, with higher ranks showing reduced stability and evidence of over-factorization (Table S4; Figures S1AA–S1CC). Furthermore, bootstrap-based perturbation analyses of k = 3 demonstrated that the three-cluster structure is reproducibly recovered across subsampling of both samples and features (Figure S1DD).

Dimensionality reduction (UMAP) using the top 1% most differentially accessible peaks revealed pronounced separation of samples into these three ATAC-seq-defined clusters (Figure 1C). Cluster assignments remained highly consistent when defined using the top 5% or 10% most variable peaks (Figure S1N), indicating robustness to peak selection thresholds.

In most cases, P1 and P3 tumors from the same parental tumor clustered together; however, five tumors (01, 02, 03, 07, and 16) exhibited divergent cluster distributions on ATAC-seq, consistent with evolutionary and regulatory state changes during passaging[30] rather than transcriptomic drift. Importantly, when models with divergent passage-level assignments are excluded, k = 3 remained the optimal solution, with higher ranks again showing reduced stability and fragmented consensus structure (Table S4; Figures S1EE and S1FF). Thus, the analysis supports a reproducible three-program structure in SCLC chromatin accessibility, while also indicating that individual tumors may contain or transition between regulatory states.

Gene ontology analysis of cluster-enriched accessible regions revealed distinct biological programs: cluster 3 was enriched for neuronal and ganglionic pathways, cluster 1 for antigen processing and interleukin signaling, and cluster 2 for stem-cell maintenance and proliferation with reduced neuronal and immune activity (Figure S1O).

To link chromatin accessibility to transcriptional output, we analyzed matched ATAC-seq and RNA-seq data from the same PDX samples (n = 35). Using ChIPseeker[31] with a ±3 kb window, accessible peaks were assigned to proximal genes. The top 1% most variable peaks showed a significant positive correlation with gene expression (R = 0.37, $p < 0.01$), whereas no meaningful correlation was observed when considering all peaks (R = 0.02, p = 0.9) (Figure S1P). Genes associated with cluster-enriched peaks were preferentially expressed in their corresponding chromatin-defined cluster (Figures S1Q and

S1R), supporting functional coupling between chromatin state and transcriptional programs.

Core SCLC markers (SYP and CDKN2A) showed high promoter accessibility across all clusters (Figure S1S). Cluster 3 exhibited increased accessibility at ASCL1 and NEUROD1, with enrichment of canonical NE lineage genes (ASCL1, NEUROD1, INSM1, CHGA, SYP, and NCAM1), defining it as the classical NE chromatin state (Figure 1D; Figures S1T–S1V).

In contrast, clusters 1 and 2 lacked coordinated hyper-accessibility of either NE or non-NE TF loci. Although a small subset of cluster 2 samples exhibited increased accessibility at the POU2F3 locus (Figure S1T), motif enrichment analysis showed that only a minority displayed high POU-family motif activity, while most had low or negative deviation scores. These findings indicate that POU2F3 does not define the dominant chromatin program of cluster 2 (Figure S1W).

IHC of lineage-specific TFs confirmed these distinctions: cluster 3 expressed NE lineage markers, while clusters 1 and 2 lacked a dominant TF, though some cluster 2 tumors expressed POU2F3 (Figure 1E; Table S3; Figure S1X).

Analysis of normal lung lineage programs revealed program-specific enrichment: cluster 2 was associated with proliferating basal cell signatures,[32] cluster 1 with AT2 programs,[32] and cluster 3 with NE lineage pathways[33] (Figure S1Y). Notably, chromatin-based classification remained robust even in tumors with mixed or heterogeneous ASCL1/NEUROD1/POU2F3/YAP1 expression programs (Figure S1Z), overcoming a key limitation of transcriptome-only TF-axis subtyping.

Together, these results support a three-state ATAC-seq framework that captures biologically distinct, lineage-associated regulatory architectures (classical NE, immune/AT2-like, and stem/proliferative). This framework highlights epigenetic heterogeneity and putative cell-of-origin features that are not fully resolved by transcriptome-based classification alone.

Transcriptomic profiles of the chromatin accessibility-defined SCLC programs

To further elucidate the molecular basis of the three chromatin-defined programs, we leveraged matched RNA-seq data from the same PDX tumors. This enabled direct comparison of transcriptional programs with chromatin accessibility states and allowed us to assess the biological distinctiveness of each cluster (Figure S2A).

Cluster 3 (SCLC-NE) exhibited strong enrichment for NE-associated pathways, including pancreatic beta cell, while immune-related pathways such as interferon gamma (IFN-γ) were significantly downregulated (Figure 2A, top part of the image), supporting the classification of cluster 3 as a canonical NE state. Cluster 2 (SCLC-SL) was characterized by enrichment of MYC targets, mTORC signaling, glycolysis, hypoxia, and peroxisome pathways (Figure 2A, middle part of the image), indicating a stem-like, metabolically active phenotype. Cluster 1 (SCLC-IM) demonstrated robust enrichment of immune activation programs, including interferon signaling, allograft rejection, and complement activation (Figure 2A, bottom part of the image), suggesting a distinctly immunogenic state with active antigen presentation and inflammatory signaling. Comparative analysis further highlighted the inverse enrichment patterns of immune programs between clusters 1 and 2.

To further probe functional distinctions, we assessed curated gene sets associated with neuronal differentiation, stemness, and antigen presentation (Figure 2B). Cluster 3 was selectively enriched for neuronal differentiation and nervous system development, consistent with its NE identity. Cluster 2 showed strong enrichment for adult stem cell and stem cell-related gene sets, while being negatively enriched for MHC class I/II antigen presentation, aligning with a stem-like, immune-excluded phenotype. In contrast, cluster 1 was enriched for MHC expression and complement activation, supporting its immunogenic profile.

Quantitative scoring of IL-2 signaling, stemness, and neuronal differentiation across clusters confirmed these trends (Figure 2C). IL-2 signaling was highest in cluster 1, stemness highest in cluster 2, and neuronal and NE scores were highest in cluster 3 (Figure S2B). Importantly, matched RNA-seq analyses of the PDX sample after removing discordant replicates, recapitulated the same pattern, with cluster 2 showing increased stemness and concomitant reduction in IL-2 and neuronal programs (Figure S2C). Notably, there was minimal gene-level overlap between stemness and epithelial-to-mesenchymal transition (EMT) (3 shared genes out of 109 stemness and 200 EMT genes), suggesting that stemness in SCLC-SL represents a distinct biological program rather than a surrogate for EMT.[34]

These findings delineate three transcriptionally and functionally distinct chromatin-informed SCLC programs: SCLC-IM (immune active), SCLC-SL (stem like and metabolically reprogrammed), and SCLC-NE (NE differentiated), as defined by integrated chromatin accessibility and transcriptomic profiling.

Transcriptional regulators of chromatin-based SCLC programs

Chromatin accessibility analysis can be informative of the transcriptional regulators driving the biological diversity of SCLC regulatory programs. Using HOMER-based motif analysis, we identified both shared and program-enriched TFs across the ATAC-seq-defined SCLC clusters (Table S5; Figure S3A) in the PDX dataset.

Top TF hits in SCLC-NE included NEUROD1[35] and ASCL1,[36] well-established drivers of the NE lineage (Figure 3A). Additional enriched TFs included ATOH1 and DLX2/DLX1, emphasizing the role of neuronal lineage-specific regulators. Unique TFs for SCLC-NE were predominantly members of the basic helix-loop-helix (bHLH) family, further supporting the NE characteristics of this chromatin-transcriptional state.[37] Both SCLC-IM and SCLC-SL exhibited significant enrichment for components of the AP1/TEAD pathway, including FRA1, FRA2, ATF3, JUNB, and FOS. These factors, part of the basic leucine zipper (bZIP) TF family, are known for their roles in cellular stress responses.[38,39] SCLC-IM uniquely demonstrated enrichment of interferon response factors (IRF), including IRF8, aligning with the observed increase in tumor-intrinsic immune signaling. SCLC-SL displayed a distinct profile enriched for POU Homeobox family TFs, such as OCT1, BRN1, OCT2, OCT4, and OCT6,[40] which are associated with stemness. Consistent with this stem-like program, several canonical stem-associated markers, including CD44, PROM1 (CD133), TEAD4, and KIT, were also enriched in SCLC-SL, supporting its

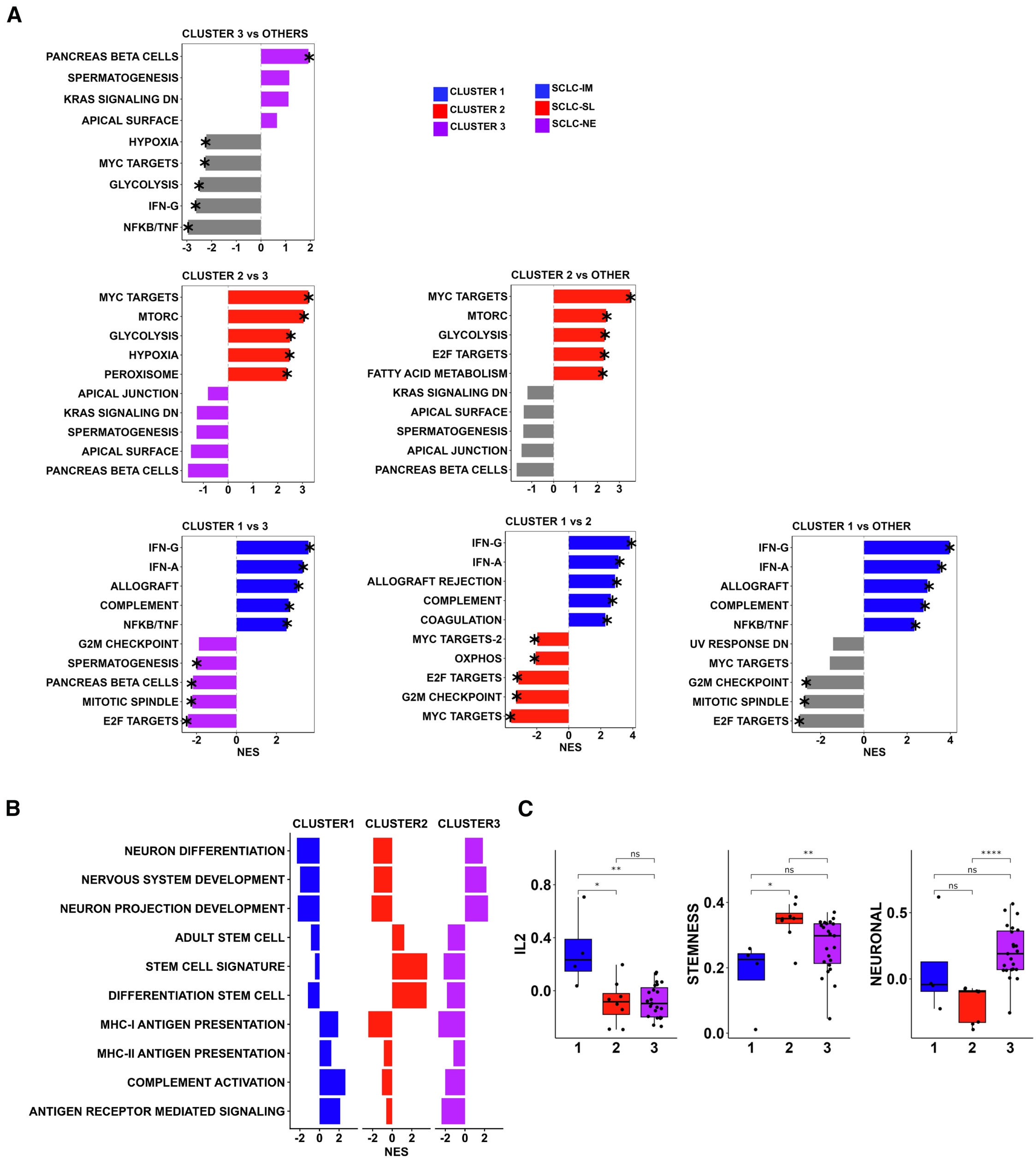

Figure 2. Transcriptomic features of chromatin-defined SCLC regulatory programs

(A) Gene set enrichment analysis of Hallmark pathways comparing each chromatin-defined cluster to the others (e.g., cluster 1 vs. others) and in pairwise comparisons (e.g., cluster 1 vs. cluster 2), using matched RNA-seq data from PDX models shown in Figure 1 (n = 35). Asterisks indicate $p < 0.05$ by Tukey's multiple comparison test.

(B) Key biological pathways enriched in each chromatin accessibility-defined cluster in matched PDX transcriptomes (n = 35).

(C) Pathway activity using single-sample gene set enrichment analysis, showing upregulation of IL-2 signaling in cluster 1, stemness signature in cluster 2 and neuronal signaling in cluster 3. Center line indicates the median; box limits indicate the first and third quartiles; whiskers represent 1.5 × IQR; points represent individual samples. Statistical comparisons were performed using Tukey's multiple comparison test; *$p < 0.05$, **$p < 0.005$, ***$p < 0.0001$ GSEA: gene set enrichment analysis; cluster 1 depicted in blue, cluster 2 in red; cluster 3 in purple, other clusters gray; NES, normalized enrichment scores; ssGSEA, single sample gene set enrichment analysis.

association with an undifferentiated regulatory state. In contrast, PLCG2, previously described as a stress-responsive, pro-metastatic population recurrent across multiple SCLC tumors,[8] was not uniquely enriched within SCLC-SL, consistent with its broad distribution across SCLC states rather than marking a fixed stem-like lineage (Figure S3B).

We constructed cluster-specific regulatory networks by integrating matched ATAC-seq and RNA-seq data to link chromatin

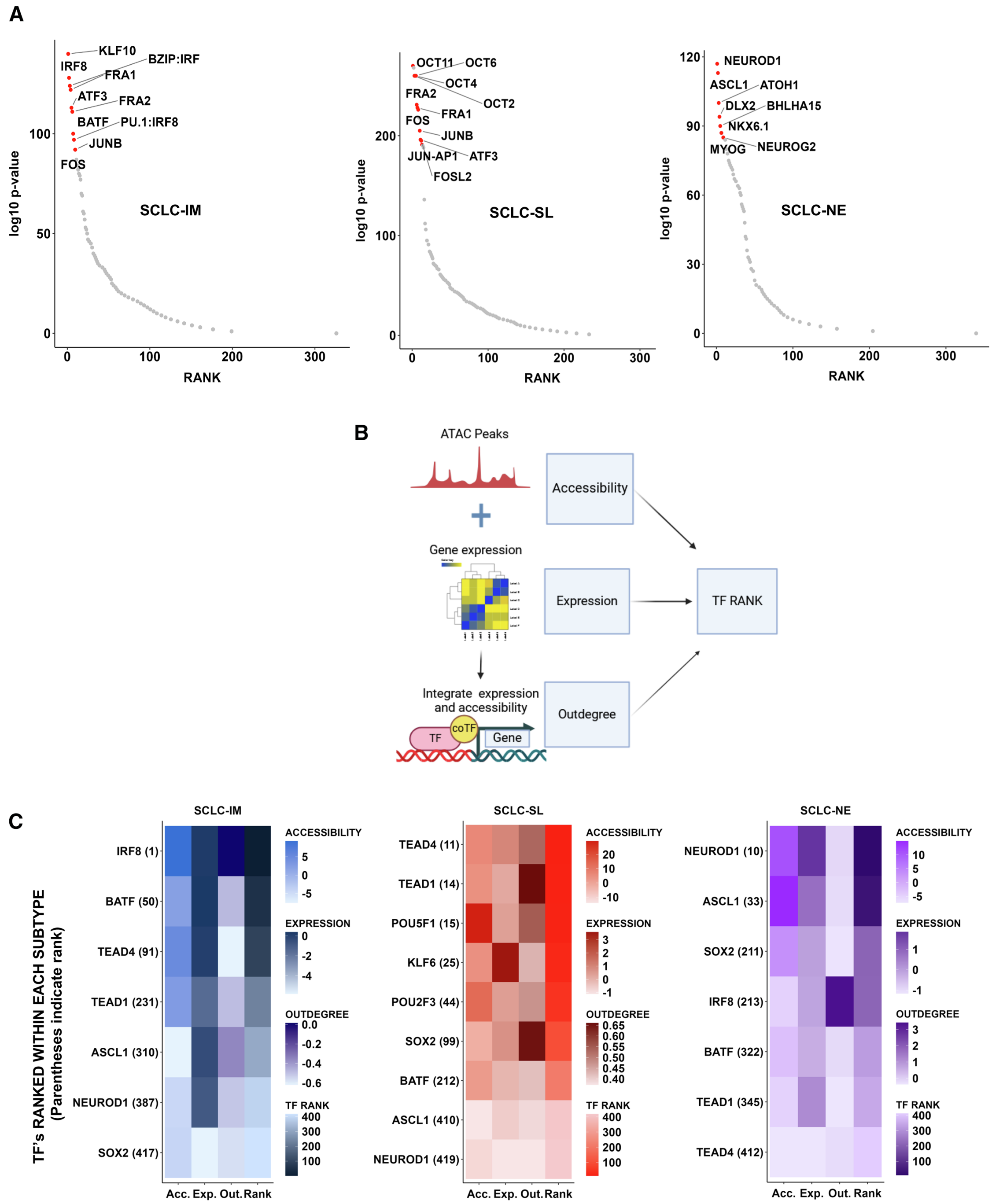

Figure 3. Candidate transcriptional regulators of chromatin-informed SCLC programs

(A) Candidate transcription factors associated with chromatin-defined SCLC programs. Differential chromatin accessibility analyzed using DESeq2 and cluster-specific peaks defined as significantly more accessible in one cluster compared to others ($log_2FC > 1$, $p < 0.05$). These peaks were then analyzed using the HOMER motif discovery algorithm to uncover transcription factors associated with each cluster. Data from SCLC PDXs (Figure 1).

(B) Schematic illustrates the workflow for constructing regulatory networks using chromatin accessibility data integrated with matched bulk transcriptomic data. Candidate TFs were prioritized within each cluster by combining chromatin accessibility and gene expression data. The "outdegree," derived from combined chromatin and expression metrics, was used to rank TFs in a cluster-specific manner. Data from SCLC PDXs (Figures 1 and 2).

(C) Heatmaps showing cluster-associated ranking of selected transcription factors from panel A, based on integrated analysis of chromatin accessibility and gene expression. For each TF, chromatin accessibility, expression, and regulatory outdegree, defined as the number of downstream target genes regulated within the inferred network, are displayed alongside the combined TF rank. TF rank (shown in parentheses) reflects the relative position within the global ranking of subtype-specific regulators.

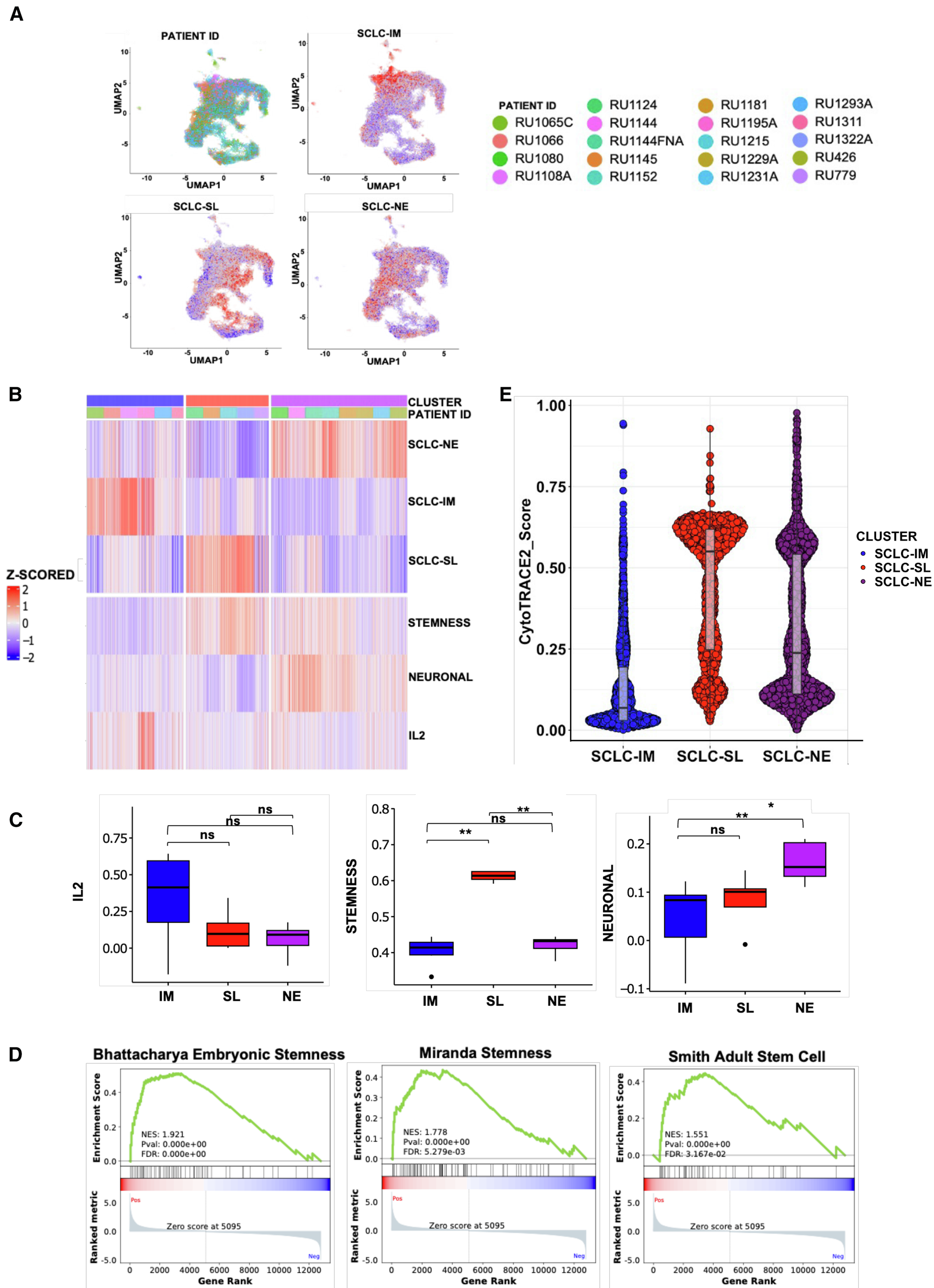

Figure 4. Single-cell transcriptomic analysis detects chromatin-informed SCLC programs in human tumors

(A) UMAP visualization of SCLC cells from 19 human SCLC tumors.[8] Cells are colored by chromatin-informed program signature score. Subtype scores were computed by projecting gene expression linked to the top 1% most variable ATAC-seq peaks (Figure 1C) onto the single-cell data.

(legend continued on next page)

accessibility with gene expression,[8] enabling the identification of peak-gene and TF-peak interactions (Figure 3B). Using this approach, we identified the key TFs for each cluster as those at the top of the inferred regulation hierarchy. Figure 3C shows the cluster-specific ranking of selected TFs, integrating chromatin accessibility, gene expression, and regulatory outdegree (the number of downstream targets regulated within each network). In SCLC-NE, NEUROD1 and ASCL1 were among the top two TFs, validating our approach (Figure 3C). In SCLC-IM, IRF8 and BATF, known for their roles in immune cell development,[41,42] were the highest-ranked. In SCLC-SL, TEAD 1 and 4 were identified as the top TFs, along with POU5F1 and POU2F3 reflecting their roles in maintaining stem-like properties. Thus, integrative analysis of matched tumor ATAC-seq and RNA-seq data identifies program-enriched candidate transcriptional regulators and supports the molecular distinction among the chromatin-informed SCLC regulatory states.

Single-cell transcriptomic validation of chromatin accessibility-based subtypes in patient tumors

Next, we analyzed the largest available scRNA-seq atlas of human SCLC[8] (19 samples, Figure 4A) by projecting gene expression signatures derived from the top 1% most variable ATAC-seq peaks (Figures 1 and 2) onto individual tumor cells. Chromatin-derived signature scores revealed that ATAC-defined subtype programs are conserved in human SCLC at the level of dominant cell states, although individual cells frequently exhibited mixed program activity, consistent with known SCLC plasticity.

Hierarchical clustering of single cancer cells using ATAC-seq-derived cluster signature scores classified all 19 patients into chromatin-based subtypes (Figure 4B): 8 samples as SCLC-NE, 5 as SCLC-SL, and 6 as SCLC-IM. These subtype programs were largely non-overlapping, indicating distinct regulatory states. Functionally, SCLC-NE cells exhibited significant activation of neuronal pathways, while SCLC-IM cells displayed increased IL-2 activity trends, suggesting enhanced tumor-intrinsic immunogenicity (Figure 4C).

The SCLC-SL subset was strongly enriched for stemness-associated programs, supported by multiple gene signatures derived from distinct biological contexts.[43–46] Although these gene sets showed limited overlap (Figure S4A), their collective enrichment in SCLC-SL underscores a robust and consistent stem-like phenotype (Figure 4D). In addition, SCLC-SL tumors exhibited enrichment for metabolic stress and growth programs, including MYC targets, mTORC1 signaling, and hypoxia, as well as transcriptional features associated with proliferating basal cells (Figure S4B), consistent with an undifferentiated, progenitor-like state.

To further explore this stem-like identity, we applied CytoTRACE, a computational framework based on the principle that transcriptional diversity, defined by the number of genes expressed per cell, tends to decrease as cells differentiate.[47] SCLC-SL cells occupied the least differentiated end of the trajectory, consistent with a stem-like phenotype (Figure 4E), while SCLC-NE and SCLC-IM cells exhibited lower potency scores, indicative of more differentiated states.

Together, these results demonstrate that chromatin-informed programs can be detected at single-cell resolution in human tumors and support the biological coherence of the chromatin-informed framework while highlighting regulatory heterogeneity across individual tumor cells.

Chromatin-informed regulatory programs are detected across independent cohorts and model systems

To evaluate the generalizability of our chromatin-based classification, we examined additional transcriptomics datasets.[13,14] Using the nearest template prediction (NTP) algorithm,[48] which assigns samples based on predefined gene signatures,[49,50] we projected chromatin-derived signatures onto independent bulk RNA-seq cohorts.

Using NTP, we reclassified transcriptomic data from both limited stage (n = 107)[14] and extensive stage (n = 271)[13] SCLC. In both disease stages, three recurrent programs corresponding to SCLC-IM, SCLC-SL, and SCLC-NE were detected (Figures 5A and 5B). We then extended this analysis to three additional cohorts totaling 537 tumors,[27,51] including an NCI cohort of extensive-stage SCLC obtained after multiple lines of therapy and relapse (n = 313). The NCI cohort represents a real-world extensive-stage SCLC dataset and includes tumors obtained after multiple lines of therapy, providing a clinically relevant complement to the treatment-naive extensive-stage[13] and early-stage cohorts.[27,51] Across all datasets, the chromatin-informed framework detected the same three recurrent programs observed in PDX and single-cell analyses (Figures S5A–S5C).

Consistent with patterns observed in PDXs, and single-cell datasets, SCLC-IM and SCLC-SL tumors displayed lower neuronal and NE scores compared to SCLC-NE-like tumors in both limited stage and extensive stage disease[13] (Figures 5C–5E; Figures S5D and S5E). Despite this shared suppression of NE features, the two groups displayed distinct biological programs: SCLC-IM tumors exhibited increased tumor-intrinsic immune signaling,

(B) Heatmap showing z-scored expression of chromatin-informed program signatures (SCLC-NE, SCLC-IM, and SCLC-SL) and selected functional programs (stemness, neuronal differentiation, and IL-2 signaling) across single tumor cells. For visualization, 100 representative cells per patient were selected. Data were integrated using Seurat with the SCTransform algorithm, and gene set enrichment scores were calculated using the AddModuleScore function. Top annotations indicate cluster designation and patient ID.

(C) Boxplots showing pathway activity scores across chromatin-informed programs using single-sample gene set enrichment analysis (ssGSEA): IL-2 signaling (left), stemness (middle), and neuronal signaling (right). Center line indicates the median; box limits indicate the first and third quartiles; whiskers represent 1.5 × IQR; points represent individual cells. Statistical comparisons were performed using Tukey's multiple comparison test; *$p < 0.05$, **$p < 0.005$, ***$p < 0.0001$; ns, not significant.

(D) Gene set enrichment analysis (GSEA) of stemness-associated signatures chromatin-informed programs. Cells enriched for the SCLC-SL program show increased stemness-associated activity compared with SCLC-NE and SCLC-IM.

(E) Developmental potential inferred using CytoTRACE. SCLC-SL (cluster 2) cells exhibit the highest potency scores, consistent with a less differentiated state, while SCLC-IM (cluster 1) and SCLC-NE (cluster 3) show lower scores, indicative of more differentiated phenotypes.

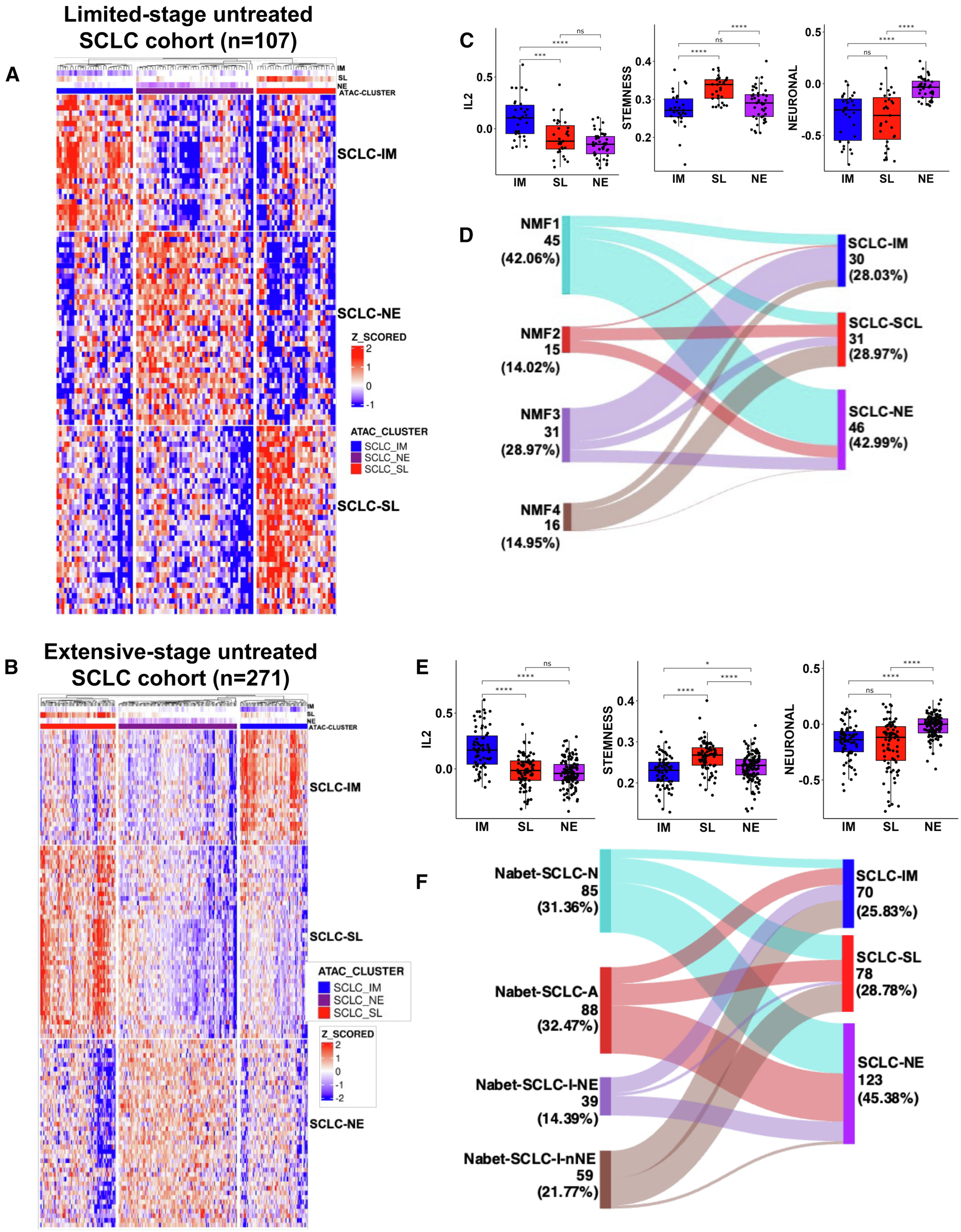

Figure 5. Chromatin-informed SCLC regulatory programs are detected across independent cohorts and model systems

(A) Heatmap showing relative expression of chromatin accessibility-derived program signature genes in limited-stage SCLC patient tumors (n = 107) from Liu et al.[14] Top annotations indicate mean expression of signature genes derived from chromatin-based clusters.

(B) Heatmap showing relative expression of chromatin accessibility-derived program signature genes in treatment-naive, extensive-stage SCLC patient tumors (n = 271) from the IMPOWER133 trial,[13,26] treated with atezolizumab or placebo and chemotherapy. Top annotations indicate mean expression of signature genes derived from chromatin-based clusters.

(legend continued on next page)

particularly through pathways, such as IL-2 and IFN-γ signaling[52] (Figures 5C–5E; Figures S5F–S5J). SCLC-SL tumors were characterized by enrichment of multiple stemness signatures, consistent with a progenitor-like phenotype (Figure S5K).

We next examined how chromatin-defined programs relate to previous molecular classifications. Using the Liu et al. integrative NMF framework,[14] reclassification revealed that Liu-NMF1 tumors (cell cycle and DNA damage) mapped predominantly to SCLC-NE; Liu-NMF3 tumors (extracellular matrix and receptor tyrosine kinase signaling) mapped mainly to SCLC-IM; and Liu-NMF4 tumors (MYC targets, chromosomal instability, and stemness) largely reclassified as SCLC-SL (Figure 5D). Liu-NMF2 tumors were distributed across SCLC-NE and SCLC-SL. Notably, SCLC-SL drew tumors from all Liu-NMF categories, indicating that it captures a unifying stem-like program spanning prior molecular groupings.

We further compared our framework with TF-based classification systems. Prior transcriptomic classifications of SCLC[9,10,12,53] were heavily weighted toward the ASCL1 subtype (80%), with only 2% categorized as YAP1, limiting their utility for effective patient stratification (Figure S5L). A recent refinement by Nabet et al. identified[13] NE inflamed (SCLC-I-NE) and non-NE inflamed (SCLC-I-nonNE) subsets. Applying the chromatin-based framework, most tumors previously assigned to SCLC-A or SCLC-N were reclassified as SCLC-NE, while SCLC-I-NE tumors mapped to either SCLC-NE or SCLC-IM. SCLC-I-nonNE tumors were reassigned to SCLC-IM or SCLC-SL (Figure 5F). Notably, tumors enriched for SCLC-SL were distributed across multiple prior categories, suggesting that this program captures a stem-like regulatory state not fully resolved by TF-based systems. Across extensive-stage cohorts, the chromatin-informed framework yielded a more balanced distribution, with approximately 45% of tumors enriched for SCLC-NE, 25% for SCLC-IM, and 29% for SCLC-SL.

To further evaluate the generalizability of the chromatin-derived framework, we performed validation in an additional patient cohort (UKSES; n = 226).[17] Consistent with prior cohorts, programs corresponding to SCLC-IM, SCLC-SL, and SCLC-NE were identified, recapitulating the chromatin-derived structure observed in PDX and earlier patient datasets. At the pathway level, tumors assigned to the SCLC-SL group demonstrated increased stemness-associated programs and relative depletion of IL-2/inflammatory and neuronal signatures (Figure S5M), while also showing enrichment of MYC-driven and metabolically reprogrammed pathways, including hypoxia (HIF1A), glycolysis, oxidative phosphorylation, lactate-associated, mTORC1, and proliferating basal cell programs (Figure S5N).

We next evaluated generalizability in an independent mouse SCLC dataset, comprising genetically engineered models with distinct oncogenic drivers, including RPM (Rb1/Trp53/Myc-driven) and RPP (Rb1/Trp53/Pten-driven) tumors. Projection of chromatin-derived signatures demonstrated that RPM tumors preferentially aligned with the stem-like (SCLC-SL) program, whereas RPP tumors showed relative depletion of this program (Figure S5O). This alignment is consistent with the known biology of MYC-driven SCLC,[54] including increased proliferative capacity, metabolic reprogramming, and reduced NE differentiation. At the pathway level, RPM tumors exhibited increased stemness-associated programs and reduced IL-2/inflammatory and neuronal signaling (Figure S5P), along with enrichment of hypoxia (HIF1A), MYC target, mTORC1 signaling, glycolysis, oxidative phosphorylation, lactate-associated, and proliferating basal cell pathways (Figure S5Q), further supporting their correspondence to the SCLC-SL phenotype.

Finally, to explore therapeutic opportunities, we assessed the expression of clinically actionable antibody-drug conjugate (ADC), bispecific T cell engager (BiTE), and chimeric antigen receptor T cell (CAR-T) surface antigens in bulk RNA-seq data of extensive stage SCLC corrected for stromal contribution.[29] SCLC-NE tumors expressed high levels of DLL3, DLK1, and SEZ6, which are associated with the ASCL1-driven NE program (Figures S5R and S5S), supporting sensitivity to tarlatamab and other DLL3-targeted ADCs. SCLC-IM tumors showed elevated expression of TROP2 (TACSTD2) and CD276, supporting the potential efficacy of agents, such as sacituzumab and govitecan, and CD276-directed therapies. In contrast, SCLC-SL tumors lacked strong enrichment for known ADC targets, highlighting the need to uncover therapeutic vulnerabilities in this stem-like, immune-excluded regulatory state.

Together, these findings support the recurrence and biological coherence of ATAC-derived SCLC regulatory programs across patient cohorts and model systems. The chromatin-informed framework provides a strategy for studying SCLC heterogeneity across disease stages using bulk RNA-seq data, while prospective studies with longitudinal and replicate sampling will be needed to determine the stability and clinical utility of individual sample-level assignments.

Mutation landscape across chromatin-defined SCLC programs

To determine genomic alterations associated with chromatin accessibility-informed SCLC programs, we analyzed paired whole-exome sequencing (WES) data from the Liu et al. dataset[14] (Figure S5T). Across all three programs, TP53 and RB1

(C) Expression of IL2, stemness, and neuronal signatures in limited-stage SCLC patient tumors.[14] Center line indicates the median; box limits indicate the first and third quartiles; whiskers represent 1.5 × IQR; points represent individual patient tumors. Statistical comparisons were performed using Tukey's multiple comparison test. *p < 0.05, **p < 0.005, ***p < 0.0001; ns, not significant.

(D) Sankey diagram depicting the reclassification of Liu et al.[14] NMF subtypes using the chromatin-informed SCLC programs in limited-stage SCLC patient tumors.

(E) Expression of IL2, stemness, and neuronal signatures in extensive-stage SCLC patient tumors.[13] Center line indicates the median; box limits indicate the first and third quartiles; whiskers represent 1.5 × IQR; points represent individual patient tumors. Statistical comparisons were performed using Tukey's multiple comparison test. *p < 0.05, **p < 0.005, ***p < 0.0001; ns, not significant.

(F) Sankey diagram depicting the reclassification of ES-SCLC patient tumors from the IMPOWER133 trial,[13,26] treated with atezolizumab or placebo and chemotherapy using the chromatin-informed SCLC programs. NTP, nearest template prediction.

CellPress
OPEN ACCESS

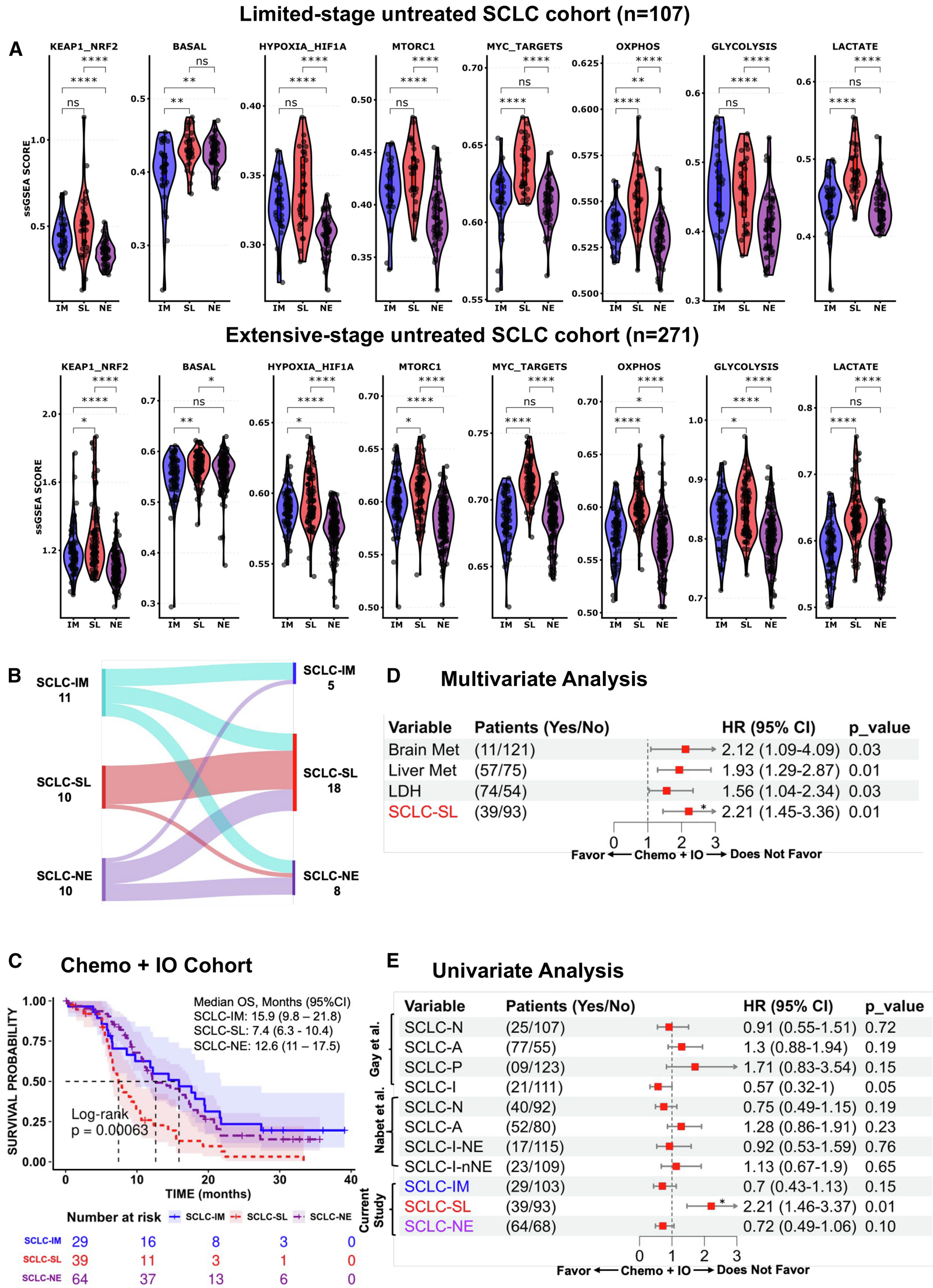

Figure 6. Clinical outcomes and biological features associated with the SCLC-SL program

(A) Violin plot showing enrichment of unique biological programs in SCLC-SL compared to the other subtypes (SCLC-IM and SCLC-NE) across the LS-SCLC[14] and ES-SCLC cohorts.[13] Violin plots show the distribution of ssGSEA scores; points represent individual patient tumors. Statistical comparisons were performed using Tukey's multiple comparison test. *$p < 0.05$, **$p < 0.005$, ***$p < 0.0001$.

(legend continued on next page)

were the most frequently mutated genes, consistent with their established roles as canonical drivers of SCLC.[55]

SCLC-IM tumors were enriched for mutations in the transcriptional regulator ZFHX3, consistent with prior observations that ZFHX3-mutant tumors exhibit elevated immune scores, increased IFN-γ and antigen presentation signaling, and enhanced responsiveness to immune checkpoint blockade.[13] Notably, ZFHX3 mutations have also been associated with increased tumor immunogenicity in other cancer types,[56] further supporting its role as a tumor-intrinsic modulator of immune activation.

In contrast, tumors enriched for the SCLC-NE program exhibited a higher frequency of mutations in PRKDC, which encodes the catalytic subunit of DNA-PK, a key mediator of nonhomologous end joining. Disruption of this DNA repair pathway may contribute to the heightened genomic instability characteristic of SCLC-NE.[11,57] Tumors enriched for the SCLC-SL program showed a trend toward increased mutation frequencies in CREBBP and CRTC1, although these did not reach statistical significance. CREBBP encodes a histone acetyltransferase that modulates chromatin accessibility and promotes transcription of genes involved in differentiation and tumor suppression[58]; its loss may reinforce a poorly differentiated, stem-like phenotype. CRTC1, a coactivator of CREB, regulates stress responses, metabolism, and survival. Together, the enrichment of CREBBP and CRTC1 mutations in SCLC-SL suggests coordinated disruption of epigenetic and transcriptional control, contributing to a stem-like, therapy-refractory state. Collectively, these findings suggest that chromatin chromatin-informed SCLC programs may be associated with distinct genomic alterations.

Metabolic and stress-response programs associated with SCLC-SL

To identify therapeutic vulnerabilities unique to the identified SCLC-SL program, we performed pathway enrichment analysis in both limited-stage (n = 107)[14] and extensive-stage (n = 271)[13] SCLC cohorts. Tumors enriched for SCLC-SL were significantly enriched for KEAP1-NRF2 signaling across both disease settings (Figure 6A), a pathway previously linked to chemotherapy resistance and poor outcomes in SCLC.[59]

In parallel, SCLC-SL-enriched tumors exhibited elevated MYC target activity and coordinated metabolic reprogramming, including enrichment of hypoxia/HIF1A, glycolysis, oxidative phosphorylation, and lactate and mTORC1 signaling, indicative of a metabolically adaptive and therapy-refractory state. SCLC-SL tumors were also associated with basal proliferative programs, consistent with emerging models in which non-canonical epithelial lineages contribute to cellular plasticity[60] (Figure 6A).

To determine how chromatin-informed programs evolve with therapy, we analyzed 31 paired pre- and post-treatment tumor samples from the NCI cohort (n = 313). At the initial time point, tumors were distributed as 35% SCLC-IM, 32.5% SCLC-SL, and 32.5% SCLC-NE (Figures 6B and S6A). At subsequent sampling, this distribution shifted markedly, with 58% classified as SCLC-SL, 26% as SCLC-NE, and 16% as SCLC-IM, indicating therapy-associated enrichment of the stem-like state. Consistent with this observation, the heavily treated extensive-stage NCI cohort also showed enrichment of KEAP1-NRF2, MYC-driven metabolic programs (hypoxia, glycolysis, OXPHOS, and mTORC1), and proliferative pathways specifically within SCLC-SL tumors (Figure S6B).

Collectively, these findings associate the SCLC-SL program with metabolic adaptation, stress-response signaling, and therapy-associated enrichment. They also generate testable hypotheses for targeting metabolic and stress-response pathways in tumors enriched for this stem-like regulatory program.

Clinical outcomes associated with the chromatin-informed framework

We evaluated the predictive and prognostic value of the ATAC-derived SCLC regulatory programs using RNA-seq from treatment-naive extensive-stage SCLC from patients enrolled on the phase 3 IMpower133 trial.[13,26] This trial evaluated the efficacy of adding atezolizumab or placebo to first-line treatment with carboplatin and etoposide. Based on the trial's results, the combination of atezolizumab, carboplatin, and etoposide is standard for first-line treatment of ES-SCLC.

In the overall cohort (n = 271) (Table S7), the key clinical and demographic features were well-balanced across treatment arms. Tumors enriched for the SCLC-SL program had significantly worse OS compared to tumors enriched for the SCLC-IM or SCLC-NE programs (8.8, 11.3, and 11.1 months, respectively; $p < 0.00079$; Figure S6C). To control for potential confounding variables, we performed univariate analyses, identifying immunotherapy treatment (hazard ratio [HR] 0.71, 95% confidence interval [CI] 0.53–0.93) to be associated with better OS, while liver metastasis (HR 1.65, 95% CI 1.25–2.19) and elevated serum lactate dehydrogenase levels (HR 1.45, 95% CI 1.10–1.92; Figure S6D) were associated with worse outcomes. After adjusting for confounding factors, enrichment of the SCLC-SL program remained associated with inferior OR (HR 1.82, 95% CI 1.34–2.47; Figure S6E).

Given that the current standard of care combines chemotherapy with immunotherapy, we further assessed the predictive value of the chromatin-based framework within the chemo-immunotherapy cohort (n = 132). Tumors enriched for the SCLC-SL program demonstrated significantly worse

(B) Changes in chromatin-informed program enrichment in paired samples (n = 31) of NCI dataset (n = 313) from the same patients across two time points along the treatment course.

(C) Kaplan-Meier survival analysis of patients with extensive-stage SCLC treated with chemoimmunotherapy (n = 132), stratified by chromatin-informed program enrichment.

(D) Multivariate analysis showing that enrichment of the SCLC-SL program is an independent prognostic indicator of poor survival in the chemoimmunotherapy cohort adjusting for brain metastases, liver metastases, and LDH level.

(E) Forest plot of univariate hazard ratios for chromatin-informed programs and previously defined SCLC classification systems from Gay et al. and Nabet et al. HR, hazard ratio; chemo-IO, chemoimmunotherapy; CI, confidence interval; *statistically significant, LS, limited stage; ES, extensive stage.

outcomes compared to tumors enriched for SCLC-IM or SCLC-NE (7.41 months vs. 15.9 months vs. 12.6 months, respectively; $p < 0.00063$ Figure 6C). To control for potential confounding variables, we performed univariate analyses, identifying brain metastases (HR 2.12, 95% CI 1.1–4.1), liver metastases (HR 1.93, 95% CI 1.3–2.87), and elevated LDH (HR 1.57, 95% CI 1.05–2.35; Figure S6F) to be predictive of worse outcomes. After adjusting for confounding factors, multivariate analysis showed enrichment of the SCLC-SL program was an independent predictor of worse OS (HR 2.21, 95% CI 1.45–3.36; Figure 6D) following chemo-immunotherapy.

Lastly, we compared our ATAC-based classification with existing transcriptomic classifications. Notably, these earlier classifications (Figures 6E and S6G) did not emerge as significant predictors in univariate analyses. SCLC-SL was associated with inferior chemotherapy outcomes (HR = 2.21, $p < 0.01$) after adjustment for clinical covariates, highlighting its potential as an important biomarker for SCLC.

A previous study identified a rare PLCG2^{+} stress-activated subpopulation associated with metastatic potential and poor outcomes in a small cohort[8] ($n = 27$). In the IMpower133 dataset ($n = 271$), PLCG2 expression was not predictive of OS ($p = 0.65$; Figure S6H), underscoring the distinction between a stress-responsive subpopulation and the stable chromatin-defined SCLC-SL state.

Together, these results support SCLC-SL as a therapy-enriched, chromatin-defined state with independent prognostic and predictive significance. These findings link metabolic stress-associated programs with reduced benefit from standard chemoimmunotherapy and provide a framework for further evaluating state-informed therapeutic strategies.

DISCUSSION

SCLC is the most aggressive type of lung cancer, characterized by rapid progression, early metastasis, and poor survival. It exhibits pronounced inter-tumoral heterogeneity, complicating efforts to predict clinical outcomes and guide treatment. Although transcriptomic subtyping has advanced the field, existing classifications have shown limited predictive value beyond established clinicopathological factors (e.g., liver or brain metastases, elevated serum LDH).[9,10,13,14] A key limitation is the reliance on bulk tumor transcriptomes (i.e., signatures derived from RNA-seq of patient tumors), which averages signals from across tumor cells, stroma, and immune infiltrates, obscuring critical tumor-intrinsic features.

Here, we use ATAC-seq from PDXs, integrated with matched transcriptomic data, to define three recurrent tumor-intrinsic chromatin accessibility programs in SCLC: NE, immunogenic, and stem like. The three-program structure is stable across multiple clustering metrics and perturbation analyses, but passage-level differences in some PDX models indicate that these programs should not be viewed as immutable patient classes. Rather, they represent recurrent regulatory states that can vary in dominance across samples, consistent with the known plasticity of SCLC.

Using chromatin-informed gene expression signatures, we detect these programs across independent bulk and single-cell datasets spanning limited-stage, extensive-stage, relapsed tumors, and in SCLC model-systems. These external datasets support the recurrence and biological coherence of the programs at the cohort level, although most do not contain biologic replicates and therefore cannot directly assess within-patient reproducibility of single-sample assignment. Clinically, tumors enriched for the stem-like program show inferior outcomes in patients treated with chemoimmunotherapy, including in the IMpower133 cohort, where this association remains significant after adjustment for liver metastases, brain metastases, and elevated LDH. These findings suggest that the stem-like program captures a high-risk, therapy-refractory biology and should be prioritized for prospective biomarker development and mechanistic therapeutic studies.

Although SCLC-SL program shares features with the previously described POU2F3 (SCLC-P) subtype (Figures 1D, 1E, 2A, and 3A), our data show that POU2F3 does not define the SCLC-SL regulatory state. POU2F3 expression was not limited to SCLC-SL tumors but was also observed in other chromatin-informed programs (Figure S1W), and only a subset of SCLC-SL tumors exhibited high POU2F3 levels (Figure S6I). Whereas SCLC-P represents a rare, transcriptionally defined tuft-cell lineage comprising <8% of SCLC tumors, SCLC-SL is substantially more prevalent (∼25%–30%) (Figure S5L) and encompasses both POU2F3-positive and POU2F3-negative tumors (Figure S6I). These findings indicate that POU2F3-driven regulation constitutes a context dependent within a broader stem-like regulatory state. By focusing on chromatin-informed regulatory programs rather than marker-defined lineages alone, this framework captures stem-like features of SCLC that may relate to tumor plasticity and clinical behavior.

The aggressive clinical behavior of the SCLC-SL program is supported by its underlying regulatory programs. Tumors enriched for SCLC-SL exhibit coordinated activation of pathways linked to therapy resistance and cellular adaptation, including MYC-driven transcription, mTORC1 signaling, hypoxia and HIF1A responses, and glycolytic and oxidative metabolic programs (Figure 6A; Figures S6B and S4B). In parallel, the SCLC-SL program is associated with reduced immune signaling and decreased NE lineage features, consistent with a more plastic and less differentiated regulatory state. Elevated KEAP1-NRF2 signaling may further contribute to oxidative stress tolerance and chemoresistance[59–61] (Figure 6A; Figures S6B and S6J). Together, these features support a stem-like, metabolically adaptable, and immune-evasive regulatory program that may contribute to the inferior outcomes observed in SCLC-SL-enriched tumors.

Limitations of the study

Several limitations should be acknowledged. First, the discovery cohort includes a relatively limited number of PDX models established predominantly from metastatic tumors. This reflects our intentional focus on advanced, treatment-resistant SCLC biology and therefore findings may not fully capture the regulatory program specific to untreated samples, but additional primary, treatment-naive, and longitudinal samples will be needed to further define the breadth of these regulatory programs. Second, although PDX models provide tumor-enriched material for

chromatin profiling with reduced stromal contamination, they do not fully preserve the native spatial tumor microenvironment. Therefore, the contributions of stromal, endothelial, and immune compartments to these chromatin-informed programs remain incompletely resolved. Third, external cohort analyses rely on projection of chromatin-derived gene expression signatures onto bulk or single-cell transcriptomic datasets. This approach supports recurrence of the programs across large patient cohorts but does not directly measure chromatin accessibility in those tumors. Although the chromatin-informed programs were reproducibly identified across clustering stability metrics and independent cohorts, a subset of PDX models exhibited discordant cluster assignments across passages, suggesting evolutionary selection during serial passage.[30] Moreover, because most validation cohorts include one profiled specimen per patient, they cannot directly assess within-patient reproducibility of single-sample assignment or dynamic transitions between states. Therefore, the current framework is best interpreted as identifying recurrent regulatory programs at the cohort level rather than establishing fixed subtype assignments for individual patients. Finally, associations linking the stem-like program to KEAP1-NRF2 signaling, metabolic adaptation, and treatment resistance are correlative and do not establish causal mechanisms. Future studies incorporating longitudinal sampling, single-cell chromatin profiling, spatial transcriptomics, and functional perturbation will be needed to define subtype-specific dependencies and evaluate the clinical utility of chromatin-informed patient stratification.

RESOURCE AVAILABILITY

Lead contact

Further information and requests for resources and reagents should be directed to and will be fulfilled by the lead contact, Anish Thomas, anish.thomas@northwestern.edu.

Materials availability

This study did not generate new unique reagents.

Data and code availability

- Data: The raw ATAC-seq data generated in this study have been deposited in dbGaP and are available under dbGaP: phs003983.v1.p1. The scRNA-seq and bulk RNA-seq datasets used for validation in Figures 4, 5, and 6 are available through dbGaP: phs002371, Genome Sequence Archive: HRA003419, and European Genome-Phenome Archive: EGAD50000000195, respectively. Additionally, patient clinical information, pathological characteristics, chromatin-derived gene signatures, and demographic and clinical characteristics of the validation cohorts are provided in Tables S1, S2, S6, and S7, respectively.
- Code: this paper does not report any original code.
- General statement: any additional information required to reanalyze the data reported in this work paper is available from the lead contact upon request.

ACKNOWLEDGMENTS

We gratefully acknowledge the contributions of our patients. This work utilized the computational resources of the NIH HPC Biowulf cluster (http://hpc.nih.gov). This work was supported by the intramural programs of the Center for Cancer Research, National Cancer Institute (ZIA BC 011793). We thank members of the Thomas lab and Developmental Therapeutics Branch for their insightful feedback.

AUTHOR CONTRIBUTIONS

Conceptualization, R.K., P.D., and A.T.; data curation, R.K., P.D., and R.E.M.; formal analysis, R.K., P.D., and A.T.; methodology, R.K., P.D., R.E.M., and A.T.; investigation, R.K., P.D., R.E.M., L.B., A.A.B., C.M., N.T., K.L.S., V.K., L.P., D.A., M.N., A.M., M.A., K.P., D.N., S.N., L.S., S.S., N.K., A.K.S., N.P., A.R., E.L., M.K., C.F.A., M.G., R.S., Z.W.O., and A.T.; visualization, R.K., P.D., C.M., and A.T.; writing – original draft, R.K., P.D., C.M., L.B., R.E.M., and A.T.; Writing – review and editing, R.K., P.D., C.M., and A.T.

DECLARATION OF INTERESTS

This study was supported by the Center for Cancer Research, the Intramural Program of the NCI (ZIA 011793 BC). A.T. received grants to NCI from EMD Serono Research & Development, AstraZeneca, Gilead Sciences, and ProLynx during the conduct of the study.

STAR★METHODS

Detailed methods are provided in the online version of this paper and include the following:

- KEY RESOURCES TABLE
- EXPERIMENTAL MODEL AND STUDY PARTICIPANT DETAILS
  - Human samples
  - Animal studies
- METHOD DETAILS
  - Human samples for PDX model development
  - PDX generation
  - Immunohistochemistry
  - ATAC-seq
  - RNA-seq
  - Whole exome data and somatic mutation analysis
  - Identification of features from ATAC-seq and determination of optimal clusters
  - Differential chromatin accessibility and cluster-specific features
  - Differential gene expression and pathway enrichment analysis
  - Analysis of single-cell RNA-seq data
  - Motif enrichment
  - Construction of gene regulatory networks
  - Subtype assignment of SCLC datasets
  - Extensive stage immunotherapy cohort
  - Other datasets
  - Survival analysis
- QUANTIFICATION AND STATISTICAL ANALYSIS

SUPPLEMENTAL INFORMATION

Supplemental information can be found online at https://doi.org/10.1016/j.xcrm.2026.103027.

Received: July 3, 2025
Revised: February 9, 2026
Accepted: August 12, 2026
Published: September 10, 2026

REFERENCES

1. Cancer IAfRo. Absolute numbers, Mortality, Both sexes, in 2022. 2022; https://gco.iarc.fr/today/en/dataviz/pie?mode=cancer&group_populations=1&types=1. Accessed May 6, 2024.
2. Thomas, A., Pattanayak, P., Szabo, E., and Pinsky, P. (2018). Characteristics and Outcomes of Small Cell Lung Cancer Detected by CT Screening. Chest *154*, 1284–1290. https://doi.org/10.1016/j.chest.2018.07.029.
3. Thomas, A., Mohindroo, C., and Giaccone, G. (2025). Advancing therapeutics in small-cell lung cancer. Nat. Cancer *6*, 938–953.

4. Lissa, D., Takahashi, N., Desai, P., Manukyan, I., Schultz, C.W., Rajapakse, V., Velez, M.J., Mulford, D., Roper, N., Nichols, S., et al. (2022). Heterogeneity of neuroendocrine transcriptional states in metastatic small cell lung cancers and patient-derived models. Nat. Commun. *13*, 2023. https://doi.org/10.1038/s41467-022-29517-9.
5. Desai, P., Takahashi, N., Kumar, R., Nichols, S., Malin, J., Hunt, A., Schultz, C., Cao, Y., Tillo, D., Nousome, D., et al. (2024). Microenvironment shapes small-cell lung cancer neuroendocrine states and presents therapeutic opportunities. Cell Rep. Med. *5*, 101610. https://doi.org/10.1016/j.xcrm.2024.101610.
6. Stewart, C.A., Gay, C.M., Xi, Y., Sivajothi, S., Sivakamasundari, V., Fujimoto, J., Bolisetty, M., Hartsfield, P.M., Balasubramaniyan, V., Chalishazar, M.D., et al. (2020). Single-cell analyses reveal increased intratumoral heterogeneity after the onset of therapy resistance in small-cell lung cancer. Nat. Cancer *1*, 423–436. https://doi.org/10.1038/s43018-019-0020-z.
7. Simpson, K.L., Stoney, R., Frese, K.K., Simms, N., Rowe, W., Pearce, S.P., Humphrey, S., Booth, L., Morgan, D., Dynowski, M., et al. (2020). A biobank of small cell lung cancer CDX models elucidates inter- and intratumoral phenotypic heterogeneity. Nat. Cancer *1*, 437–451. https://doi.org/10.1038/s43018-020-0046-2.
8. Chan, J.M., Quintanal-Villalonga, Á., Gao, V.R., Xie, Y., Allaj, V., Chaudhary, O., Masilionis, I., Egger, J., Chow, A., Walle, T., et al. (2021). Signatures of plasticity, metastasis, and immunosuppression in an atlas of human small cell lung cancer. Cancer Cell *39*, 1479–1496.e18. https://doi.org/10.1016/j.ccell.2021.09.008.
9. Rudin, C.M., Poirier, J.T., Byers, L.A., Dive, C., Dowlati, A., George, J., Heymach, J.V., Johnson, J.E., Lehman, J.M., MacPherson, D., et al. (2019). Molecular subtypes of small cell lung cancer: a synthesis of human and mouse model data. Nat. Rev. Cancer *19*, 289–297. https://doi.org/10.1038/s41568-019-0133-9.
10. Gay, C.M., Stewart, C.A., Park, E.M., Diao, L., Groves, S.M., Heeke, S., Nabet, B.Y., Fujimoto, J., Solis, L.M., Lu, W., et al. (2021). Patterns of transcription factor programs and immune pathway activation define four major subtypes of SCLC with distinct therapeutic vulnerabilities. Cancer Cell *39*, 346–360.e7. https://doi.org/10.1016/j.ccell.2020.12.014.
11. Thomas, A., Takahashi, N., Rajapakse, V.N., Zhang, X., Sun, Y., Ceribelli, M., Wilson, K.M., Zhang, Y., Beck, E., Sciuto, L., et al. (2021). Therapeutic targeting of ATR yields durable regressions in small cell lung cancers with high replication stress. Cancer Cell *39*, 566–579.e7. https://doi.org/10.1016/j.ccell.2021.02.014.
12. Roper, N., Velez, M.J., Chiappori, A., Kim, Y.S., Wei, J.S., Sindiri, S., Takahashi, N., Mulford, D., Kumar, S., Ylaya, K., et al. (2021). Notch signaling and efficacy of PD-1/PD-L1 blockade in relapsed small cell lung cancer. Nat. Commun. *12*, 3880. https://doi.org/10.1038/s41467-021-24164-y.
13. Nabet, B.Y., Hamidi, H., Lee, M.C., Banchereau, R., Morris, S., Adler, L., Gayevskiy, V., Elhossiny, A.M., Srivastava, M.K., Patil, N.S., et al. (2024). Immune heterogeneity in small-cell lung cancer and vulnerability to immune checkpoint blockade. Cancer Cell *42*, 429–443.e4.
14. Liu, Q., Zhang, J., Guo, C., Wang, M., Wang, C., Yan, Y., Sun, L., Wang, D., Zhang, L., Yu, H., et al. (2024). Proteogenomic characterization of small cell lung cancer identifies biological insights and subtype-specific therapeutic strategies. Cell *187*, 184–203.e28. https://doi.org/10.1016/j.cell.2023.12.004.
15. Zhang, W., Girard, L., Zhang, Y.A., Haruki, T., Papari-Zareei, M., Stastny, V., Ghayee, H.K., Pacak, K., Oliver, T.G., Minna, J.D., and Gazdar, A.F. (2018). Small cell lung cancer tumors and preclinical models display heterogeneity of neuroendocrine phenotypes. Transl. Lung Cancer Res. *7*, 32–49. https://doi.org/10.21037/tlcr.2018.02.02.
16. Baine, M.K., Hsieh, M.S., Lai, W.V., Egger, J.V., Jungbluth, A.A., Daneshbod, Y., Beras, A., Spencer, R., Lopardo, J., Bodd, F., et al. (2020). SCLC Subtypes Defined by ASCL1, NEUROD1, POU2F3, and YAP1: A Comprehensive Immunohistochemical and Histopathologic Characterization. J. Thorac. Oncol. *15*, 1823–1835. https://doi.org/10.1016/j.jtho.2020.09.009.
17. Park, S., Hong, T.H., Hwang, S., Heeke, S., Gay, C.M., Kim, J., Jung, H.A., Sun, J.M., Ahn, J.S., Ahn, M.J., et al. (2024). Comprehensive analysis of transcription factor-based molecular subtypes and their correlation to clinical outcomes in small-cell lung cancer. EBioMedicine *102*, 105062. https://doi.org/10.1016/j.ebiom.2024.105062.
18. Qu, S., Fetsch, P., Thomas, A., Pommier, Y., Schrump, D.S., Miettinen, M.M., and Chen, H. (2022). Molecular subtypes of primary SCLC tumors and their associations with neuroendocrine and therapeutic markers. J. Thorac. Oncol. *17*, 141–153. https://doi.org/10.1016/j.jtho.2021.08.763.
19. Wang, Z., Liu, C., Zheng, S., Yao, Y., Wang, S., Wang, X., Yin, E., Zeng, Q., Zhang, C., Zhang, G., et al. (2024). Molecular subtypes of neuroendocrine carcinomas: A cross-tissue classification framework based on five transcriptional regulators. Cancer Cell *42*, 1106–1125.e8.
20. Owonikoko, T.K., Dwivedi, B., Chen, Z., Zhang, C., Barwick, B., Ernani, V., Zhang, G., Gilbert-Ross, M., Carlisle, J., Khuri, F.R., et al. (2021). YAP1 Expression in SCLC Defines a Distinct Subtype With T-cell-Inflamed Phenotype. J. Thorac. Oncol. *16*, 464–476. https://doi.org/10.1016/j.jtho.2020.11.006.
21. Chen, H., Deng, C., Gao, J., Wang, J., Fu, F., Wang, Y., Wang, Q., Zhang, M., Zhang, S., Fan, F., et al. (2025). Integrative spatial analysis reveals tumor heterogeneity and immune colony niche related to clinical outcomes in small cell lung cancer. Cancer Cell *43*, 519–536.
22. Thomas, A., Desai, P., and Takahashi, N. (2022). Translational research: A patient-centered approach to bridge the valley of death. Cancer Cell *40*, 565–568. https://doi.org/10.1016/j.ccell.2022.04.014.
23. Drapkin, B.J., George, J., Christensen, C.L., Mino-Kenudson, M., Dries, R., Sundaresan, T., Phat, S., Myers, D.T., Zhong, J., Igo, P., et al. (2018). Genomic and Functional Fidelity of Small Cell Lung Cancer Patient-Derived Xenografts. Cancer Discov. *8*, 600–615. https://doi.org/10.1158/2159-8290.CD-17-0935.
24. Caeser, R., Egger, J.V., Chavan, S., Socci, N.D., Jones, C.B., Kombak, F.E., Asher, M., Roehrl, M.H., Shah, N.S., Allaj, V., et al. (2022). Genomic and transcriptomic analysis of a library of small cell lung cancer patient-derived xenografts. Nat. Commun. *13*, 2144. https://doi.org/10.1038/s41467-022-29794-4.
25. Corces, M.R., Granja, J.M., Shams, S., Louie, B.H., Seoane, J.A., Zhou, W., Silva, T.C., Groeneveld, C., Wong, C.K., Cho, S.W., et al. (2018). The chromatin accessibility landscape of primary human cancers. Science *362*, eaav1898. https://doi.org/10.1126/science.aav1898.
26. Horn, L., Mansfield, A.S., Szczęsna, A., Havel, L., Krzakowski, M., Hochmair, M.J., Huemer, F., Losonczy, G., Johnson, M.L., Nishio, M., et al. (2018). First-Line Atezolizumab plus Chemotherapy in Extensive-Stage Small-Cell Lung Cancer. N. Engl. J. Med. *379*, 2220–2229. https://doi.org/10.1056/NEJMoa1809064.
27. George, J., Lim, J.S., Jang, S.J., Cun, Y., Ozretić, L., Kong, G., Leenders, F., Lu, X., Fernández-Cuesta, L., Bosco, G., et al. (2015). Comprehensive genomic profiles of small cell lung cancer. Nature *524*, 47–53. https://doi.org/10.1038/nature14664.
28. Balanis, N.G., Sheu, K.M., Esedebe, F.N., Patel, S.J., Smith, B.A., Park, J.W., Alhani, S., Gomperts, B.N., Huang, J., Witte, O.N., and Graeber, T.G. (2019). Pan-cancer Convergence to a Small-Cell Neuroendocrine Phenotype that Shares Susceptibilities with Hematological Malignancies. Cancer Cell *36*, 17–34.e7. https://doi.org/10.1016/j.ccell.2019.06.005.
29. Puram, S.V., Tirosh, I., Parikh, A.S., Patel, A.P., Yizhak, K., Gillespie, S., Rodman, C., Luo, C.L., Mroz, E.A., Emerick, K.S., et al. (2017). Single-Cell Transcriptomic Analysis of Primary and Metastatic Tumor Ecosystems in Head and Neck Cancer. Cell *171*, 1611–1624.e24. https://doi.org/10.1016/j.cell.2017.10.044.
30. Ben-David, U., Ha, G., Tseng, Y.Y., Greenwald, N.F., Oh, C., Shih, J., McFarland, J.M., Wong, B., Boehm, J.S., Beroukhim, R., and Golub, T.R. (2017). Patient-derived xenografts undergo mouse-specific tumor evolution. Nat. Genet. *49*, 1567–1575. https://doi.org/10.1038/ng.3967.

31. Yu, G., Wang, L.G., and He, Q.Y. (2015). ChIPseeker: an R/Bioconductor package for ChIP peak annotation, comparison and visualization. Bioinformatics *31*, 2382–2383. https://doi.org/10.1093/bioinformatics/btv145.

32. Travaglini, K.J., Nabhan, A.N., Penland, L., Sinha, R., Gillich, A., Sit, R.V., Chang, S., Conley, S.D., Mori, Y., Seita, J., et al. (2020). A molecular cell atlas of the human lung from single-cell RNA sequencing. Nature *587*, 619–625. https://doi.org/10.1038/s41586-020-2922-4.

33. Fukushima, T., Togasaki, K., Hamamoto, J., Emoto, K., Ebisudani, T., Mitsuishi, A., Sugihara, K., Shinozaki, T., Okada, M., Saito, A., et al. (2025). An organoid library unveils subtype-specific IGF-1 dependency via a YAP-AP1 axis in human small cell lung cancer. Nat. Cancer *6*, 874–891. https://doi.org/10.1038/s43018-025-00945-y.

34. Kreis, J., Aybey, B., Geist, F., Brors, B., and Staub, E. (2024). Stromal Signals Dominate Gene Expression Signature Scores That Aim to Describe Cancer Cell-intrinsic Stemness or Mesenchymality Characteristics. Cancer Res. Commun. *4*, 516–529. https://doi.org/10.1158/2767-9764.CRC-23-0383.

35. Neptune, E.R., Podowski, M., Calvi, C., Cho, J.H., Garcia, J.G.N., Tuder, R., Linnoila, R.I., Tsai, M.J., and Dietz, H.C. (2008). Targeted disruption of NeuroD, a proneural basic helix-loop-helix factor, impairs distal lung formation and neuroendocrine morphology in the neonatal lung. J. Biol. Chem. *283*, 21160–21169. https://doi.org/10.1074/jbc.M708692200.

36. Ball, D.W., Azzoli, C.G., Baylin, S.B., Chi, D., Dou, S., Donis-Keller, H., Cumaraswamy, A., Borges, M., and Nelkin, B.D. (1993). Identification of a human achaete-scute homolog highly expressed in neuroendocrine tumors. Proc. Natl. Acad. Sci. USA *90*, 5648–5652. https://doi.org/10.1073/pnas.90.12.5648.

37. Guillemot, F. (1999). Vertebrate bHLH genes and the determination of neuronal fates. Exp. Cell Res. *253*, 357–364. https://doi.org/10.1006/excr.1999.4717.

38. Zhang, H., Liu, C.Y., Zha, Z.Y., Zhao, B., Yao, J., Zhao, S., Xiong, Y., Lei, Q.Y., and Guan, K.L. (2009). TEAD transcription factors mediate the function of TAZ in cell growth and epithelial-mesenchymal transition. J. Biol. Chem. *284*, 13355–13362. https://doi.org/10.1074/jbc.M900843200.

39. Levings, D.C., Lacher, S.E., Palacios-Moreno, J., and Slattery, M. (2021). Transcriptional reprogramming by oxidative stress occurs within a predefined chromatin accessibility landscape. Free Radic. Biol. Med. *171*, 319–331. https://doi.org/10.1016/j.freeradbiomed.2021.05.016.

40. Tantin, D. (2013). Oct transcription factors in development and stem cells: insights and mechanisms. Development *140*, 2857–2866. https://doi.org/10.1242/dev.095927.

41. Tamura, T., Kurotaki, D., and Koizumi, S.i. (2015). Regulation of myelopoiesis by the transcription factor IRF8. Int. J. Hematol. *101*, 342–351. https://doi.org/10.1007/s12185-015-1761-9.

42. Kurachi, M., Barnitz, R.A., Yosef, N., Odorizzi, P.M., DiIorio, M.A., Lemieux, M.E., Yates, K., Godec, J., Klatt, M.G., Regev, A., et al. (2014). The transcription factor BATF operates as an essential differentiation checkpoint in early effector CD8 T cells. Nat. Immunol. *15*, 373-+.

43. Miranda, A., Hamilton, P.T., Zhang, A.W., Pattnaik, S., Becht, E., Mezheyeuski, A., Bruun, J., Micke, P., de Reynies, A., and Nelson, B.H. (2019). Cancer stemness, intratumoral heterogeneity, and immune response across cancers. Proc. Natl. Acad. Sci. USA *116*, 9020–9029. https://doi.org/10.1073/pnas.1818210116.

44. Palmer, N.P., Schmid, P.R., Berger, B., and Kohane, I.S. (2012). A gene expression profile of stem cell pluripotentiality and differentiation is conserved across diverse solid and hematopoietic cancers. Genome Biol. *13*, R71. https://doi.org/10.1186/gb-2012-13-8-r71.

45. Ben-Porath, I., Thomson, M.W., Carey, V.J., Ge, R., Bell, G.W., Regev, A., and Weinberg, R.A. (2008). An embryonic stem cell-like gene expression signature in poorly differentiated aggressive human tumors. Nat. Genet. *40*, 499–507. https://doi.org/10.1038/ng.127.

46. Shats, I., Gatza, M.L., Chang, J.T., Mori, S., Wang, J., Rich, J., and Nevins, J.R. (2011). Using a stem cell-based signature to guide therapeutic selection in cancer. Cancer Res. *71*, 1772–1780. https://doi.org/10.1158/0008-5472.CAN-10-1735.

47. Gulati, G.S., Sikandar, S.S., Wesche, D.J., Manjunath, A., Bharadwaj, A., Berger, M.J., Ilagan, F., Kuo, A.H., Hsieh, R.W., Cai, S., et al. (2020). Single-cell transcriptional diversity is a hallmark of developmental potential. Science *367*, 405–411. https://doi.org/10.1126/science.aax0249.

48. Hoshida, Y. (2010). Nearest template prediction: a single-sample-based flexible class prediction with confidence assessment. PLoS One *5*, e15543. https://doi.org/10.1371/journal.pone.0015543.

49. Tang, F., Xu, D., Wang, S., Wong, C.K., Martinez-Fundichely, A., Lee, C.J., Cohen, S., Park, J., Hill, C.E., Eng, K., et al. (2022). Chromatin profiles classify castration-resistant prostate cancers suggesting therapeutic targets. Science *376*, eabe1505. https://doi.org/10.1126/science.abe1505.

50. Joanito, I., Wirapati, P., Zhao, N., Nawaz, Z., Yeo, G., Lee, F., Eng, C.L.P., Macalinao, D.C., Kahraman, M., Srinivasan, H., et al. (2022). Single-cell and bulk transcriptome sequencing identifies two epithelial tumor cell states and refines the consensus molecular classification of colorectal cancer. Nat. Genet. *54*, 963–975. https://doi.org/10.1038/s41588-022-01100-4.

51. Heeke, S., Gay, C.M., Estecio, M.R., Tran, H., Morris, B.B., Zhang, B., Tang, X., Raso, M.G., Rocha, P., Lai, S., et al. (2024). Tumor- and circulating-free DNA methylation identifies clinically relevant small cell lung cancer subtypes. Cancer Cell *42*, 225–237.e5. https://doi.org/10.1016/j.ccell.2024.01.001.

52. Ayers, M., Lunceford, J., Nebozhyn, M., Murphy, E., Loboda, A., Kaufman, D.R., Albright, A., Cheng, J.D., Kang, S.P., Shankaran, V., et al. (2017). IFN-gamma-related mRNA profile predicts clinical response to PD-1 blockade. J. Clin. Investig. *127*, 2930–2940. https://doi.org/10.1172/JCI91190.

53. McColl, K., Wildey, G., Sakre, N., Lipka, M.B., Behtaj, M., Kresak, A., Chen, Y., Yang, M., Velcheti, V., Fu, P., and Dowlati, A. (2017). Reciprocal expression of INSM1 and YAP1 defines subgroups in small cell lung cancer. Oncotarget *8*, 73745–73756. https://doi.org/10.18632/oncotarget.20572.

54. Pongor, L.S., Schultz, C.W., Rinaldi, L., Wangsa, D., Redon, C.E., Takahashi, N., Fialkoff, G., Desai, P., Zhang, Y., Burkett, S., et al. (2023). Extrachromosomal DNA Amplification Contributes to Small Cell Lung Cancer Heterogeneity and Is Associated with Worse Outcomes. Cancer Discov. *13*, 928–949. https://doi.org/10.1158/2159-8290.CD-22-0796.

55. Meuwissen, R., Linn, S.C., Linnoila, R.I., Zevenhoven, J., Mooi, W.J., and Berns, A. (2003). Induction of small cell lung cancer by somatic inactivation of both Trp53 and Rb1 in a conditional mouse model. Cancer Cell *4*, 181–189. https://doi.org/10.1016/s1535-6108(03)00220-4.

56. Zhang, J., Zhou, N., Lin, A., Luo, P., Chen, X., Deng, H., Kang, S., Guo, L., Zhu, W., and Zhang, J. (2021). ZFHX3 mutation as a protective biomarker for immune checkpoint blockade in non-small cell lung cancer. Cancer Immunol. Immunother. *70*, 137–151. https://doi.org/10.1007/s00262-020-02668-8.

57. Thomas, A., and Pommier, Y. (2016). Small cell lung cancer: Time to revisit DNA-damaging chemotherapy. Sci. Transl. Med. *8*, 346fs12. https://doi.org/10.1126/scitranslmed.aaf6282.

58. Jia, D., Augert, A., Kim, D.W., Eastwood, E., Wu, N., Ibrahim, A.H., Kim, K.B., Dunn, C.T., Pillai, S.P.S., Gazdar, A.F., et al. (2018). Crebbp Loss Drives Small Cell Lung Cancer and Increases Sensitivity to HDAC Inhibition. Cancer Discov. *8*, 1422–1437. https://doi.org/10.1158/2159-8290.CD-18-0385.

59. Brumage, L., Best, S., Hippe, D.S., Grunblatt, E., Chanana, P., Wu, F., Lee, M.C., Ying, Z., Ibrahim, A., Chung, J.H., et al. (2025). In vivo functional screens reveal KEAP1 loss as a driver of chemoresistance in small cell lung cancer. Sci. Adv. *11*, eadq7084. https://doi.org/10.1126/sciadv.adq7084.

60. Ireland, A.S., Hawgood, S.B., Xie, D.A., Hawgood, S.B., Barbier, M.W., Zuo, L.Y., Hanna, B.E., Lucas-Randolph, S., Tyson, D.R., Witt, B.L., et al. (2024). Basal ell of origin Resolves Neuroendocrine-Tuft Lineage

CellPress
OPEN ACCESS

Plasticity in Cancer. Nature *647*, 257–267. https://doi.org/10.1038/s41586-025-09503-z.

61. Chen, J., Guanizo, A.C., Jakasekara, W.S.N., Inampudi, C., Luong, Q., Garama, D.J., Alamgeer, M., Thakur, N., DeVeer, M., Ganju, V., et al. (2023). MYC drives platinum resistant SCLC that is overcome by the dual PI3K-HDAC inhibitor fimepinostat. J. Exp. Clin. Cancer Res. *42*, 100. https://doi.org/10.1186/s13046-023-02678-1.

62. Limouse, C., Smith, O.K., Jukam, D., Fryer, K.A., Greenleaf, W.J., and Straight, A.F. (2023). Global mapping of RNA-chromatin contacts reveals a proximity-dominated connectivity model for ncRNA-gene interactions. Nat. Commun. *14*, 6073. https://doi.org/10.1038/s41467-023-41848-9.

63. Wang, H., Jin, J., Wu, J., Qu, H., Wu, S., and Bao, W. (2019). Transcriptome and chromatin accessibility in porcine intestinal epithelial cells upon Zearalenone exposure. Sci. Data *6*, 298. https://doi.org/10.1038/s41597-019-0313-1.

64. Ye, S., Ding, Y.F., Jia, W.H., Liu, X.L., Feng, J.Y., Zhu, Q., Cai, S.L., Yang, Y.S., Lu, Q.Y., Huang, X.T., et al. (2019). SET Domain-Containing Protein 4 Epigenetically Controls Breast Cancer Stem Cell Quiescence. Cancer Res. *79*, 4729–4743. https://doi.org/10.1158/0008-5472.CAN-19-1084.

65. Wingett, S.W., and Andrews, S. (2018). FastQ Screen: A tool for multi-genome mapping and quality control. F1000Res. *7*, 1338. https://doi.org/10.12688/f1000research.15931.2.

66. Bolger, A.M., Lohse, M., and Usadel, B. (2014). Trimmomatic: a flexible trimmer for Illumina sequence data. Bioinformatics *30*, 2114–2120. https://doi.org/10.1093/bioinformatics/btu170.

67. Li, H., and Durbin, R. (2009). Fast and accurate short read alignment with Burrows-Wheeler transform. Bioinformatics *25*, 1754–1760. https://doi.org/10.1093/bioinformatics/btp324.

68. Zhang, Y., Liu, T., Meyer, C.A., Eeckhoute, J., Johnson, D.S., Bernstein, B.E., Nusbaum, C., Myers, R.M., Brown, M., Li, W., and Liu, X.S. (2008). Model-based analysis of ChIP-Seq (MACS). Genome Biol. *9*, R137. https://doi.org/10.1186/gb-2008-9-9-r137.

69. Pongor, L.S., Gross, J.M., Vera Alvarez, R., Murai, J., Jang, S.M., Zhang, H., Redon, C., Fu, H., Huang, S.Y., Thakur, B., et al. (2020). BAMscale: quantification of next-generation sequencing peaks and generation of scaled coverage tracks. Epigenetics Chromatin *13*, 21. https://doi.org/10.1186/s13072-020-00343-x.

70. Dobin, A., Davis, C.A., Schlesinger, F., Drenkow, J., Zaleski, C., Jha, S., Batut, P., Chaisson, M., and Gingeras, T.R. (2013). STAR: ultrafast universal RNA-seq aligner. Bioinformatics *29*, 15–21. https://doi.org/10.1093/bioinformatics/bts635.

71. Liao, Y., Smyth, G.K., and Shi, W. (2014). featureCounts: an efficient general purpose program for assigning sequence reads to genomic features. Bioinformatics *30*, 923–930. https://doi.org/10.1093/bioinformatics/btt656.

72. McKenna, A., Hanna, M., Banks, E., Sivachenko, A., Cibulskis, K., Kernytsky, A., Garimella, K., Altshuler, D., Gabriel, S., Daly, M., and DePristo, M.A. (2010). The Genome Analysis Toolkit: a MapReduce framework for analyzing next-generation DNA sequencing data. Genome Res. *20*, 1297–1303. https://doi.org/10.1101/gr.107524.110.

73. Kim, S., Scheffler, K., Halpern, A.L., Bekritsky, M.A., Noh, E., Källberg, M., Chen, X., Kim, Y., Beyter, D., Krusche, P., and Saunders, C.T. (2018). Strelka2: fast and accurate calling of germline and somatic variants. Nat. Methods *15*, 591–594. https://doi.org/10.1038/s41592-018-0051-x.

74. McLaren, W., Gil, L., Hunt, S.E., Riat, H.S., Ritchie, G.R.S., Thormann, A., Flicek, P., and Cunningham, F. (2016). The Ensembl Variant Effect Predictor. Genome Biol. *17*, 122. https://doi.org/10.1186/s13059-016-0974-4.

75. Gaujoux, R., and Seoighe, C. (2010). A flexible R package for nonnegative matrix factorization. BMC Bioinf. *11*, 367. https://doi.org/10.1186/1471-2105-11-367.

76. Love, M.I., Huber, W., and Anders, S. (2014). Moderated estimation of fold change and dispersion for RNA-seq data with DESeq2. Genome Biol. *15*, 550. https://doi.org/10.1186/s13059-014-0550-8.

77. Hänzelmann, S., Castelo, R., and Guinney, J. (2013). GSVA: gene set variation analysis for microarray and RNA-seq data. BMC Bioinf. *14*, 7. https://doi.org/10.1186/1471-2105-14-7.

78. Rozenblatt-Rosen, O., Regev, A., Oberdoerffer, P., Nawy, T., Hupalowska, A., Rood, J.E., Ashenberg, O., Cerami, E., Coffey, R.J., Demir, E., et al. (2020). The Human Tumor Atlas Network: Charting Tumor Transitions across Space and Time at Single-Cell Resolution. Cell *181*, 236–249. https://doi.org/10.1016/j.cell.2020.03.053.

79. Domínguez Conde, C., Xu, C., Jarvis, L.B., Rainbow, D.B., Wells, S.B., Gomes, T., Howlett, S.K., Suchanek, O., Polanski, K., King, H.W., et al. (2022). Cross-tissue immune cell analysis reveals tissue-specific features in humans. Science *376*, eabl5197. https://doi.org/10.1126/science.abl5197.

80. Gao, R., Bai, S., Henderson, Y.C., Lin, Y., Schalck, A., Yan, Y., Kumar, T., Hu, M., Sei, E., Davis, A., et al. (2021). Delineating copy number and clonal substructure in human tumors from single-cell transcriptomes. Nat. Biotechnol. *39*, 599–608. https://doi.org/10.1038/s41587-020-00795-2.

81. Hao, Y., Stuart, T., Kowalski, M.H., Choudhary, S., Hoffman, P., Hartman, A., Srivastava, A., Molla, G., Madad, S., Fernandez-Granda, C., and Satija, R. (2024). Dictionary learning for integrative, multimodal and scalable single-cell analysis. Nat. Biotechnol. *42*, 293–304. https://doi.org/10.1038/s41587-023-01767-y.

82. Gu, Z., Eils, R., and Schlesner, M. (2016). Complex heatmaps reveal patterns and correlations in multidimensional genomic data. Bioinformatics *32*, 2847–2849. https://doi.org/10.1093/bioinformatics/btw313.

83. Heinz, S., Benner, C., Spann, N., Bertolino, E., Lin, Y.C., Laslo, P., Cheng, J.X., Murre, C., Singh, H., and Glass, C.K. (2010). Simple combinations of lineage-determining transcription factors prime cis-regulatory elements required for macrophage and B cell identities. Mol. Cell *38*, 576–589. https://doi.org/10.1016/j.molcel.2010.05.004.

84. Gu, Z., and Hübschmann, D. (2023). rGREAT: an R/bioconductor package for functional enrichment on genomic regions. Bioinformatics *39*, btac745. https://doi.org/10.1093/bioinformatics/btac745.

85. Li, Z., Schulz, M.H., Look, T., Begemann, M., Zenke, M., and Costa, I.G. (2019). Identification of transcription factor binding sites using ATAC-seq. Genome Biol. *20*, 45. https://doi.org/10.1186/s13059-019-1642-2.

86. Gusmao, E.G., Allhoff, M., Zenke, M., and Costa, I.G. (2016). Analysis of computational footprinting methods for DNase sequencing experiments. Nat. Methods *13*, 303–309. https://doi.org/10.1038/nmeth.3772.

87. Schep, A.N., Wu, B., Buenrostro, J.D., and Greenleaf, W.J. (2017). chromVAR: inferring transcription-factor-associated accessibility from single-cell epigenomic data. Nat. Methods *14*, 975–978. https://doi.org/10.1038/nmeth.4401.

STAR★METHODS

KEY RESOURCES TABLE

REAGENT or RESOURCE	SOURCE	IDENTIFIER
Antibodies		
ASCL1	Santa Cruz<br>Abcam, Cat# ab74065	D7<br>RRID: AB_1859937
NEUROD1	Abcam Cat# ab213725,	RRID: AB_2801303
YAP1	Santa Cruz<br>Cat# sc-101199	RRID: AB_1131430
POU2F3	Santa Cruz Biotechnology Cat# sc-293402	RRID: AB_2890011
INSM1	Santa Cruz<br>Sc-271408	A-8<br>RRID: AB_10607955
TEM 8	Abcam	EPNCI-R173-37
Biological samples		
Patient-derived xenografts (PDX)	Frederick, CAPR (Center for Advanced Preclinical Research)	phs003983.v1.p1
Critical commercial assays		
ATAC-Seq	Novogene	Illumina HiSeq 4000 platform
RNA-Seq	NCI CCR sequencing facility	Illumina HiSeq 4000
Deposited data		
ATAC-sequencing	Related to ATAC-seq based classification	dbGAP: phs003983.v1.p1
scRNA sequencing	Related to Figure 4	dbGAP: phs002371
RNA sequencing	Related to Figure 5	GSA: HRA003419
RNA sequencing	Related to Figure 6	EGA: EGAD50000000195
Experimental models: Organisms/strains		
Mouse: R6/2: B6CBA-Tg(HDexon1)62Gpb/3 J	The Jackson Laboratory	JAX: 006494
Mouse: NSG mice (NOD.Cg-Prkdc ˆscid Il2rg ˆtm1Wjl/SzJ	The Jackson Laboratory	RRID: IMSR_JAX:005557
Software and algorithms		
R	Open source	V4.3.1
R-studio	Open source	Build 554 2022.07.01
GraphPad prism	GraphPad Software	V9.3.1 (471)
Qu-Path	Open source	V0.3.0
Qlucore omics explorer	Qlucore AB	V3.8
Halo	Indica labs	v3.4.15916.175
Complex heatmap	Bioconductor	v. 2.12.1
Trimmomatic	CRAN	v.0.33
BWA2	Bioconductor	v. 0.7.17
Picard	Broad institute	v. 2.17.11
GATK	Broad institute	v.4.2.2.0
Strelka2	Broad institute	V2.9.10
maftools	Bioconductor	v.2.12.0
DESeq2	Bioconductor	v.1.50.2
Addmodulescore	Seurat	v.4.4.0
NMF	Bioconductor	v.0.23.0
Suerat	Bioconductor	v.5.1.0

(*Continued on next page*)

Continued

REAGENT or RESOURCE	SOURCE	IDENTIFIER
CytoTRACE	GitHub	v.1.1.0
TOBIAS	GitHub	v.0.14.0
Python	Python software foundation	v.3.8.0
GSEApy	Github	v.1.3.0

EXPERIMENTAL MODEL AND STUDY PARTICIPANT DETAILS

Human samples

Fresh tumor biopsies were obtained from patients with small-cell lung cancer enrolled on protocol NCT02146170, which was approved by the NIH Institutional Review Board (IRB 14-*c*-0105). All patients provided written informed consent, and additional consent was obtained from next of kin when applicable. Patient demographic and clinical characteristics, including age at diagnosis, sex, disease stage, smoking history, and prior treatment history, are summarized in Table S1. Tumor biopsies were performed at the NIH clinical center by laboratory of pathology staff. A total of 31 biopsies from 27 unique patients were collected, processed, and stored according to protocol-defined procedures. The collected biopsies were allocated for patient-derived xenograft (PDX) establishment, and successfully engrafted tumors were subsequently used for ATAC-sequencing and RNA sequencing. Both male and female patients were included in this study. Sex was recorded as part of the clinical characteristics (Table S1); however, the study was not designed or powered to evaluate sex-specific differences, and analyses were therefore not stratified by sex.

Animal studies

Fresh tumor specimens were implanted subcutaneously into eight-week-old male or female NSG mice (NOD.Cg-Prkdc ˆscid Il2rg ˆtm1Wjl/SzJ; #005557, Jackson Laboratory) to generate patient-derived xenograft (PDX) models and were serially propagated through passages P1–P3. Mouse handling and experimental procedures were conducted under an approved animal study protocol #23–044 in accordance with frederick national laboratory animal care and use committee guidelines. NCI-Frederick is accredited by AAALAC International and follows the Public Health Service Policy for the Care and Use of Laboratory Animals. Animal care was provided in accordance with the procedures outlined in the Guide for the Care and Use of Laboratory Animals (National Research Council, 2011; National Academies Press, Washington, DC). Animals were housed under a 12-h light/dark cycle with controlled temperature (68–74°F) and humidity (30–70%) and monitored daily. Of 31tumor biopsies implanted, 21 leds to successful PDX model generation and subjected to ATAC-sequencing and RNA sequencing. Both male and female NSG mice were used for PDX establishment. The study was not designed to evaluate the effect of host sex on PDX engraftment or downstream molecular analyses, and therefore no sex-specific analyses were performed.

METHOD DETAILS

Human samples for PDX model development

Fresh tumor biopsies were obtained on NCT02146170 which was approved by the NIH Institutional Review Board. All patients provided informed consent. In total, 31 tumor biopsies were performed from 27 unique patients (TMSCLC-01,06, 09, and 22 had biopsies at two separate timepoints). TMSCLC09 had cyst contents harvested into 2 aliquots in lieu of solid biopsy core, and both were maintained independently, designated TMSLC09-1 and 09–2. Ten of the 31 biopsies failed to engraft as subcutaneous tumors in NSG mice. For each case, only a single needle biopsy was available for implantation into one mouse at the P1 stage. Factors contributing to engraftment failure likely included limited tissue volume, low tumor cellularity, delays between sampling and implantation, and health issues in recipient mice necessitating euthanasia per ASP and ACUC protocols.

PDX generation

Eight-week-old male or female NSG mice (NOD.Cg-Prkdc ˆscid Il2rg ˆtm1Wjl/SzJ; #005557, Jackson Laboratory) were subcutaneously implanted with fresh patient needle biopsies mixed with Matrigel (Corning) at the Center for Advanced Preclinical Research (CAPR) at the Frederick National Laboratory for Cancer Research. Mice were monitored daily, with caliper measurements and body weights performed twice weekly. Once tumors reached the size limit (2000 mm^3) or mice became moribund, the mouse was euthanized, and a 3×3 mm^3 tumor fragment was transplanted into a new mouse (P2). This process was repeated to achieve a minimum of passage three (P3) for each patient-derived origin. Samples from each passage were collected for histological analysis (fixed in 10% neutral buffered formalin), flash-frozen for molecular studies, and cryopreserved for viable storage. Of 31 tumor biopsies implanted, 21 leds to successful PDX model generation. The PDX models were characterized for *in vivo* consistency, histopathological reliability, and similarity to the originating patient tumor pathology. Mouse handling and procedures were conducted in accordance with an approved Animal Study Protocol, adhering to Frederick National Laboratory Animal Care and Use Committee

guidelines. Mice were housed under a 12-h light/dark cycle, with room temperatures maintained between 68 and 74°F and humidity levels between 30 and 70%.

Immunohistochemistry

Paraffin-embedded tumor sections were prepared and subjected to citrate-based antigen retrieval prior to primary antibody staining. Staining was performed against the following proteins: NeuroD1 (1:100, Abcam, Cat# ab213725, RRID:AB_2801303), MASH1 (1:750, Abcam, Cat# ab74065, RRID:AB_1859937), Pou2F3(1:50, Santa Cruz Biotechnology Cat# sc-293402, RRID:AB_2890011), YAP1 (1:25, Santa Cruz Biotechnology Cat# sc-101199, RRID:AB_1131430), and INSM1 (1:100, Santa Cruz Biotechnology Cat# sc-271408, RRID:AB_10607955). All staining was carried out using the Leica BondMax Autostainer (Leica, IL). Antigen retrieval was conducted using Leica's ER1 (pH 6) retrieval buffer for 10 min (NeuroD1 and MASH1) or 20 min (Pou2F3), or Leicas ER2 (pH 9) retrieval buffer for 10 min (YAP1) or 20 min (INSM1)). Primary antibody incubation was performed for either 30 min (Neuro D1, MASH1) or 60 min (YAP1 and INSM1) at room temperature. POU2F3 was incubated overnight at 4C. Secondary antibody incubation, signal amplification, DAB development, and hematoxylin counterstaining were performed using the Leica Polymer Refine Kit (Leica, IL). Immunohistochemistry slides were scanned with the Leica Aperio AT2 scanner (RRID:SCR_021256). Scanned images were uploaded into Halo Image Analysis Software (Indica Labs, NM, RRID:SCR_018350) for automated quantification. Images were annotated to analyze viable tumor and exclude regions of necrosis and staining artifacts. Immunohistochemical staining intensity was quantified using the H-score method. For individual cells, staining intensity was classified into four levels: 0 (no staining), 1 (weak), 2 (moderate), and 3 (strong). The percentage of positively stained cells at each intensity level was determined. The H-score for each sample was calculated by multiplying each staining intensity score by the corresponding percentage of cells at that intensity and subsequently summing the resulting values. The final scores ranged from 0 to 300.

ATAC-seq

Xenograft samples were harvested during exponential growth to minimize necrosis and ensure high-quality tissue. ATAC-seq was performed on frozen samples using an established and well-validated protocol optimized for cancer tissues. The workflow was adapted from previously published protocols with standardized nuclei lysis and titrated Tn5 transposition.[25,62–64] This protocol utilizes multiple detergents (NP40, Tween 20, Digitonin) and a post-lysis wash to reduce mitochondrial contamination and background, improving signal-to-noise ratio for frozen tissues. For each xenograft, 50 mg of tissue (minimum 20 mg) was processed. Samples underwent quality control prior to nuclear extraction and again following library construction. Input material met predefined quality criteria, including sufficient DNA yield and concentration, absence of degradation or contamination, and appropriate purity as assessed by NanoDrop and gel electrophoresis. Xenografts that did not meet these criteria were excluded and replaced with a quality-controlled passage of the corresponding model. Library quality was further evaluated based on fragment-size distribution, library complexity, and enrichment prior to sequencing. Libraries were sequenced as paired end 150 bp reads on an Illumina HiSeq 4000 platform. Following sequencing, raw FASTQ files containing the reads were subjected to quality control using FastQC[65] prior any downstream analysis. Preprocessing included trimming and filtering to retain only high-quality reads; low-quality bases and adapter sequences were removed using Trimmomatic.[66] Trimmed reads were aligned to the human genome reference assembly (hg19) using the Burrows-Wheeler Aligner.[67] PCR duplicates were identified and marked using Picard tools (https://broadinstitute.github.io/picard/) to ensure that downstream analyses were restricted to unique fragments. Reads mapping to mitochondrial DNA were removed to focus on nuclear chromatin accessibility. Peak calling was performed using MACS2 (Model-based Analysis of ChIP-Seq)[68] to identify regions of open chromatin indicative of potential regulatory elements, such as promoters or enhancers. A consensus matrix of accessible chromatin regions across all PDX samples was generated using the BAMscale tool[69] enabling integrated analysis of chromatin accessibility and capturing biologically relevant regulatory regions across the cohort.

RNA-seq

The same biopsy samples used for ATAC-seq were utilized for RNA-seq library preparation and sequencing, following the Illumina HiSeq protocol (NCI CCR Sequencing Facility). Initially, raw FASTQ files underwent processing with Trimmomatic[66] to remove adapter sequences and low-quality reads. The trimmed reads were then aligned to the human genome (hg19) using STAR aligner (version 2.4.2a).[70] Gene expression levels were quantified from the aligned reads using Rsubread featureCounts.[71] The raw counts were subsequently normalized to Transcripts Per Million (TPM) to enable comparison of expression levels across samples, mitigating variability due to sequencing depth or gene length. To validate PDX stability across passages, we performed correlation analysis on matched samples ($n = 35$) using the top 1,000 most variable genes. Pairwise Pearson correlations were computed on log2-transformed expression data, and hierarchical clustering was performed using correlation-based distances (1 - Pearson correlation, average linkage). Expression stability of core SCLC transcriptional markers (ASCL1, NEUROD1, INSM1, CHGA, SYP, NCAM1, POU2F3, YAP1, EPCAM, DLL3) was assessed by Z score normalization per gene and visualization across passages for each PDX model.

Whole exome data and somatic mutation analysis

The whole exome paired-end matched tumor-normal dataset of limited stage SCLC was obtained from Liu et al.[14] with authorized access through the National Genomics Data Center (HRA003419). To identify single nucleotide variants (SNVs) and small insertions/deletions (INDELs), whole exome sequencing data underwent comprehensive quality control and variant calling procedures using the

CellPress
OPEN ACCESS

XAVIER (https://zenodo.org/records/15473339) pipeline with default parameters. In short, quality-filtered paired-end sequencing reads were aligned to the human reference genome (hg38) using BWA-MEM algorithm.[67] The resulting alignment files were processed through GATK modules[72] for base quality score recalibration and duplicate removal. Somatic variant was detected using Strelka2[73] on matched tumor-normal sample pairs. The identified variants were filtered through GATK SelectVariants and VariantFiltration modules. Finally, the variant annotation was performed using VEP[74] to characterize functional consequences.

Identification of features from ATAC-seq and determination of optimal clusters

Peaks identified by the MACS2 peak caller[68] were utilized to map open chromatin regions. Annotation of peaks to their nearest neighbor genes was performed using the default ChIPseeker[31] settings, spanning −3000 to +3000 bp from transcription start sites. The annotation process involves default hierarchical feature assignment, prioritizing promoter regions (±1000 bp from transcription start site) followed by UTRs, exons, introns, and intergenic regions. Gene annotations for peak annotation were derived from UCSC genome using the hg19 reference genome, with peak-to-gene distances calculated using standard interval overlap methods. Among the identified ATAC-seq peaks, promoter peaks accounted for 37.06%, while distal peaks comprised 57.23%, indicating a predominant enrichment in distal regulatory elements within our dataset. Unsupervised clustering of patient samples was conducted using Non-Negative Matrix Factorization via NMF R package (version 0.23.0),[75] using the 'brunet' factorization method with open chromatin matrices created from the ATAC-seq data as input. We explored cluster numbers (k) ranging from 2 to 8, performing 50 repetitions and 1,000 iterations for each value of k to ensure reproducibility and reliability. Optimal clustering was determined using multiple stability metrics, including cophenetic correlation, dispersion, and mean silhouette width, which consistently identified k = 3 as the most stable solution. Once the optimal k was established (k = 3), we reran the NMF analysis using top 1% most variable peaks obtained using median absolute deviation (MAD) to ensure the results was stable and robust. To assess robustness, we performed a bootstrap-based stability analysis of the k = 3, using 200 replicates under sample, feature, and joint subsampling (80% each), with stability quantified by the adjusted Rand index (ARI). To assess robustness, we repeated NMF clustering using the top 1%, 5%, and 10% of variable peaks, which yielded highly concordant subtype assignments. Unsupervised clustering using NMF yielded a single set of three chromatin-based clusters (k = 3), derived from the top 1% most variable ATAC-seq peaks and consistently recovered across biological replicates. As summarized in the concept schematic (Figure S2A), these NMF-defined chromatin clusters represent the sole clustering solution generated from ATAC-seq data. To assess the impact of within-model variability, NMF clustering was repeated after excluding samples with discordant cluster assignments across passages, and stability was evaluated using cophenetic correlation, dispersion, silhouette metrics, and consensus structure. Cluster-specific subtype signatures were derived by linking cluster-enriched ATAC-seq peaks to their nearest genes using ChIPseeker, thereby defining chromatin-informed gene sets associated with each cluster. Matched bulk RNA-seq data were subsequently used to quantify and project these chromatin-derived gene signatures (Table S6) across independent datasets for validation. Based on enrichment of immune-associated, stem-like, and neuroendocrine regulatory programs, the three chromatin clusters were annotated as SCLC-IM, SCLC-SL, and SCLC-NE, respectively. Importantly, the IM/SL/NE nomenclature reflects biological interpretation of the underlying chromatin clusters and does not represent an independent or secondary clustering.

Differential chromatin accessibility and cluster-specific features

Differential chromatin accessibility across clusters identified by NMF was analyzed using DESeq2.[76] Regions with significant chromatin accessibility differences were identified with thresholds of absolute log2FoldChange >1 and P value <0.05. These cluster-specific accessible peaks were then linked to potential gene regulatory elements unique to each cluster. For lineage enrichment analyses, Fisher's exact tests were used to compare the overlap of lineage-associated genes among significantly differentially accessible peaks versus background peaks, and enrichment was quantified as the $\log_2$-transformed odds ratio, which was used for visualization and comparison across chromatin-defined subtypes.

Differential gene expression and pathway enrichment analysis

Differential gene expression analysis between clusters was conducted using DESeq2.[76] Cluster-specific genes were identified by comparing each cluster to the remaining samples, applying thresholds of absolute log2FoldChange >2 and P value <0.05. To uncover biological pathways associated with each cluster, we performed Gene Set Enrichment Analysis (GSEA) using the Broad Molecular Signature Database gene sets. Single-sample GSEA (ssGSEA) enrichment scores were computed using the GSVA R/Bioconductor package.[77] Gene-sets used in this study and source information are in Table S8.

Analysis of single-cell RNA-seq data

scRNA-seq data for 19 SCLC samples[8] was obtained from the HTAN[78] data portal. Tumor cells were selected based on annotation of cells using CellTypist[79] and inferred copy number status by CopyKAT version 1.1.0.[80] Tumor samples were integrated using the SCTransform method from Seurat version 5.1.0,[81] and tumor cells were selected for further analysis. Single-cell activity of pathways was calculated using the AddModuleScore of Seurat, while single sample gene set enrichment analysis (ssGSEA) activity of gene sets was calculated on the generated bulk expression data of each patient using the ssgsea function of the GSVA package version 1.50.5.[77] Heatmaps were generated using the ComplexHeatmap package version 2.18.0.[82]

Motif enrichment

Motif analysis was performed using the HOMER algorithm[83] (findMotifsGenome.pl) with 'size -given -mask' criteria to identify regulatory elements associated with specific clusters. Differentially accessible peaks were further analyzed for pathway enrichment using the Genomic Regions Enrichment of Annotations Tool (GREAT).[84] Transcription factor binding motifs were obtained from the JASPAR 2022 database (human, non-redundant) and matched to accessible chromatin regions. Motif deviation scores, which represent accessibility at motif-containing peaks relative to matched background peaks, were computed for each motif in each sample.

Construction of gene regulatory networks

We constructed regulatory networks to link open chromatin, gene expression, and transcription factor binding sites (TFBS) using a method similar to that described by Tang et al.[49] To link open chromatin to gene expression, we performed a Pearson correlation test between ATAC-seq peaks within ±0.5 Mb of a gene's transcription start site (TSS) and the normalized RNA-seq counts of that gene. We retained peak-gene pairs with an FDR <0.01. Additionally, foot printing analysis was performed using HINT-ATAC[85,86] to link TFBS with ATAC-seq peaks. The final network was constructed by combining peak-gene links with TF-peak links to infer the networks of TFs and the genes they regulate.

To rank the TFs, we utilized the scoring system developed by Tang et al.[49] The ranking was based on three metrics: (1) outdegree, which represents the number of target genes for a TF in the constructed regulatory networks for each sample, (2) chromatin accessibility of TFBS, computed using chromVAR[87] as the change in accessibility based on ATAC-seq peaks at TFBS relative to the average accessibility across samples, and (3) differential gene expression of TF genes, computed using DESeq2[76] for log2FoldChange from RNA-seq data. Each TF was independently ranked according to these metrics, and the scores were summed across the three metrics to generate an overall TF rank.

Subtype assignment of SCLC datasets

We identified signature genes for our cohort by distinguishing three clusters through differential expression analysis with DESeq2,[76] using stringent criteria of log2FoldChange >2 and adjusted P value (padj) < 0.01. These signature genes were then used to classify independent SCLC cohorts. To ensure optimal classification, we selected the top 50 most discriminative signature genes based on their expression patterns, showing higher expression in one cluster compared to others. For subtype classification of the independent SCLC cohorts, we utilized the Nearest Template Prediction (NTP) algorithm.[48] This method calculated the cosine distance (d) between each tumor's expression profile and our predefined signatures. Statistical significance was determined through 1,000 iterations of random gene sampling from the patient expression data. To avoid forcing discrete assignments, NTP was used to compute probability estimates for all three subtypes for every sample. Classification probabilities were derived from distance metrics using a softmax transformation, and multiple testing correction was applied using false discovery rate (FDR) adjustment. To explicitly account for biological heterogeneity and cross-platform differences, we defined an ambiguity criterion based on classification confidence. Samples were classified as ambiguous if the maximum subtype probability was <0.5 or if the confidence margin between the top two subtype probabilities was <0.2. For each sample, we report: (i) probabilities for all three subtypes, (ii) the maximum assignment probability, (iii) the confidence margin between the top two subtypes, (iv) an ambiguity flag (Is_Ambiguous: TRUE/FALSE), and (v) FDR-adjusted permutation p values. Ambiguous samples were retained in downstream analyses and interpreted as reflecting intermediate or mixed regulatory states rather than classification failures. All probabilistic subtype assignments and ambiguity metrics are provided in Table S9.

Extensive stage immunotherapy cohort

TPM normalized RNA expression data (EGAD50000000196), clinical data (EGAD50000000195) and subtype assignments (EGAD00001006926) for the IMpower133 were obtained through the European Genome-Phenome Archive (EGA, https://ega-archive.org/) under the EGAS50000000138[13] and EGAS00001004888[10] study IDs.

Other datasets

RNA-seq data for limited-stage SCLC patients were obtained from the Genome Sequence Archive of the National Genomic Data Center, China (HRA00341915). Data from treatment-naïve SCLC patients (George et al.) were sourced from EGAS00001000925,[27] with additional samples from the database of Genotype and Phenotype accession phs003416.v1. p1(Heeke et al.).[51] NCI RNA-seq dataset includes extensive-stage, relapsed, SCLC from patients recruited on NCT02146170 which was approved by the NIH Institutional Review Board. All patients provided informed consent.

Survival analysis

Patient characteristics were categorized based on clusters and compared using Fisher's exact test or chi-square test for categorical data, and the Wilcoxon rank-sum test for continuous data. Categorical variables were summarized as frequencies and percentages, while continuous variables (e.g., age) were described using summary statistics such as median, minimum, and maximum values. Overall survival data was obtained from trial records. Univariate and multivariable Cox proportional hazards models were used to evaluate the impact of potential risk factors on OS, incorporating statistically significant variables into the multivariable models. Hazard ratios with corresponding 95% confidence intervals were reported. Kaplan-Meier curves were generated to estimate survival

distributions by cluster status, with the Log rank test used to compare survival distributions across treatment groups. All statistical tests were two-tailed, with P values <0.05 considered statistically significant. Analyses were performed using R (version 4.3.0), utilizing the 'survival' and 'survminer' packages.

QUANTIFICATION AND STATISTICAL ANALYSIS

All statistical analyses were performed using R (version 4.3.1) unless otherwise specified. Data visualization and statistical analyses were conducted using R and GraphPad Prism (version 9.3.1). Gene set enrichment analyses were performed as described in the Method Details section, and GSEA enrichment plots were generated using GSEApy within a Python 3.8 environment. Statistical details for individual experiments, including the statistical tests used, exact sample sizes (n), the definition of n, and significance thresholds, are provided in the corresponding figure legends, Results section, and STAR Methods. For PDX studies, n represents individual tumor samples or biological replicates as indicated. For clinical cohort analyses, n represents individual patients. For single-cell RNA sequencing analyses, n represents patient samples, as specified in the relevant figures and methods. Differential chromatin accessibility and differential gene expression analyses were performed using DESeq2 (version 1.50.2). Pearson correlation analysis was used to assess associations between chromatin accessibility and gene expression. Comparisons among multiple groups were performed using Tukey's multiple-comparison test. Categorical variables were compared using Fisher's exact test or chi-square test, while continuous variables were analyzed using the Wilcoxon rank-sum test. Survival outcomes were evaluated using Kaplan-Meier analyses with two-sided log rank tests, and univariate and multivariable Cox proportional hazards models were used to estimate hazard ratios (HRs) with 95% confidence intervals (CIs). Unless otherwise stated, all statistical tests were two-sided and *p* values <0.05 were considered statistically significant. Data are presented as median values, hazard ratios with 95% confidence intervals, enrichment scores, or other summary statistics as indicated in the corresponding figures and figure legends.

Cell Reports Medicine, Volume *7*

Supplemental information

A stem-like chromatin program in small-cell lung cancer is associated with poor outcomes after chemoimmunotherapy

Rajesh Kumar, Parth Desai, Rajaa El Meskini, Chirayu Mohindroo, Alexandra Ágnes Benő, Devon Atkinson, Michael Nirula, Laura Bassel, Nobuyuki Takahashi, Kelly L. Sovacool, Anna Meinhardt, Mohit Arora, Kanak Parmar, Darryl Nausome, Samantha Nichols, Linda Sciuto, Shannon Swift, Kolos Nemes, Ajit Kumar Sharma, Nathan Pate, Arun Rajan, Elliot Levy, Michael Kassin, Christopher Febres Aldana, Vishal Koparde, Melissa Galloux, Roshan Shreshta, Zoe Weaver Ohler, Lorinc Pongor, and Anish Thomas

Fig. S1

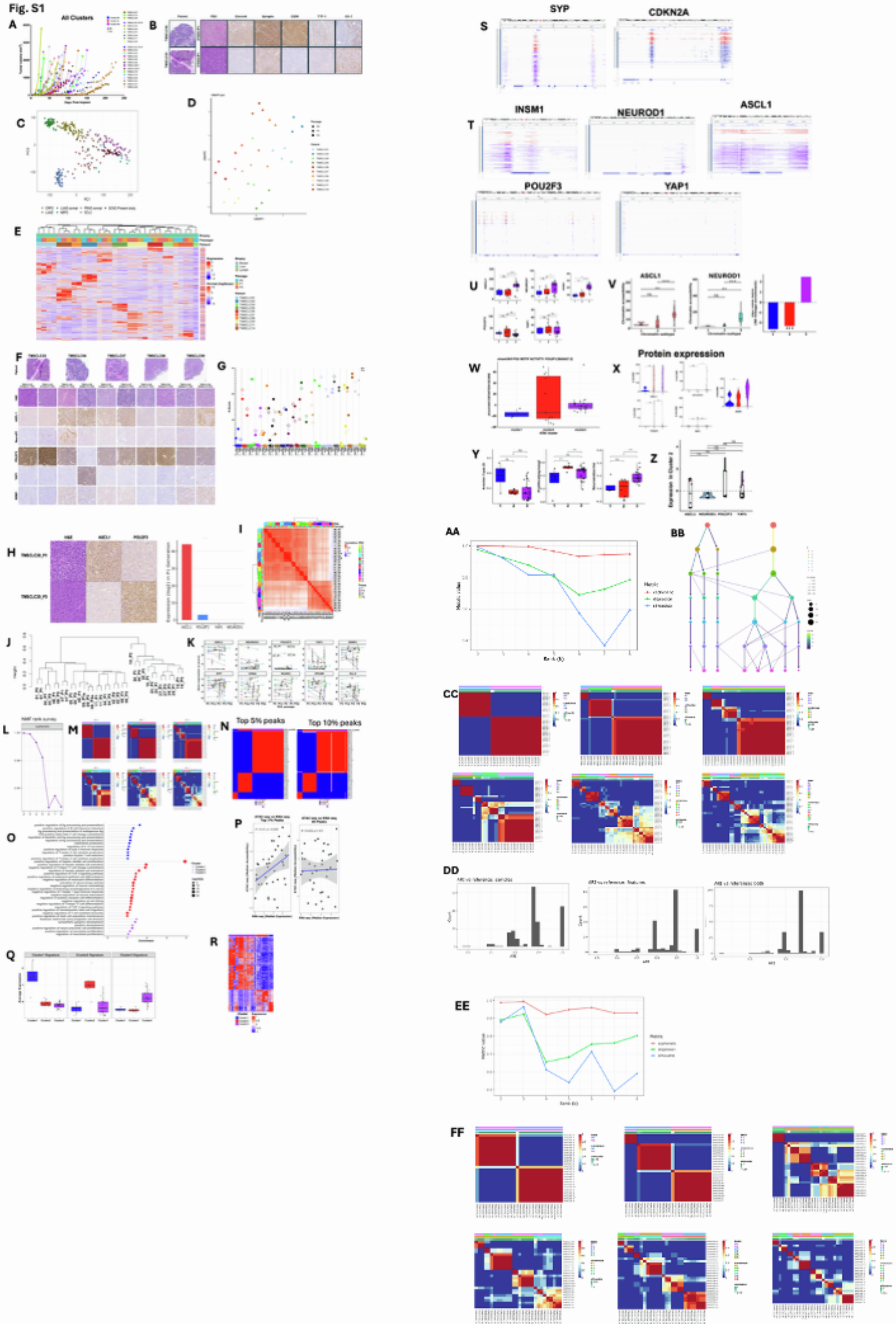
A
All Clusters
B
C
D
E
F
G
H
H&E
ASCL1
POU2F3
I
J
K
L
NMF rank survey
M
N
Top 5% peaks
Top 10% peaks
O
P
Q
R
S
SYP
CDKN2A
T
INSM1
NEUROD1
ASCL1
POU2F3
YAP1
U
V
ASCL1
NEUROD1
W
X
Protein expression
Y
Z
AA
Metric
Rank (k)
BB
CC
DD
EE
FF

Figure S1. Validation of PDX models and stability of chromatin accessibility-informed regulatory programs and NMF clustering in SCLC, related to Figure 1

A. Growth kinetics of PDX tumors.
B. H/E and IHC analysis of key SCLC diagnostic markers (chromogranin A, synaptophysin, CD56, TTF-1, and/or CK-7) in patient-derived tumors and their corresponding first-generation PDX tumors (P1). For further details, see Tables S1 and S2. Scale bars, 100 µm.
C. Pan-cancer rPCA projection of transcriptomic data from the current study, co-clustering with known SCLC tumors[28].
D. UMAP of transcriptomic data (top 1% most variable genes), showing co-localization of P0 and corresponding PDX tumors.
E. Heatmap generated after removal of TME genes (using an approach described previously[29]) displaying co-clustering of P0 and PDX tumors.
F. H/E staining of patient-origin tumors (on top) and corresponding PDX tumors (P1, P3), alongside IHC of key SCLC lineage-defining markers (ASCL1, NEUROD1, INSM1, YAP1, and POU2F3) across P1 and P3. See Table S3 for more details. Scale bars, 100 µm.
G. IHC quantification (H-scores) of ASCL1, NEUROD1, INSM1, YAP1, and POU2F3 in PDX tumors (P1, P3).
H. IHC staining of TMSCLC20 shows a passage-associated shift from ASCL1-high at P1 to POU2F3-high at P3 by IHC, with P1 bulk RNA-seq confirming high ASCL1 expression.
I. Pearson correlation heatmap of bulk RNA-seq profiles from patient-derived xenografts (PDXs) across multiple passages. Samples from different passages of same PDX cluster together with some variability, indicating global preservation of global transcriptional identity.
J. Hierarchical clustering showing multiple passages from the same PDX frequently co-cluster, while broader branch structure reflects inter-tumor heterogeneity, supporting the absence of global transcriptional drift during PDX propagation.
K. Expression pattern of core SCLC marker conserved among the serial passages across the matched PDX samples.
L. Cophenetic correlation index of NMF for ATAC-seq data (top 1% most variable peaks) at various k values.
M. Cross-correlation map of ATAC-seq data at various NMF resolutions, demonstrating that k=3 is the optimal resolution.
N. NMF subtype assignments are stable in PDX using the top 5% vs 10% most variable ATAC-seq peaks.
O. Chromatin-accessibility defined cluster exhibits distinct biological programs enriched for neuronal, immune and stemness pathways.
P. Correlation plot showing that concordance between the ATAC-seq and matched RNA-seq dataset for top 1% peaks (left) and all peaks (right).
Q. Boxplot depicting the specificity of genes linked to chromatin-accessibility derived peaks. Center line indicates the median; box limits indicate the first and third quartiles; whiskers represent 1.5 × IQR; points represent individual samples.
R. Heatmap showing specificity of genes linked to chromatin-accessibility derived peaks.
S. Chromatin accessibility of core SCLC markers SYP and CDKN2A.
T. Chromatin accessibility of established SCLC lineage-defining markers ASCL1, NEUROD1, YAP1, POU2F3 and INSM1.
U. Quantification of chromatin accessibility of established SCLC lineage-defining markers ASCL1, NEUROD1, YAP1, POU2F3 and INSM1. Center line indicates the median; box limits indicate the first and third quartiles; whiskers represent 1.5 × IQR; points represent individual samples.
V. Plot depicting the enrichment of ASCL1/ NEUROD1 and odd ratio enrichment of SCLC lineage markers in Cluster 3 compared to Cluster 1 and Cluster 2. Center line indicates the median; box limits indicate the first and third quartiles; whiskers represent 1.5 × IQR; points represent individual samples.
W. Motif enrichment of chromatin defined cluster reveals heterogeneous expression of POU2F3 within Cluster 2. Center line indicates the median; box limits indicate the first and third quartiles; whiskers represent 1.5 × IQR; points represent individual samples.
X. IHC quantification (H-scores) for INSM1, ASCL1, NEUROD1, POU2F3, and YAP1. Center line indicates the median; box limits indicate the first and third quartiles; whiskers represent 1.5 × IQR; points represent individual samples.

Y. Boxplot depicting enrichment of cell-type specific signature in chromatin defined clusters. Center line indicates the median; box limits indicate the first and third quartiles; whiskers represent 1.5 × IQR; points represent individual samples.

Z. Plot showing expression of canonical SCLC lineage markers (ASCL1, NEUROD1, POU2F3, and YAP1) in chromatin-defined Cluster 2. Center line indicates the median; box limits indicate the first and third quartiles; whiskers represent 1.5 × IQR; points represent individual samples.

AA. Cophenetic correlation, dispersion, and silhouette scores for NMF solutions on ATAC-seq data (top 1% most variable peaks) across values of k (n=39).

BB. Clustree analysis showing stable cluster lineage from k=2 to k=3, with increased fragmentation at higher k in PDX ATAC-seq dataset.

CC. Consensus cross correlation similarity heatmaps for NMF solutions at different k values, illustrating that k = 3 yields the most stable and well-separated clusters.

DD. Perturbation-based bootstrap stability analysis of the NMF derived k=3. Histograms show the distribution of Adjusted Rand Index (ARI) values from 200 bootstrap replicates under three perturbation condition.

EE. Plot showing cluster stability metrices (cophenetic, dispersion and silhouette) on the discordant sample removed dataset (n=28) across different values of k.

FF. Heatmap showing cluster stability at different values of NMF (k=2-8). k=3 is most stable with well-defined cluster stability blocks and increasing sub clustering yield off-diagonal signal, showing reduced cluster stability.

NMF, nonnegative matrix factorization; PCA, principal component analysis; TME, tumor microenvironment; UMAP, Uniform manifold approximation and projection; IHC, Immunohistochemistry *$p<0.05$, *** $p<0.001$, Tukey's multiple comparison test.

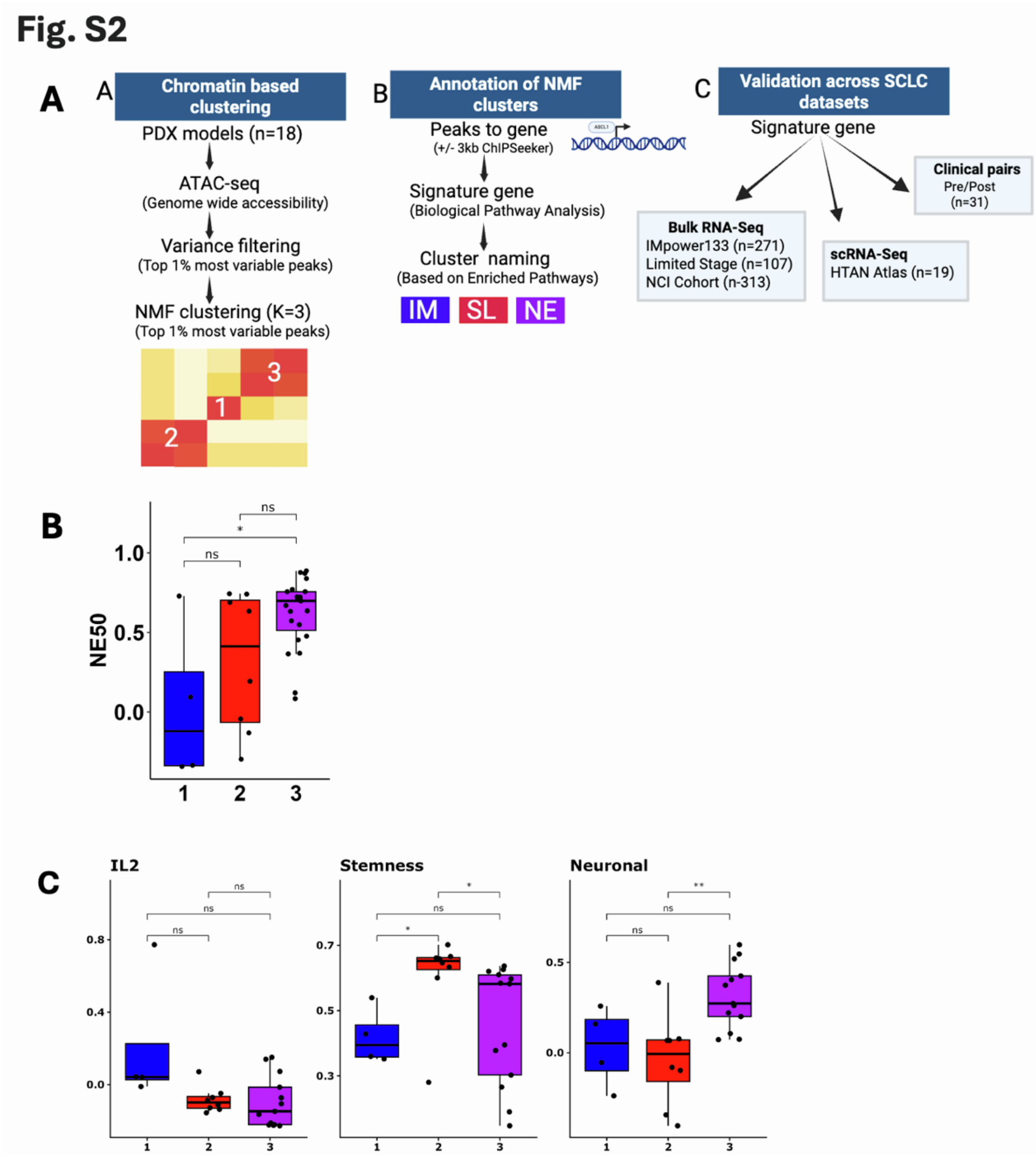

Figure S2. Computational framework and transcriptomic features supporting chromatin-defined SCLC regulatory programs, related to Figure 2

A. Concept schematic figure describing the overall computational framework for clustering, naming and cross validation strategy used in the study
B. Boxplot depicting expression of a 50-gene NE signature[15] across the three ATAC-seq-derived clusters in PDX transcriptomic dataset (n=35). Center line indicates the median; box limits indicate the first and third quartiles; whiskers represent 1.5 × IQR; points represent individual samples.

C. Pathway activity using ssGSEA analysis, showing upregulation of IL-2 signaling in cluster1, stemness signature in cluster2 and neuronal signaling in cluster3 samples. Center line indicates the median; box limits indicate the first and third quartiles; whiskers represent 1.5 × IQR; points represent individual samples. Tukey's multiple comparison test; * $p < 0.05$, ** $p < 0.005$, *** $p < 0.0001$

NE, neuroendocrine

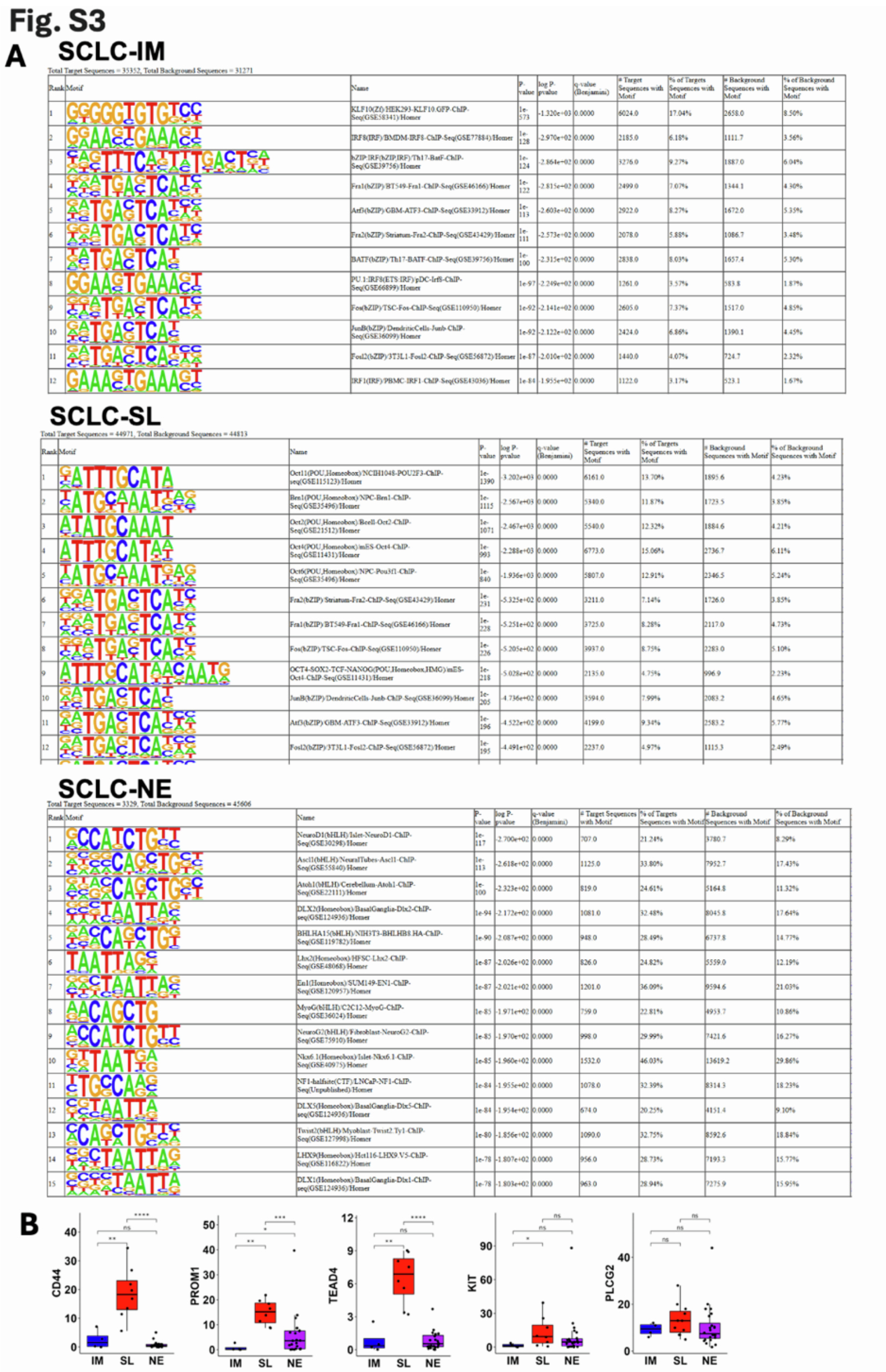

Rank	Motif	Name	P-value	log P-pvalue	q-value (Benjamini)	# Target Sequences with Motif	% of Targets Sequences with Motif	# Background Sequences with Motif	% of Background Sequences with Motif
1		KLF10(Zf)/HEK293-KLF10.GFP-ChIP-Seq(GSE58341)/Homer	1e-573	-1.320e+03	0.0000	6024.0	17.04%	2658.0	8.50%
2		IRF8(IRF)/BMDM-IRF8-ChIP-Seq(GSE77884)/Homer	1e-128	-2.970e+02	0.0000	2185.0	6.18%	1111.7	3.56%
3		bZIP:IRF(bZIP,IRF)/Th17-BatF-ChIP-Seq(GSE39756)/Homer	1e-124	-2.864e+02	0.0000	3276.0	9.27%	1887.0	6.04%
4		Fra1(bZIP)/BT549-Fra1-ChIP-Seq(GSE46166)/Homer	1e-122	-2.815e+02	0.0000	2499.0	7.07%	1344.1	4.30%
5		Atf3(bZIP)/GBM-ATF3-ChIP-Seq(GSE33912)/Homer	1e-113	-2.603e+02	0.0000	2922.0	8.27%	1672.0	5.35%
6		Fra2(bZIP)/Striatum-Fra2-ChIP-Seq(GSE43429)/Homer	1e-111	-2.573e+02	0.0000	2078.0	5.88%	1086.7	3.48%
7		BATF(bZIP)/Th17-BATF-ChIP-Seq(GSE39756)/Homer	1e-100	-2.315e+02	0.0000	2838.0	8.03%	1657.4	5.30%
8		PU.1:IRF8(ETS:IRF)/pDC-Irf8-ChIP-Seq(GSE66899)/Homer	1e-97	-2.249e+02	0.0000	1261.0	3.57%	583.8	1.87%
9		Fos(bZIP)/TSC-Fos-ChIP-Seq(GSE110950)/Homer	1e-92	-2.141e+02	0.0000	2605.0	7.37%	1517.0	4.85%
10		JunB(bZIP)/DendriticCells-Junb-ChIP-Seq(GSE36099)/Homer	1e-92	-2.122e+02	0.0000	2424.0	6.86%	1390.1	4.45%
11		Fosl2(bZIP)/3T3L1-Fosl2-ChIP-Seq(GSE56872)/Homer	1e-87	-2.010e+02	0.0000	1440.0	4.07%	724.7	2.32%
12		IRF1(IRF)/PBMC-IRF1-ChIP-Seq(GSE43036)/Homer	1e-84	-1.955e+02	0.0000	1122.0	3.17%	523.1	1.67%

Rank	Motif	Name	P-value	log P-pvalue	q-value (Benjamini)	# Target Sequences with Motif	% of Targets Sequences with Motif	# Background Sequences with Motif	% of Background Sequences with Motif
1		Oct11(POU,Homeobox)/NCIH1048-POU2F3-ChIP-seq(GSE115123)/Homer	1e-1390	-3.202e+03	0.0000	6161.0	13.70%	1895.6	4.23%
2		Brn1(POU,Homeobox)/NPC-Brn1-ChIP-Seq(GSE35496)/Homer	1e-1115	-2.567e+03	0.0000	5340.0	11.87%	1723.5	3.85%
3		Oct2(POU,Homeobox)/Bcell-Oct2-ChIP-Seq(GSE21512)/Homer	1e-1071	-2.467e+03	0.0000	5540.0	12.32%	1884.6	4.21%
4		Oct4(POU,Homeobox)/mES-Oct4-ChIP-Seq(GSE11431)/Homer	1e-993	-2.288e+03	0.0000	6773.0	15.06%	2736.7	6.11%
5		Oct6(POU,Homeobox)/NPC-Pou3f1-ChIP-Seq(GSE35496)/Homer	1e-840	-1.936e+03	0.0000	5807.0	12.91%	2346.5	5.24%
6		Fra2(bZIP)/Striatum-Fra2-ChIP-Seq(GSE43429)/Homer	1e-231	-5.325e+02	0.0000	3211.0	7.14%	1726.0	3.85%
7		Fra1(bZIP)/BT549-Fra1-ChIP-Seq(GSE46166)/Homer	1e-228	-5.251e+02	0.0000	3725.0	8.28%	2117.0	4.73%
8		Fos(bZIP)/TSC-Fos-ChIP-Seq(GSE110950)/Homer	1e-226	-5.205e+02	0.0000	3937.0	8.75%	2283.0	5.10%
9		OCT4-SOX2-TCF-NANOG(POU,Homeobox,HMG)/mES-Oct4-ChIP-Seq(GSE11431)/Homer	1e-218	-5.028e+02	0.0000	2135.0	4.75%	996.9	2.23%
10		JunB(bZIP)/DendriticCells-Junb-ChIP-Seq(GSE36099)/Homer	1e-205	-4.736e+02	0.0000	3594.0	7.99%	2083.2	4.65%
11		Atf3(bZIP)/GBM-ATF3-ChIP-Seq(GSE33912)/Homer	1e-196	-4.522e+02	0.0000	4199.0	9.34%	2583.2	5.77%
12		Fosl2(bZIP)/3T3L1-Fosl2-ChIP-Seq(GSE56872)/Homer	1e-195	-4.491e+02	0.0000	2237.0	4.97%	1115.3	2.49%

Rank	Motif	Name	P-value	log P-pvalue	q-value (Benjamini)	# Target Sequences with Motif	% of Targets Sequences with Motif	# Background Sequences with Motif	% of Background Sequences with Motif
1		NeuroD1(bHLH)/Islet-NeuroD1-ChIP-Seq(GSE30298)/Homer	1e-117	-2.700e+02	0.0000	707.0	21.24%	3780.7	8.29%
2		Ascl1(bHLH)/NeuralTubes-Ascl1-ChIP-Seq(GSE55840)/Homer	1e-113	-2.618e+02	0.0000	1125.0	33.80%	7952.7	17.43%
3		Atoh1(bHLH)/Cerebellum-Atoh1-ChIP-Seq(GSE22111)/Homer	1e-100	-2.323e+02	0.0000	819.0	24.61%	5164.8	11.32%
4		DLX2(Homeobox)/BasalGanglia-Dlx2-ChIP-seq(GSE124936)/Homer	1e-94	-2.172e+02	0.0000	1081.0	32.48%	8045.8	17.64%
5		BHLHA15(bHLH)/NIH3T3-BHLHB8.HA-ChIP-Seq(GSE119782)/Homer	1e-90	-2.087e+02	0.0000	948.0	28.49%	6737.8	14.77%
6		Lhx2(Homeobox)/HFSC-Lhx2-ChIP-Seq(GSE48068)/Homer	1e-87	-2.026e+02	0.0000	826.0	24.82%	5559.0	12.19%
7		En1(Homeobox)/SUM149-EN1-ChIP-Seq(GSE120957)/Homer	1e-87	-2.021e+02	0.0000	1201.0	36.09%	9594.6	21.03%
8		MyoG(bHLH)/C2C12-MyoG-ChIP-Seq(GSE36024)/Homer	1e-85	-1.971e+02	0.0000	759.0	22.81%	4953.7	10.86%
9		NeuroG2(bHLH)/Fibroblast-NeuroG2-ChIP-Seq(GSE75910)/Homer	1e-85	-1.970e+02	0.0000	998.0	29.99%	7421.6	16.27%
10		Nkx6.1(Homeobox)/Islet-Nkx6.1-ChIP-Seq(GSE40975)/Homer	1e-85	-1.960e+02	0.0000	1532.0	46.03%	13619.2	29.86%
11		NF1-halfsite(CTF)/LNCaP-NF1-ChIP-Seq(Unpublished)/Homer	1e-84	-1.955e+02	0.0000	1078.0	32.39%	8314.3	18.23%
12		DLX5(Homeobox)/BasalGanglia-Dlx5-ChIP-seq(GSE124936)/Homer	1e-84	-1.954e+02	0.0000	674.0	20.25%	4151.4	9.10%
13		Twist2(bHLH)/Myoblast-Twist2.Ty1-ChIP-Seq(GSE127998)/Homer	1e-80	-1.856e+02	0.0000	1090.0	32.75%	8592.6	18.84%
14		LHX9(Homeobox)/Hct116-LHX9.V5-ChIP-Seq(GSE116822)/Homer	1e-78	-1.807e+02	0.0000	956.0	28.73%	7193.3	15.77%
15		DLX1(Homeobox)/BasalGanglia-Dlx1-ChIP-seq(GSE124936)/Homer	1e-78	-1.803e+02	0.0000	963.0	28.94%	7275.9	15.95%

Figure S3. Enriched motifs and stem-associated features of chromatin-informed SCLC programs, related to Figure 3

A. HOMER-enriched transcription factor motifs for SCLC-IM, SCLC-SL, and SCLC-NE programs.
B. Boxplot showing promoter accessibility of canonical stem -associated marker genes. Center line indicates the median; box limits indicate the first and third quartiles; whiskers represent 1.5 × IQR; points represent individual samples. Tukey's multiple comparison test; * $p < 0.05$, ** $p < 0.005$, *** $p < 0.0001$

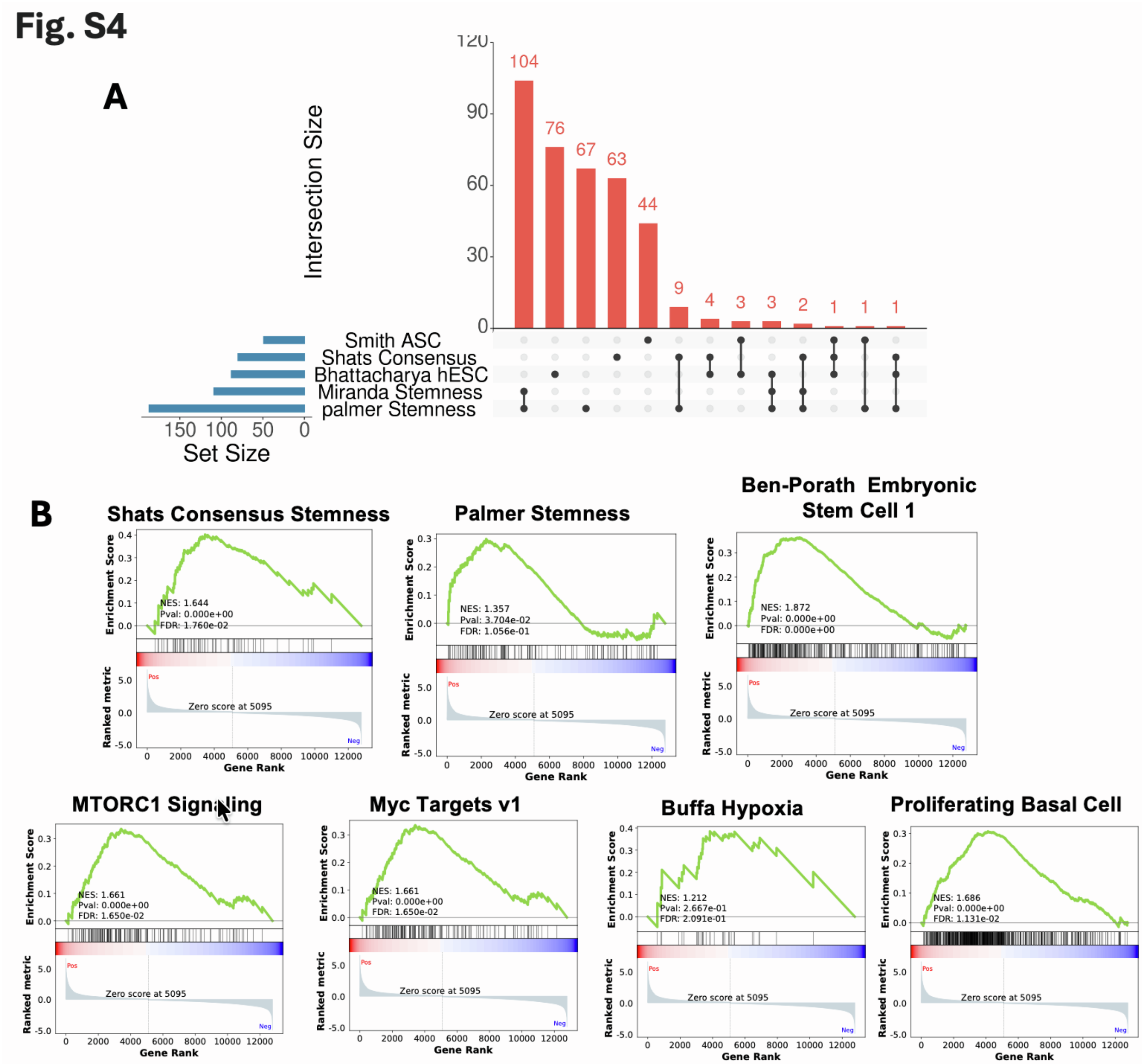

Figure S4. Stemness and metabolic features define the chromatin-informed SCLC-SL programs, related to Figure 4

A. UpSet plot depicting the degree of overlap among the stemness gene signature considered in the present study.
B. Gene set enrichment analysis shows multiple stemness signature along with metabolic signatures are exclusively high in SCLC-SL group compared to SCLC-IM and SCLC-NE.

Fig. S5

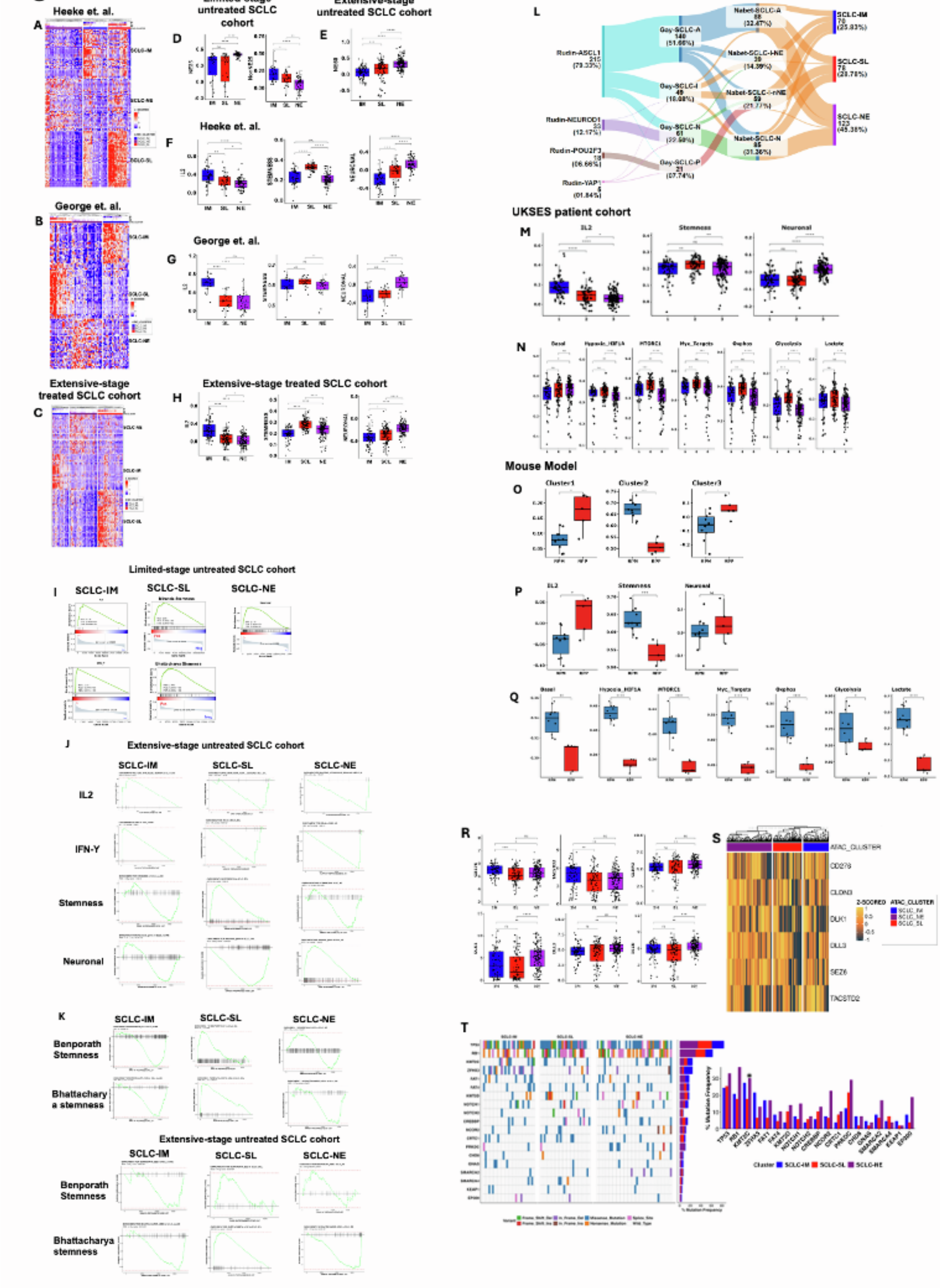
Heeke et. al.
George et. al.
Extensive-stage treated SCLC cohort
Limited-stage untreated SCLC cohort
Extensive-stage untreated SCLC cohort
Heeke et. al.
George et. al.
Extensive-stage treated SCLC cohort
Limited-stage untreated SCLC cohort
SCLC-IM
SCLC-SL
SCLC-NE
Extensive-stage untreated SCLC cohort
IL2
IFN-Y
Stemness
Neuronal
Benporath Stemness
Bhattacharya stemness
Extensive-stage untreated SCLC cohort
UKSES patient cohort
Mouse Model
Cluster1
Cluster2
Cluster3
ATAC_CLUSTER
CD276
CLDN3
DLK1
DLL3
SEZ6
TACSTD2
% Mutation Frequency
Cluster SCLC-IM SCLC-SL SCLC-NE

Figure S5. Cross-cohort identification of chromatin-informed SCLC program and associated features, related to Figures 5

A-C. Heatmap showing relative expression of chromatin accessibility-defined program signature genes in Heeke et al.[51], George et al.[27], and NCI datasets. Top annotations indicate mean expression of signature genes derived from chromatin-based clusters

D, E. NE score in the chromatin-derived SCLC program identified in the limited stage[14] and extensive-stage [13]SCLC datasets. Center line indicates the median; box limits indicate the first and third quartiles; whiskers represent 1.5 × IQR; points represent individual samples. Statistical comparisons were performed using Tukey's multiple comparison test; * $p < 0.05$, **$p < 0.005$, ***$p < 0.0001$

F-H. Expression of IL2, stemness, and neuronal signatures in Heeke et al.[51], George et al.[27], and NCI Thomas datasets. Center line indicates the median; box limits indicate the first and third quartiles; whiskers represent 1.5 × IQR; points represent individual samples. Statistical comparisons were performed using Tukey's multiple comparison test; * $p < 0.05$, **$p < 0.005$, ***$p < 0.0001$

I.IL2, IFN-gamma, Stemness and neuronal pathways enrichment (GSEA-based) in SCLC-IM, SCLC-SL, and SCLC-NE in LS-SCLC tumors.

J. IL2, IFN-gamma, stemness and neuronal pathways enrichment (GSEA-based) in SCLC-IM, SCLC-SL, and SCLC-NE in ES-SCLC tumors.

K. Additional validation of stemness enrichment in SCLC-SL using multiple additional stem signatures[43-46] (GSEA-based) in chromatin accessibility defined SCLC tumors (LS-SCLC and ES-SCLC)

L. Sankey diagram depicting the reclassification of prior SCLC classifications (Rudin et al., Gay et al., Nabet et al) using the chromatin-based classification in extensive-stage SCLC patient tumors[13].

M. Boxplot showing pathway level activity of IL2, stemness, and neuronal signatures in UKSES cohort[57] (n=226). Center line indicates the median; box limits indicate the first and third quartiles; whiskers represent 1.5 × IQR; points represent individual samples. Statistical comparisons were performed using Tukey's multiple comparison test; * $p < 0.05$, **$p < 0.005$, ***$p < 0.0001$

N. Boxplot showing enrichment of unique biological programs in SCLC-SL (Cluster2) compared to the other, SCLC-IM (Cluster1) and SCLC-NE (Cluster3) in UKSES cohort. Center line indicates the median; box limits indicate the first and third quartiles; whiskers represent 1.5 × IQR; points represent individual samples. Statistical comparisons were performed using Tukey's multiple comparison test; * $p < 0.05$, **$p < 0.005$, ***$p < 0.0001$

O. Boxplots showing enrichment of chromatin-derived cluster signature in the SCLC mouse model transcriptomic dataset[58] (RPM and RPP). RPM mouse model shows enrichment of the Cluster2 (stem-like, SCLC-SL) signature, while significantly de-enriched for both Cluster1 and Cluster3 signature, indicating consistent with the SCLC-SL program. Center line indicates the median; box limits indicate the first and third quartiles; whiskers represent 1.5 × IQR; points represent individual samples. Statistical comparisons were performed using Tukey's multiple comparison test; * $p < 0.05$, **$p < 0.005$, ***$p < 0.0001$

P. Pathway level activity of the key functional programs in RPM and RPP mouse model. RPM model shows significant enrichment of stemness pathway while de-enriched for both IL2 and Neuronal programs. Center line indicates the median; box limits indicate the first and third quartiles; whiskers represent 1.5 × IQR; points represent individual samples. Statistical comparisons were performed using Tukey's multiple comparison test; * $p < 0.05$, **$p < 0.005$, ***$p < 0.0001$

Q. Additional pathway analysis demonstrating increased activity of basal cell, hypoxia (HIF1A), MTORC1 signaling, MYC targets, oxidative phosphorylation, glycolysis, and lactate-associated pathways in RPM tumors, consistent with a metabolically reprogrammed, stem-like phenotype. Center line indicates the median; box limits indicate the first and third quartiles; whiskers represent 1.5 × IQR; points represent individual samples. Statistical comparisons were performed using Student's t.test *$p < 0.05$, **$p < 0.01$, ***$p < 0.001$, ****$p < 0.0001$; ns, not significant

R. Expression levels of clinically relevant surface targets across chromatin accessibility informed programs in extensive stage disease[13]. Center line indicates the median; box limits indicate the first and third quartiles; whiskers represent 1.5 × IQR; points represent individual samples. Statistical comparisons were performed using Tukey's multiple comparison test; * $p < 0.05$, **$p < 0.005$, ***$p < 0.0001$

S. Comprehensive expression profiling of surface immune targets across chromatin-informed programs in extensive stage SCLC[13] following stromal deconvolution.

T. Mutation landscape and frequency bar plot showing significant enrichment of ZFHX3 mutation in SCLC-IM in LS-SCLC[14]. Statistical comparisons were performed using Fisher's exact test; * $p < 0.05$, **$p < 0.005$, ***$p < 0.0001$
GSEA, gene set enrichment analysis; NE, neuroendocrine; LS, Limited stage; ES, extensive stage

Fig. S6

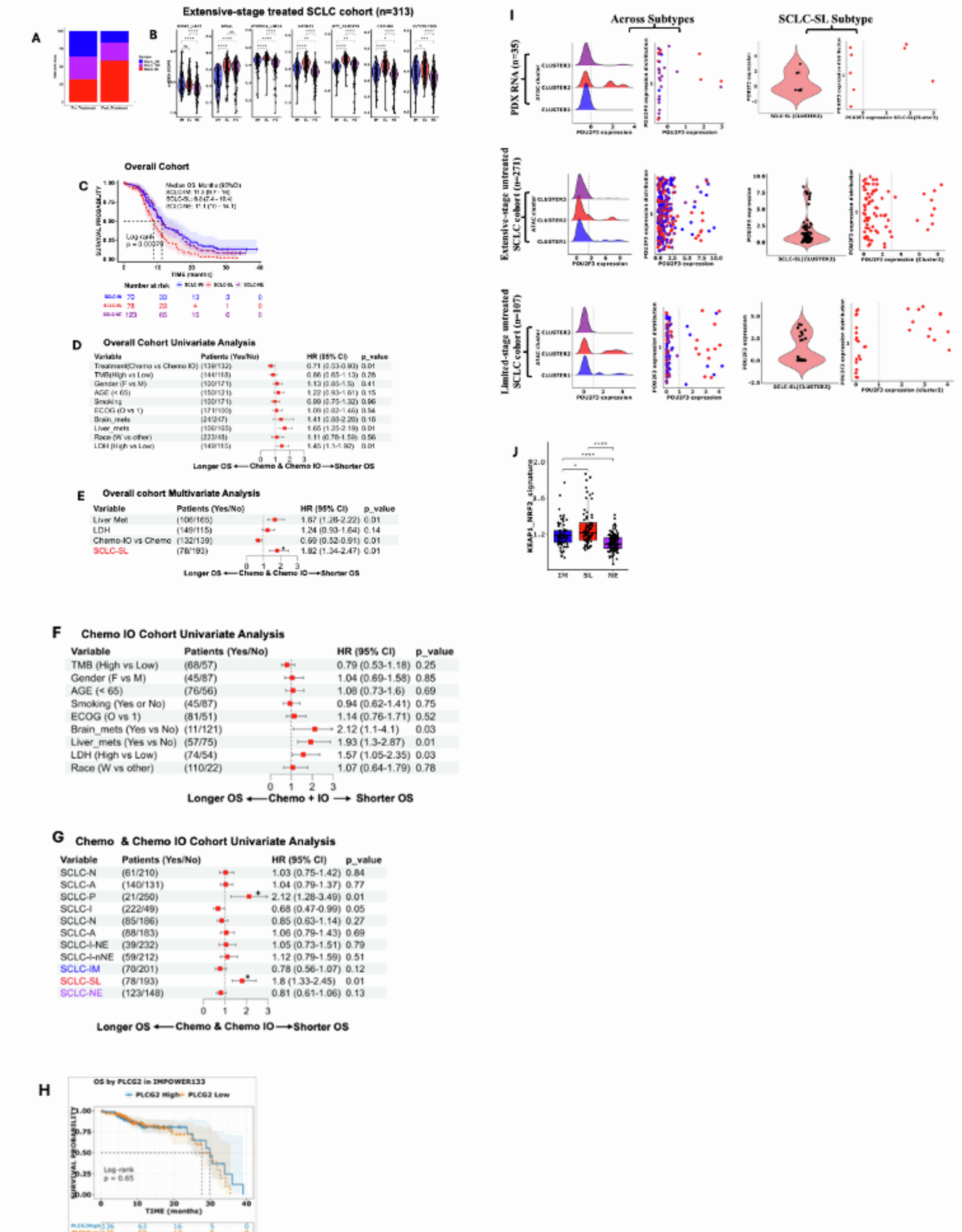

Extensive-stage treated SCLC cohort (n=313)
A
B
C
Overall Cohort
Log-rank p = 0.00079
TIME (months)
Number at risk
D
Overall Cohort Univariate Analysis
Longer OS ← Chemo & Chemo IO → Shorter OS
E
Overall cohort Multivariate Analysis
Variable
Patients (Yes/No)
HR (95% CI)
p_value
Liver Met
(106/165)
1.67 (1.26-2.22)
0.01
LDH
(149/115)
1.24 (0.93-1.64)
0.14
Chemo-IO vs Chemo
(132/139)
0.69 (0.52-0.91)
0.01
SCLC-SL
(78/193)
1.82 (1.34-2.47)
0.01
Longer OS ← Chemo & Chemo IO → Shorter OS
F
Chemo IO Cohort Univariate Analysis
Variable
Patients (Yes/No)
HR (95% CI)
p_value
TMB (High vs Low)
(68/57)
0.79 (0.53-1.18)
0.25
Gender (F vs M)
(45/87)
1.04 (0.69-1.58)
0.85
AGE (< 65)
(76/56)
1.08 (0.73-1.6)
0.69
Smoking (Yes or No)
(45/87)
0.94 (0.62-1.41)
0.75
ECOG (O vs 1)
(81/51)
1.14 (0.76-1.71)
0.52
Brain_mets (Yes vs No)
(11/121)
2.12 (1.1-4.1)
0.03
Liver_mets (Yes vs No)
(57/75)
1.93 (1.3-2.87)
0.01
LDH (High vs Low)
(74/54)
1.57 (1.05-2.35)
0.03
Race (W vs other)
(110/22)
1.07 (0.64-1.79)
0.78
Longer OS ← Chemo + IO → Shorter OS
G
Chemo & Chemo IO Cohort Univariate Analysis
Variable
Patients (Yes/No)
HR (95% CI)
p_value
SCLC-N
(61/210)
1.03 (0.75-1.42)
0.84
SCLC-A
(140/131)
1.04 (0.79-1.37)
0.77
SCLC-P
(21/250)
2.12 (1.28-3.49)
0.01
SCLC-I
(222/49)
0.68 (0.47-0.99)
0.05
SCLC-N
(85/186)
0.85 (0.63-1.14)
0.27
SCLC-A
(88/183)
1.06 (0.79-1.43)
0.69
SCLC-I-NE
(39/232)
1.05 (0.73-1.51)
0.79
SCLC-I-nNE
(59/212)
1.12 (0.79-1.59)
0.51
SCLC-IM
(70/201)
0.78 (0.56-1.07)
0.12
SCLC-SL
(78/193)
1.8 (1.33-2.45)
0.01
SCLC-NE
(123/148)
0.81 (0.61-1.06)
0.13
Longer OS ← Chemo & Chemo IO → Shorter OS
H
OS by PLCG2 in IMPOWER133
PLCG2 High
PLCG2 Low
SURVIVAL PROBABILITY
Log-rank p = 0.65
TIME (months)
I
Across Subtypes
SCLC-SL Subtype
PDX RNA (n=35)
Extensive-stage untreated SCLC cohort (n=271)
Limited-stage untreated SCLC cohort (n=107)
CLUSTER3
CLUSTER2
CLUSTER1
POU2F3 expression
J
KEAP1_NRF2_signature
IM
SL
NE

Figure S6. Prognostic and molecular features of chromatin-informed SCLC programs, related to Figure 6

A. Proportions of chromatin accessibility-informed programs for paired samples (NCI dataset) from the same patients across two time points along the treatment course.

B. Violin plot showing enrichment of unique biological programs in SCLC-SL, compared to the others (SCLC-IM and SCLC-NE) in NCI dataset. Violin plots show the distribution of ssGSEA scores; points represent individual patient tumors. Statistical comparisons were performed using Tukey's multiple comparison test. *$p < 0.05$, **$p < 0.005$, ***$p < 0.0001$.

C. Kaplan-Meier survival analysis of patients from the IMpower133 trial[26] with extensive-stage SCLC[13] treated with chemoimmunotherapy or chemotherapy (n = 271), stratified by the chromatin-based features.

D. Forest plot of univariate hazard ratios and clinical variables in overall cohort for ES-SCLC dataset[13] treated with chemoimmunotherapy or chemotherapy.

E. Multivariate cox regression analysis adjusting for liver metastases, LDH level, and treatment (chemoimmunotherapy or chemotherapy)

F. Forest plot of univariate hazard ratios and clinical variables in chemoimmunotherapy cohort for ES-SCLC dataset[13] (n=132 patients)

G. Forest plot of univariate hazard ratios for chromatin-informed programs and prior SCLC classifications from (Gay et al. and Nabet et al.) in overall cohort for ES-SCLC dataset[13] (n=271).

H. Kaplan-Meier overall survival analysis of Impower133 cohort (n = 271), stratified by PLCG2 expression.

I. POU2F3 expression across chromatin-defined SCLC program in PDX, limited-stage[14], and extensive-stage[13] SCLC cohorts. POU2F3 expression is heterogeneous within SCLC-SL and not exclusive to this subtype

J. KEAP1/NRF2 pathway signature in SCLC-SL subtype in the extensive-stage[13] SCLC cohort. Center line indicates the median; box limits indicate the first and third quartiles; whiskers represent 1.5 × IQR; points represent individual samples. Statistical comparisons were performed using Tukey’s multiple comparison test; * $p < 0.05$, **$p < 0.005$, ***$p < 0.0001$
